# Effects of Carbohydrate Mouth Rinse on Human Capacity and Performance—A Systematic Review and Meta-Analysis of 90 Controlled Trials

**DOI:** 10.3390/nu18183099

**Published:** 2026-09-21

**Authors:** Constantine Zalagkitis, Anastasia Kraniotaki, Nikolaos Simitzis, Konstantinos Dallas, Athanasios Moustogiannis, Eirini Chatzinikita, Giorgos Paradisis, Roxane Tenta, Maria Maridaki, Aristeidis Veskoukis, George Metsios, Anastassios Philippou, Costas Chryssanthopoulos, Petros C. Dinas

**Affiliations:** 1 Department of Nutrition and Dietetics, School of Physical Education, Sport Science and Dietetics, University of Thessaly, 421 32 Trikala, Greece; zaldean@yahoo.gr (C.Z.); anastasiakraniotaki@gmail.com (A.K.); nikolassimit@gmail.com (N.S.); veskoukis@gmail.com (A.V.); g.metsios@uth.gr (G.M.); 2 Department of Physiology, Medical School, National and Kapodistrian University of Athens, 75 Micras Asias, 115 27 Athens, Greece; kwnstantinos.dallas@gmail.com (K.D.); moustogi@gmail.com (A.M.); echatzin@med.uoa.gr (E.C.); tfilipou@med.uoa.gr (A.P.); chryssan@phed.uoa.gr (C.C.); 3 Faculty of Physical Education and Sport Science, National and Kapodistrian University of Athens, 172 37 Athens, Greece; gparadi@phed.uoa.gr (G.P.); mmarida@phed.uoa.gr (M.M.); 4 Department of Nutrition and Dietetics, School of Health Science and Education, Harokopio University, 70 El. Venizelou, 176 76 Kallithea, Greece; rtenta@hua.gr

**Keywords:** CHO, workload, muscular endurance, mouth rinsing

## Abstract

**Objective**: The aim of the current systematic review and meta-analysis was to examine the acute effects of carbohydrate (CHO) mouth rinse on exercise capacity and performance in humans of any health and fitness status. **Methods**: We registered our systematic review with PROSPERO (CRD42018085308). Eligibility criteria included adult humans > 18 years old at any health and fitness status undertaking any CHO mouth rinse prior and during of any type of acute exercise session. **Results**: We included 90 crossover design-controlled trials. We found improvements in distance covered (Hedges’ g = 0.17; 95% CI 0.03 to 0.30, *p* = 0.02; τ^2^ = 0.00; I^2^ = 0%; Q(14) = 9.61, *p* > 0.05), performance time (Hedges’ g = −0.15; 95% CI −0.25 to −0.05, *p* < 0.01; τ^2^ = 0.008; I^2^ = 9.3%; Q(34) = 76.48, *p* < 0.01), speed performance (Hedges’ g = 0.17; 95% CI 0.05 to 0.30, *p* < 0.01; τ^2^ ≈ 0; I^2^ = 0%; Q(23) = 22.73, *p* > 0.05), workload (Hedges’ g = 0.25; 95% CI 0.08 to 0.41, *p* < 0.01, τ^2^ = 0; I^2^ = 0%; (Q(9) = 7.37, *p* > 0.05) and maximum voluntary contraction (MVC) (Hedges’ g = 0.18, 95% CI 0.02 to 0.34, *p* = 0.03; τ^2^ = 0; I^2^ = 0%; (Q(10) = 7.73, *p* > 0.05). No effect of CHO mouth rinse on muscular endurance, maximum power output, or maximum oxygen consumption (VO_2_max) was found (*p* > 0.05). **Conclusions**: The available evidence suggests that CHO mouth rinse can produce a small, outcome-dependent ergogenic effect in distance covered, performance time and speed performance as well as small improvements in workload, with less robust evidence for MVC—evidence that mainly applies to male participants. The practical importance of these findings is likely to vary according to the exercise context and competitive context.

## 1. Introduction

Carbohydrate (CHO) ingestion has been recognized as an effective nutritional strategy for enhancing exercise performance by maintaining blood glucose concentration and preserving endogenous glycogen stores during prolonged exercise [1]. However, other evidence suggests that the ergogenic effects of CHO are not solely attributable to their metabolic availability and simply rinsing the mouth with a CHO solution, without swallowing it, can improve exercise performance, which indicates the involvement of central neural mechanisms independent of CHO oxidation [2]. The CHO mouth rinse typically involves rinsing the oral cavity with a CHO solution, for approximately 5–10 s before expectoration. This strategy activates CHO-sensitive receptors located in the oral cavity, which stimulate brain regions involved in reward, motivation, and motor control, including the anterior cingulate cortex and striatum [3]. Functional magnetic resonance imaging has shown that oral CHO exposure activates these brain areas independently of sweetness perception, suggesting that the ergogenic effects are mediated through central nervous system pathways rather than through metabolic substrate provision [3]. In this light, CHO mouth rinse increases motivation, reduces the perception of effort, and ultimately allows athletes to sustain higher exercise intensities [4]. As a result, CHO mouth rinse may improve exercise performance probably without changing circulating glucose or insulin concentrations.

We located five previous systematic reviews that examined the effects of CHO mouth rinse on exercise performance. In 2013, evidence showed that CHO mouth rinse improved endurance performance by 1.5% to 11.6% in cycling and running time trials [5]. Similarly, in 2019, another systematic review showed significant, but small improvements in power output and time to complete the cycling trial [6]. Maltodextrin mouth rinse also showed a small ergogenic effect in multiple exercise modes in 2022 [7]. However, Oliveira-Silva et al. in 2023 revealed no effect of CHO mouth rinse on maximal strength and limited evidence of an effect on muscular endurance [8]. Similarly, in 2026, Suen et al. showed no effect of CHO mouth rinse on high-intensity interval training performance [9]. These findings indicate that the effect of CHO mouth rinse on exercise performance and capacity shows inconsistencies and depends on exercise mode and probably on nutritional status [10]. Given that CHO mouth rinse continues to be an active area of investigation, with ongoing research aimed at identifying the exercise conditions, athlete populations, and physiological mechanisms that maximize its effectiveness, there is a need for an accumulation and analysis of the current evidence. Also, CHO mouth rinse represents a practical, low-cost, and non-invasive nutritional strategy for enhancing exercise performance and a better understanding of the factors influencing its efficacy may help optimize its application across different sporting disciplines and competitive settings. Therefore, the aim of the current systematic review and meta-analysis was to examine the acute effects of CHO mouth rinse on exercise capacity and performance in humans at any health and fitness status.

## 2. Materials and Methods

We conducted a systematic review and meta-analysis according to the Preferred Reporting Items for Systematic Reviews and Meta-Analyses (PRISMA) guidelines [11]. We registered the protocol with the International Prospective Register of Systematic Reviews (PROSPERO) database (registration number: CRD42018085308) [12].

### 2.1. Eligibility Criteria

We used the population, intervention, comparator, outcome, study design (PICOS) method to set the eligibility criteria, as follows:

- Population: Adult humans > 18 years old at any health and fitness status. Studies in children and adolescents, as well as in animals, were excluded.
- Intervention: Any CHO mouth rinse prior and during any type of acute exercise session. Studies that included consumption of CHO and/or other supplements immediately prior to and during the intervention, were excluded. Studies that included mouth rinse other than CHO, were also excluded. Studies that examined chronic effects of CHO mouth rinse on exercise capacity and performance and studies that included consumption of a meal less than two hours prior to intervention, were excluded.
- Comparator: Post-acute exercise intervention measurements of a control group or a control situation, in the case of a crossover design. Accepted control groups or situations included mouth rinse with a placebo, water or no mouth rinse.
- Outcomes: Strength (measured in kilograms/pounds via 1 repetition maximum test and/or load lifted), power (measured in watts), exercise performance in time (measured via any sport such as cycling and running), exercise performance in distance (measured in kilometres/miles), total intensity exercise performance (measured in intensity percentage), muscular endurance (number of repetitions × load lifted and/or time × load lifted), and isometric force/torque (measured via dynamometer or electromyography). Studies that included CHO mouth rinse without exercise capacity and performance measurements were excluded. Studies that included measurements of fatigue, and psychological and cognitive performance measurements were out of the scope of the current systematic review.
- Study design: We accepted controlled trials (CTs) and we excluded epidemiological, cross-sectional, and case–control studies; case reports; case series studies; reviews; editorials; conference abstracts; magazine articles; and grey literature.

### 2.2. Search and Data Sources

We used PICOS to employ our search strategy. We searched PubMed, Ovid and Sport Discus databases from their inception to December 2025. The searching key word algorithms for all databases can be found in the supplement. The searches were performed by CZ and were checked by PCD.

### 2.3. Selection Process

Two independent investigators (CZ and AK) performed the selection process, while PCD acted as a referee. Initially, we used the EndNote software to remove the duplicated publications, reviews, conference abstracts, editorials, protocols, reports and grey literature from the total number of retrieved publications. Subsequently, CZ and AK independently selected the eligible publications. Finally, NS screened the reference lists of the eligible publications to identify potential publications that fulfilled the eligibility criteria and did not appear in the initial searching procedure.

### 2.4. Data Extraction

CZ, AK and NS extracted the data from the eligible studies as follows: (a) First author and year of publication, (b) study design, location and funding, (c) participants’ characteristics (age, gender, body mass index), (d) exercise/physical activity intervention, (e) nutrition/diet status, (f) mouth rinse intervention, and (g) outcomes. Finally, CZ and PCD extracted the data suitable for meta-analysis in relevant Excel files.

### 2.5. Risk of Bias Assessment

Two independent investigators (NS and CC) assessed the quality of the eligible studies, using the updated Risk of Bias 2 Cochrane library tool [13]. PCD acted as a referee in case of a disagreement between NS and CC.

### 2.6. Data Synthesis

Three eligible CTs [14,15,16] did not offer data for a meta-analysis and therefore, we used a narrative data synthesis for these studies. For the remaining 87 eligible CTs, random-effects model meta-analyses were employed due to heterogeneity in participants’ characteristics, exercise and mouth rinse interventions, duration of the intervention and tools of measurement for the variables of interest. In particular, we conducted continuous meta-analyses for the following outcomes: (a) muscular endurance (i.e., repetitions to failure), (b) maximum voluntary contraction (MVC), (c) distance covered during the exercise intervention, (d) performance time, (e) power output, (f) speed, (g) maximal oxygen consumption (VO_2_max) and (h) workload. In cases where a standard error was given instead of a standard deviation (SD), we converted it into SD using the equation: SD = standard error × √n [17]. When SD was not reported and could not be estimated, we imputed SD using the coefficient of variation (CV), following previously published methodology. We calculated the mean CV from the remaining studies (CV = SD/Mean) and applied it to the mean of the study lacking SD information to obtain an imputed SD [17,18]. Where pertinent, we calculated speed using the following equation: Speed = Distance/Performance time [19]. For 22 eligible CTs that reported data in figures [20,21,22,23,24,25,26,27,28,29,30,31,32,33,34,35,36,37,38,39,40,41], these were extracted via the WebPlotDigitizer (version 5.2, 2024; Ankit Rohatgi, Pacifica, CA, USA) software [42], which displays >13,000 citations in Google Scholar, has shown intercoder reliability of 99.7% and agreement of 98.7% with the values reported in the original research [43], and it has been suggested to be used in data synthesis of systematic reviews [44]. For the performance time outcome, we multiplied the mean value by −1 for nine eligible CTs [10,27,34,45,46,47,48,49,50], due to the direction of the time (i.e., the higher the better) used for measuring the effect value, which was different from the measurement time (i.e., the lower the better) used in the other eligible studies included in this particular meta-analysis [17].

In the eligible studies that reported more than one experimental CHO mouth rinse condition within the same participants, these experimental conditions were combined to obtain a single overall CHO mouth rinse intervention estimate for the pairwise meta-analysis. The combined mean was calculated as the sample-size-weighted mean of the individual experimental conditions, while an assumed correlation of r = 0.50, with sensitivity analyses using r = 0.25 and r = 0.75, was used to assess the robustness of the pooled estimates. The corresponding SD was calculated taking into account the within-participant correlation between conditions, following the equation: SDcombined = 1/k √((sum(i = 1)^k^ SD_i_^2^ + 2r sum(i < j) SD_i_ SD_j_)), where k is the number of experimental conditions, SDi and j are the SDs of the conditions, and r is the assumed within-participant correlation between conditions [17].

All meta-analyses were conducted in RStudio (version 4.6.1 (24 June 2026; Posit Software, PBC, Boston, MA, USA) using the metafor package [51]. Continuous outcomes were synthesized using standardized mean differences (SMD), expressed as Hedges’ g, to account for differences in the measurement scales used across studies [17]. Hedges’ g was used to provide a small-sample bias correction [52]. Random-effects meta-analyses were performed using restricted maximum likelihood (REML) to estimate between-study variance (τ^2^). Statistical heterogeneity was assessed using Cochran’s Q statistic and quantified using I^2^ [17,51]. Given that all the eligible studies were crossover design studies, the paired nature of observations was taken into account when estimating the variance of the intervention effect. Because the within-participant correlation was not reported in the eligible studies, the correlation was imputed to estimate the variance of the within-participant difference, following the recommendations of the *Cochrane Handbook* [17]. The primary analysis assumed a correlation of r = 0.50, with sensitivity analyses conducted using r = 0.25 and r = 0.75 to assess the robustness of the pooled estimates to the assumed correlation. To account for the within-participant correlation, the SD of the paired intervention–control difference was calculated according to the *Cochrane Handbook* as follows: SDdiff = √SD^2^ experimental + SD^2^ control – 2 × corr(r) × (SD experimental) × (SD control), where (r) represents the assumed within-participant correlation between the experimental and control conditions [17].

Considering the availability of the existing evidence in the eligible CTs, we performed subgroup analyses for the following subgroups: (a) exercise type, (b) CHO mouth rinse type, (c) concentration of CHO solution with cut-offs corresponding more closely to the concentrations commonly used in the literature (i.e., ≤8% vs. >8%) [5], and (d) fitness level of the population (i.e., athletes vs. non-athletes). Subgroup analyses that violated meta-analysis principles (i.e., included only one study), were not considered in the final interpretation [17]. Differences between subgroups were assessed using mixed-effects meta-regression and the QM test of moderators [51]. Potential small-study effects were assessed using funnel plots and Egger’s regression test for funnel-plot asymmetry [53].

### 2.7. Certainty of Evidence and Interpretation

We used the Grading of Recommendations Assessment, Development and Evaluation (GRADE) analysis to assess the quality of evidence in all meta-analyses of predefined outcomes, irrespective of statistical significance [54]. We considered risk of bias, inconsistency (heterogeneity), indirectness, imprecision and publication bias to reduce the quality of evidence, while we considered the magnitude of effect to increase the quality of evidence. The GRADE quality of evidence was defined as very low, low, moderate and high. For the interpretation of the results, we followed specific terminology as suggested by the *Cochrane Handbook*, by combining the quality of evidence we obtained from GRADE analysis and the magnitude of effect of each meta-analysis [17]. The magnitude of effect was expressed as <0.2 = trivial, 0.2–0.5 = small, 0.5–0.8 = moderate and >0.8 = large, according to Cohen’s d effect size [54].

## 3. Results

We provide a PRISMA checklist in the supplement Table S3.

### 3.1. Search and Selection Processes

**Figure 1:**
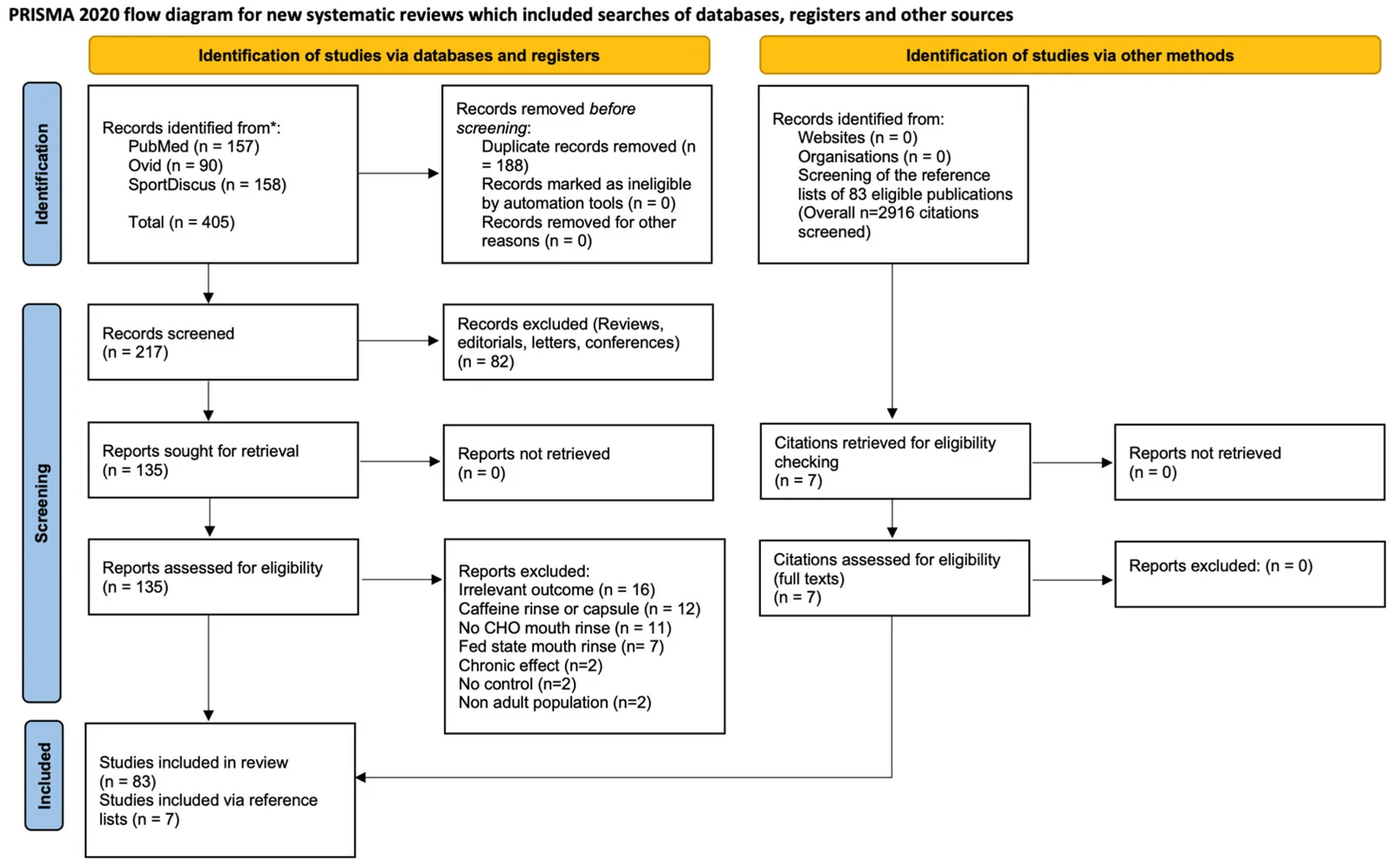
PRISMA flow diagram. * reporting the number of records identified from each database.

A PRISMA flow diagram is depicted in Figure 1. The search procedure retrieved a total of 405 publications (PubMed = 157, Ovid = 90, SportDiscus = 158). Among them, 188 publications were removed as duplicates, while the remaining 217 were screened for eligibility. Consequently, 82 publications were removed as reviews, letters, conference abstracts, etc., 16 were excluded due to irrelevant outcome, 12 due to they used caffeine as mouth rinse intervention, 11 not included a CHO mouth rinse, seven included a CHO mouth rinse in a fed state, two examined the chronic effect of CHO mouth rinse, two had no control group or situation and two examined non-adult populations. As such 83 CTs were deemed eligible. We then screened the reference lists of the 83 eligible CTs to determine eligibility for seven more publications. As a result, a total of 90 CTs were finally included in the systematic review.

### 3.2. Study Characteristics

Study characteristics can be found in the supplement (Table S1). The eligible CTs involved 1184 participants with a mean age of 25.06 years, of whom 96.45% were males. Twenty-seven of the eligible CTs involved athletes in their protocols, while 63 involved recreational athletes. Twenty-one eligible CTs used dextrose as a CHO mouth rinse solution; one study did not report the type of solution [16], one used fructose [55], one used maltose [56], seven used sucrose and the remaining eligible CTs used maltodextrin. For two pairs of studies—Ali 2016/Ali 2017 [34,57] and Ferreira 2018 [58,59]–we identified identical or highly similar participant characteristics, including sample size, age, height, and body mass, raising the possibility that the respective publications included the same or overlapping participant samples. However, as each report constituted a distinct publication with a unique DOI, the studies were retained in the systematic review. To minimize the risk of unit-of-analysis errors and avoid potential double counting of participants in the meta-analysis synthesis, studies suspected of containing overlapping samples were not included together in the same outcome-specific meta-analysis. Instead, data from each publication were included only in separate outcome meta-analyses, thereby ensuring that potentially duplicated participants did not contribute more than once to any single pooled effect estimate [17].

### 3.3. Risk of Bias

The risk of bias results are depicted in Table S2 and Figure S1. For the randomization process, 36 CTs showed some concerns, while 54 showed a low risk of bias. For the intervention assignment and intervention adherence only one CT [60] showed some concerns about risk of bias, as for the missing data [59], while 89 CTs revealed low risk of bias. For the outcome component, three CTs [29,31,61] showed some concerns and 87 had a low risk of bias. Finally, for the reported results component, 11 CTs displayed some concerns and 79 had a low risk of bias.

### 3.4. Data Synthesis, Certainty of Evidence and Interpretation

The three CTs that were included in the narrative data synthesis showed insufficient evidence that CHO mouth rinse enhances jump performance [15] and soccer skill-related performance [14], while one study showed that CHO mouth rinse improved MVC [16].

When multiple experimental CHO mouth-rinse conditions were evaluated, we used r = 0.50 as a primary correlation. As expected, the sensitivity analyses using r = 0.25 and r = 0.75 did not affect the combined means, while the resulting combined SDs differed by <20% from those obtained using r = 0.50 across all relevant outcomes. Given the limited sensitivity of the combined estimates to plausible alternative correlation assumptions, r = 0.50 was retained for the primary analyses on all occasions. Meta-analyses revealed that CHO mouth rinse did not improve muscular endurance compared with the control condition (Hedges’ g = 0.49, 95% CI −0.31 to 1.29, *p* > 0.05). Between-study heterogeneity was considerable (τ^2^ = 1.43; I^2^ = 95.7%), and Cochran’s Q test was statistically significant (Q(8) = 64.22, *p* < 0.01, Figure S2). Sensitivity analyses using alternative within-participant correlation coefficients produced the same overall conclusion. At r = 0.25, the pooled effect was Hedges’ g = 0.40 (95% CI −0.26 to 1.07, *p* > 0.05), with substantial heterogeneity (I^2^ = 93.9%; τ^2^ = 0.95). At r = 0.75, the pooled effect was Hedges’ g = 0.69 (95% CI −0.44 to 1.81, *p* > 0.05), with similarly high heterogeneity (I^2^ = 97.7%; τ^2^ = 2.85). Formal statistical assessment of funnel-plot asymmetry was considered inappropriate because only nine studies were included [17].

A visual inspection of the forest plot identified Pereira et al. (2020) as an influential study [40]. Leave-one-out sensitivity analysis showed that Pereira et al. (2020) [40] reduced the pooled effect to Hedges’ g = 0.07 (95% CI −0.16 to 0.30, *p* > 0.05, Figure S3) and markedly reduced heterogeneity to I^2^ = 47.3% (τ^2^ = 0.05; Q(7) = 13.43, *p* = 0.06). Therefore, Pereira et al. (2020) [40] accounted for a substantial proportion of the observed between-study heterogeneity and influenced the magnitude of the pooled estimate; however, its exclusion did not alter the pooled effect as this remained non-significant (*p* > 0.05).

**Figure 2:**
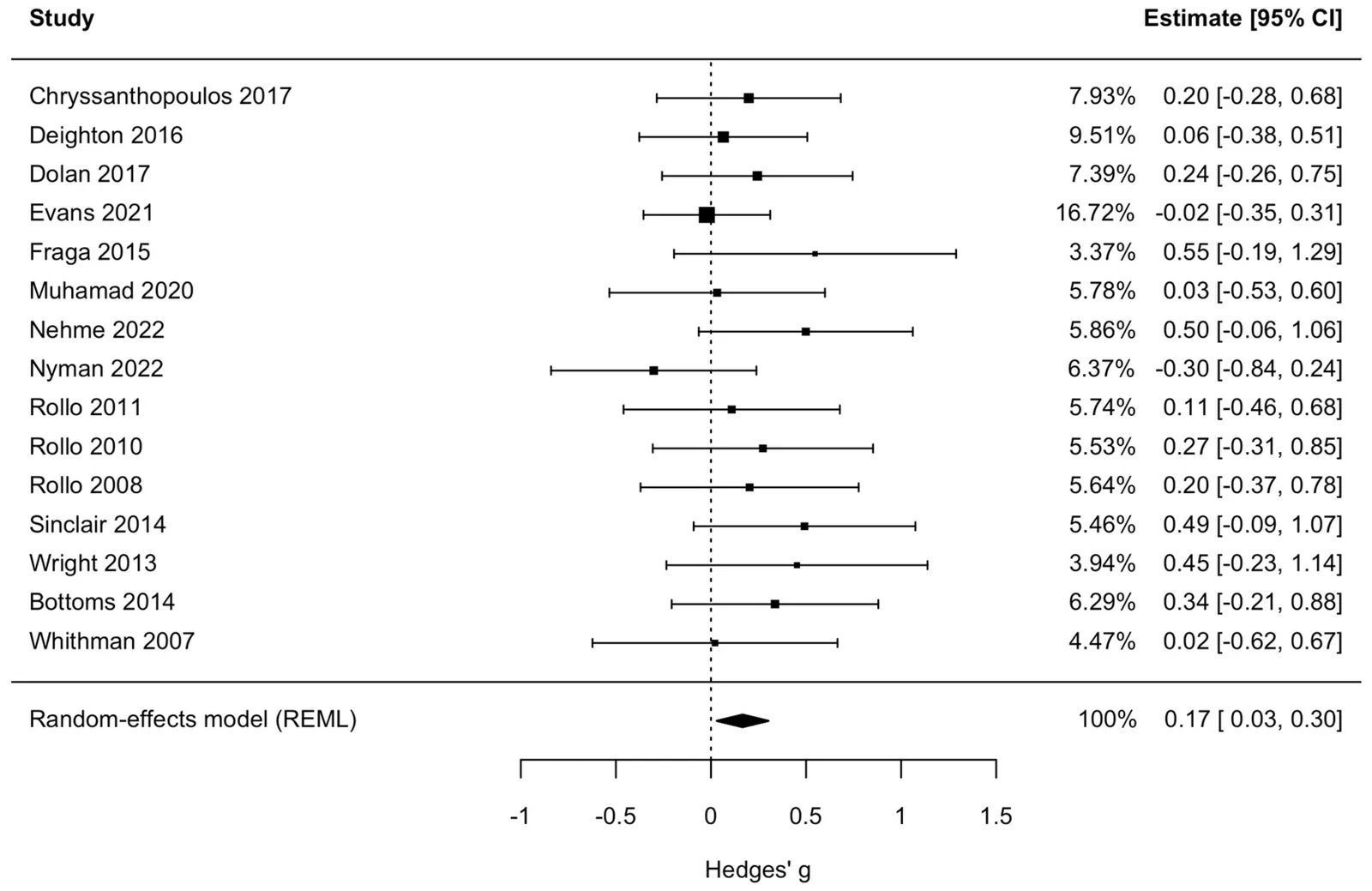
Forest plot distance covered [20,21,27,28,30,62,63,64,65,66,67,68,69,70,71].

Regarding the outcome of distance covered in any sport test (e.g., cycling, running, etc.) our meta-analysis showed a small but statistically significant positive effect compared with the control condition (Hedges’ g = 0.17, 95% CI 0.03 to 0.30, *p* = 0.02, Figure 2). There was no evidence of between-study heterogeneity (τ^2^ = 0; I^2^ = 0.0%), and Cochran’s Q test was non-significant (Q(14) = 9.61, *p* > 0.05). Sensitivity analyses examining different assumed within-participant correlations showed that the pooled effect remained statistically significant when correlations of r = 0.25 (Hedges’ g = 0.14, 95% CI 0.003 to 0.27, *p* = 0.04) and r = 0.75 (Hedges’ g = 0.24, 95% CI 0.08 to 0.40, *p* < 0.01) were assumed. Finally, Egger’s regression test indicated non-significant funnel-plot asymmetry (z = 1.63, *p* > 0.05, Figure S4).

Subgroup analysis according to exercise type showed significant positive effects for cycling (k = 2; Hedges’ g = 0.41, 95% CI 0.01 to 0.81, *p* = 0.04; I^2^ = 0%) and running (k = 10; Hedges’ g = 0.24, 95% CI 0.06 to 0.43, *p* < 0.01; I^2^ = 0%), whereas no significant effects were observed for hockey (k = 1; Hedges’ g = −0.30, 95% CI −0.84 to 0.24, *p* > 0.05) or walking (k = 2; Hedges’ g = 0.01, 95% CI −0.26 to 0.28, *p* > 0.05; I^2^ = 0%). However, the overall test for differences among exercise-type subgroups was not statistically significant (QM(3) = 6.31, *p* > 0.05). When studies were stratified according to CHO mouth rinse type, maltodextrin mouth rinsing was associated with a small significant improvement in performance (k = 8; Hedges’ g = 0.19, 95% CI 0.01 to 0.37, *p* = 0.03; I^2^ = 0%), whereas the effect of dextrose was not statistically significant (k = 7; Hedges’ g = 0.13, 95% CI −0.09 to 0.35, *p* > 0.05; I^2^ = 0%). Nevertheless, there was no evidence that the magnitude of the effect differed according to CHO mouth rinse type (QM(1) = 0.17, *p* > 0.05). Finally, subgroup analysis for fitness level showed a borderline statistically significant positive effect among recreationally active participants (k = 11; Hedges’ g = 0.16, 95% CI 0.0003 to 0.31, *p* = 0.05; I^2^ = 0%), whereas the effect among athletes was not statistically significant (k = 4; Hedges’ g = 0.22, 95% CI −0.16 to 0.60, *p* > 0.05; I^2^ = 43.5%). Importantly, the test for subgroup differences indicated no significant moderation by fitness level (QM(1) = 0.08, *p* > 0.05).

**Figure 3:**
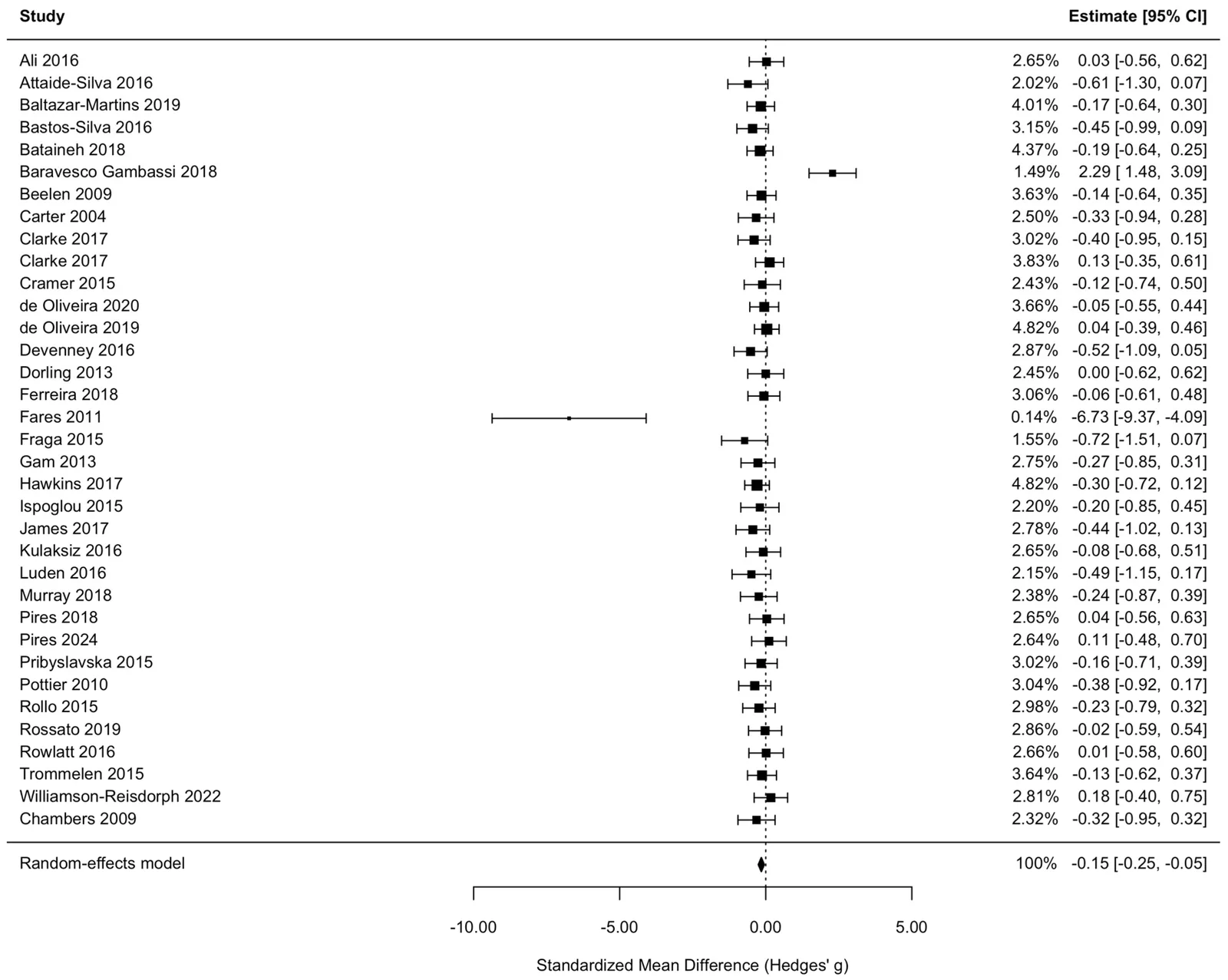
Forest plot performance time [2,3,10,27,29,34,35,45,46,47,48,49,50,55,58,60,72,73,74,75,76,77,78,79,80,81,82,83,84,85,86,87,88,89,90].

Performance time outcome showed improvement due to CHO mouth rinse with an overall Hedges’ *g* of −0.15 (95% CI −0.25 to −0.05, *p* < 0.01, Figure 3). Statistical heterogeneity was low (I^2^ = 9.3%, τ^2^ = 0.008), although Cochran’s Q test was statistically significant [Q(34) = 76.48, *p* < 0.01]. Sensitivity analyses examining different assumptions for the within-participant correlation showed that at r = 0.25, the pooled effect was g = −0.122 (95% CI −0.216 to −0.027, *p* < 0.01), with I^2^ = 0% and τ^2^ = 0.001. At r = 0.75, the pooled effect remained statistically significant and was somewhat larger, g = −0.216 (95% CI −0.342 to −0.090, *p* < 0.01), although heterogeneity increased to I^2^ = 38.4% (τ^2^ = 0.05).

A visual inspection of the forest plot identified Fares et al. (2011) [10] (Hedges’ g = −6.73) and Bavaresco Gambassi et al. (2018) [47] (Hedges’ g = 2.29) as influential studies. Leave-one-out sensitivity analysis showed that the pooled effect was not driven by any single study, with pooled estimates ranging from Hedges’ g = −0.19 to −0.14 following sequential exclusion of individual comparisons. Exclusion of Fares et al. (2011) [10] resulted in a pooled effect of Hedges’ g = −0.14 (95% CI −0.24 to −0.05, *p* < 0.01), whereas exclusion of Bavaresco Gambassi et al. (2018) [47] resulted in Hedges’ g = −0.19 (95% CI −0.28 to −0.09, *p* < 0.01). Importantly, the 95% confidence interval remained below the null value following exclusion of every individual comparison. Therefore, although these studies had comparatively large individual effect estimates, their exclusion did not alter the magnitude, direction, or statistical significance of the pooled effect.

Subgroup analysis according to exercise type showed no evidence that the pooled effect differed among cycling, running, and fencing [QM(2) = 0.274, *p* > 0.05]. The pooled effect was not statistically significant for cycling (k = 23; g = −0.166, 95% CI −0.369 to 0.037, *p* > 0.05; I^2^ = 61.3%) or running (k = 11; g = −0.141, 95% CI −0.296 to 0.013, *p* > 0.05; I^2^ = 0%). Fencing was represented by a single study (g = 0.013, 95% CI −0.577 to 0.603, *p* > 0.05) and was therefore not independently pooled. Similarly, the CHO mouth rinse type did not significantly moderate the overall effect [QM(3) = 1.219, *p* > 0.05]. The pooled effects were not statistically significant for dextrose (k = 5; g = −0.173, 95% CI −0.458 to 0.111, *p* > 0.05; I^2^ = 0%) or maltodextrin (k = 23; g = −0.151, 95% CI −0.342 to 0.040, *p* > 0.05; I^2^ = 60.1%). A statistically significant effect was observed for sucrose (k = 6; g = −0.208, 95% CI −0.415 to −0.0004, *p* = 0.05; I^2^ = 0%). Fructose was represented by a single study (g = 0.176, 95% CI −0.396 to 0.747, *p* > 0.05). However, given the non-significant test for subgroup differences, the significant within-sucrose estimate should not be interpreted as evidence that sucrose produces a different effect from the other CHO mouth rinse types.

Fitness level was also not a significant moderator [QM(1) = 1.069, *p* > 0.05]. Among athletes, a statistically significant pooled effect was observed (k = 12; g = −0.226, 95% CI −0.393 to −0.060, *p* < 0.01; I^2^ = 0%). In recreational participants, the pooled effect was not statistically significant (k = 23; g = −0.114, 95% CI −0.307 to 0.078, *p* > 0.05), with substantial within-subgroup heterogeneity (I^2^ = 61.8%, τ^2^ = 0.131). Subgroup analysis according to CHO mouth rinse solution concentration likewise provided no evidence of effect modification [QM(1) = 0.035, *p* > 0.05]. Solutions with concentrations ≤ 8% produced a statistically significant pooled effect (k = 29; g = −0.148, 95% CI −0.273 to −0.022, *p* = 0.02; I^2^ = 28.1%, τ^2^ = 0.033), whereas concentrations > 8% did not (k = 6; g = −0.170, 95% CI −0.397 to 0.057, *p* > 0.05; I^2^ = 0%). Finally, Egger’s regression test indicated significant funnel-plot asymmetry (z = −2.999, *p* < 0.01, Figure S5), suggesting the presence of small-study effects.

Our meta-analysis showed that CHO mouth rinse did not affect power output compared with the control condition (Hedges’ g = 0.008, 95% CI −0.13 to 0.14, *p* > 0.05, Figure S6). Between-study heterogeneity was moderate (I^2^ = 36.1%; τ^2^ = 0.057), and Cochran’s Q test indicated statistically significant heterogeneity (Q(33) = 69.47, *p* < 0.01). The findings were robust to alternative assumptions regarding the within-participant correlation. When a correlation of r = 0.25 was assumed, the pooled effect remained negligible and non-significant (Hedges’ g = 0.005, 95% CI −0.106 to 0.116, *p* > 0.05; I^2^ = 8.9%; τ^2^ = 0.010). Similarly, at r = 0.75, there was no significant intervention effect (Hedges’ g = 0.016, 95% CI −0.170 to 0.202, *p* > 0.05; I^2^ = 64.0%; τ^2^ = 0.188). Egger’s regression test provided evidence of funnel-plot asymmetry (z = 2.933, *p* < 0.01, Figure S7), with a limit estimate of −0.908 (95% CI −1.538 to −0.278).

CHO mouth rinse did not affect VO_2_max compared with the control condition (Hedges’ g = −0.003, 95% CI −0.18 to 0.18, *p* > 0.05, Figures S8 and S9). There was no evidence of between-study heterogeneity (I^2^ = 0%; τ^2^ ≈ 0), and Cochran’s Q test was non-significant (Q(9) = 11.08, *p* > 0.05). Sensitivity analyses with r = 0.25 showed that the pooled effect was Hedges’ g = −0.008 (95% CI −0.189 to 0.172, *p* > 0.05), with no observed heterogeneity (I^2^ = 0%; τ^2^ ≈ 0). Similarly, when r = 0.75 was assumed, the pooled effect was g = 0.011 (95% CI −0.186 to 0.208, *p* > 0.05), with low heterogeneity (I^2^ = 10.6%; τ^2^ = 0.011).

**Figure 4:**
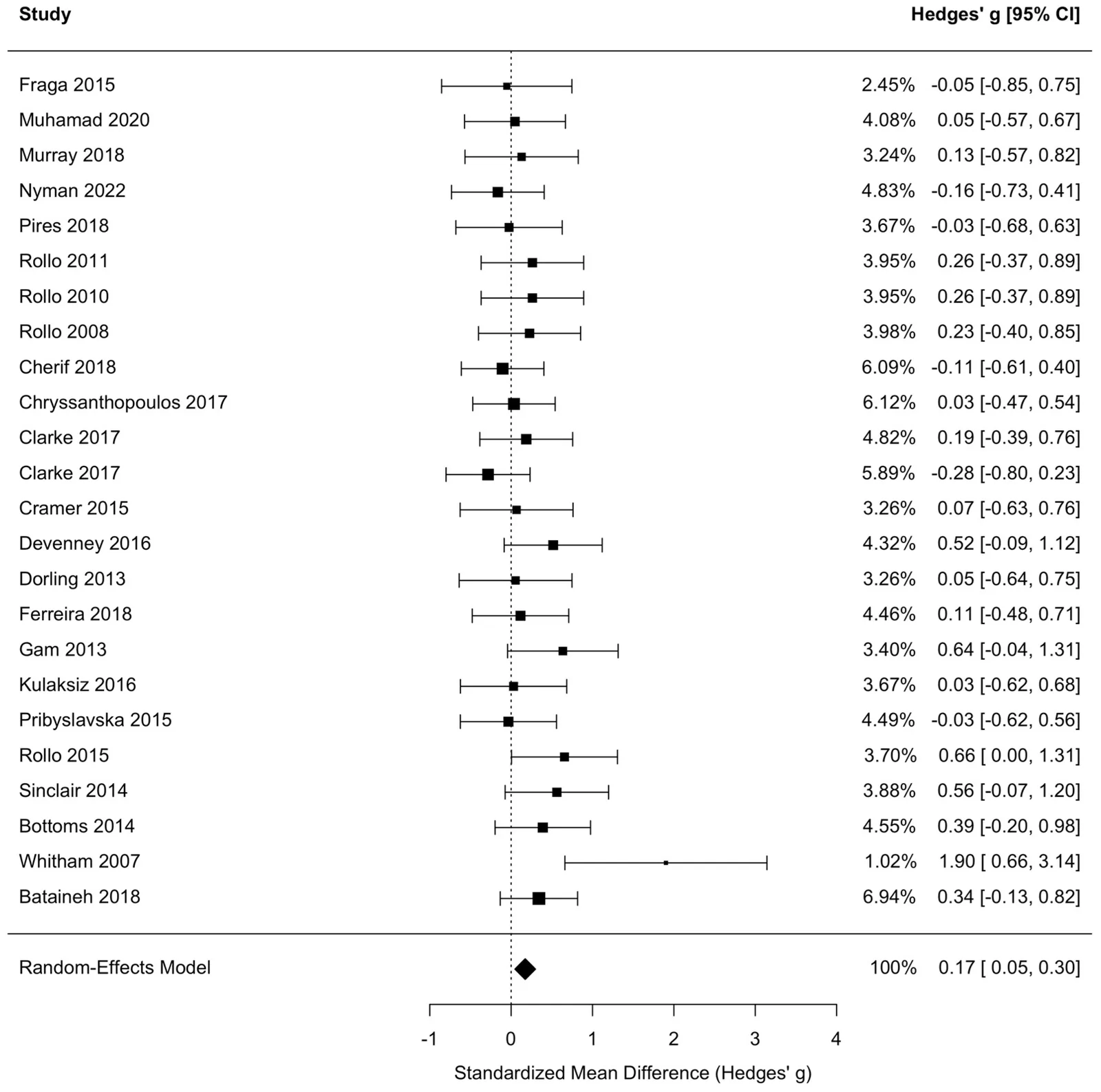
Forest plot for speed performance [27,28,29,30,46,58,61,63,65,66,67,68,69,71,75,76,79,80,81,83,85,86,87,89].

Regarding speed, our meta-analysis showed that CHO mouth rinse increased speed performance compared with the control condition (Hedges’ g = 0.17, 95% CI 0.05 to 0.30, *p* < 0.01, Figure 4). There was no evidence of between-study heterogeneity (I^2^ = 0%; τ^2^ ≈ 0), and Cochran’s Q test was non-significant (Q(23) = 22.73, *p* > 0.05). Sensitivity analyses showed that at r = 0.25, the pooled effect remained statistically significant (Hedges’ g = 0.149, 95% CI 0.024 to 0.273, *p* = 0.02; I^2^ = 0%). At r = 0.75, the effect remained statistically significant and increased slightly in magnitude (g = 0.231, 95% CI 0.083 to 0.380, *p* < 0.01), with low-to-moderate heterogeneity (I^2^ = 25.2%; τ^2^ = 0.034). A visual inspection of the forest plot identified Whitham et al. (2007) [30] as an influential study. Leave-one-out sensitivity analysis showed that the pooled effect was robust to the sequential exclusion of individual studies, with pooled Hedges’ g estimates ranging from approximately 0.15 to 0.20 and remaining statistically significant in all analyses. Although Whitham et al. (2007) [30] showed a comparatively large individual effect (Hedges’ g = 1.90), its exclusion did not alter the overall pooled estimate (Hedges’ g = 0.17, 95% CI 0.047 to 0.298, *p* < 0.01).

Subgroup analysis according to exercise type showed no evidence that the intervention effect differed among exercise modalities (QM(2) = 2.385, *p* > 0.05). A statistically significant effect was observed for cycling studies (k = 9; Hedges’ g = 0.276, 95% CI 0.062 to 0.489, *p* = 0.01; I^2^ = 0%), whereas the pooled effect for running studies did not reach statistical significance (k = 14; g = 0.141, 95% CI −0.020 to 0.302, *p* > 0.05; I^2^ = 0%). Hockey was represented by a single study (g = −0.164, 95% CI −0.733 to 0.406, *p* > 0.05). Similarly, the CHO mouth rinse type did not significantly moderate the intervention effect (QM(2) = 1.142, *p* > 0.05). The maltodextrin subgroup demonstrated a significant pooled effect (k = 15; Hedges’ g = 0.205, 95% CI 0.035 to 0.374, *p* = 0.02; I^2^ = 12.6%), whereas the dextrose subgroup did not (k = 8; g = 0.085, 95% CI −0.143 to 0.313, *p* > 0.05; I^2^ = 0%). Sucrose was represented by one study (g = 0.341, 95% CI −0.134 to 0.816, *p* > 0.05). Participant fitness level was also not a significant moderator (QM(1) = 1.409, *p* > 0.05). Among recreationally active participants, the pooled effect was statistically significant (k = 19; Hedges’ g = 0.209, 95% CI 0.070 to 0.347, *p* < 0.01; I^2^ = 0%), whereas no significant effect was observed among athletes (k = 5; g = 0.012, 95% CI −0.281 to 0.305, *p* > 0.05; I^2^ = 0%).

Subgroup analysis according to CHO mouth rinse solution concentration also showed no evidence of effect modification (QM(1) = 0.504, *p* > 0.05). Solutions with concentrations ≤ 8% produced a statistically significant pooled effect (k = 21; Hedges’ g = 0.154, 95% CI 0.019 to 0.290, *p* = 0.02; I^2^ = 0%), whereas concentrations > 8% did not (k = 3; g = 0.322, 95% CI −0.159 to 0.804, *p* > 0.05; I^2^ = 50.9%). Finally, Egger’s regression test indicated significant funnel-plot asymmetry (z = 2.458, *p* = 0.01, Figure S10), suggesting the presence of small-study effects. The limit estimate was −0.811 (95% CI −1.605 to −0.017).

**Figure 5:**
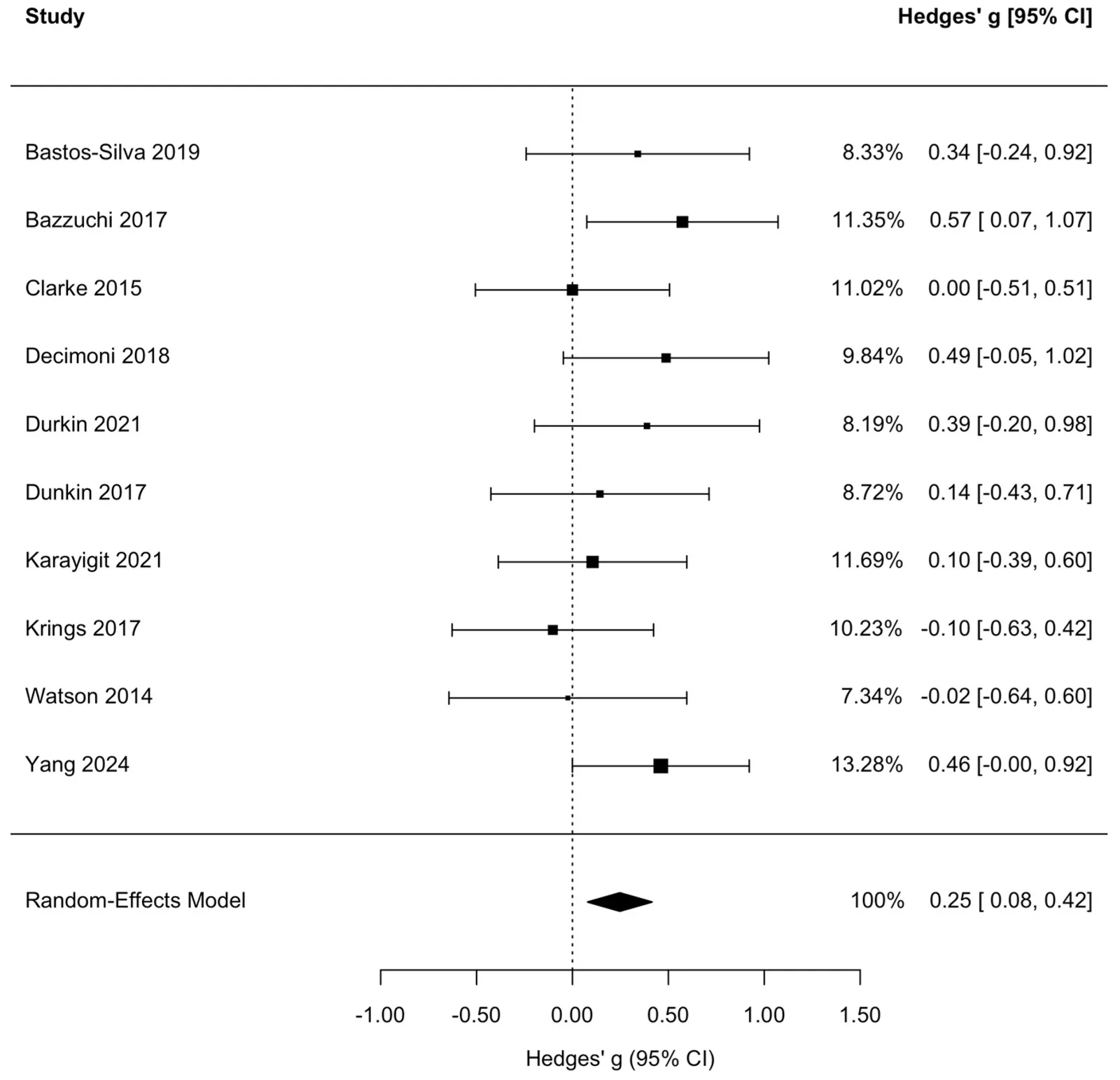
Forest plot for workload [22,23,24,25,36,91,92,93,94,95].

Furthermore, meta-analysis showed that CHO mouth rinse increased workload compared with the control condition (Hedges’ g = 0.25, 95% CI 0.08 to 0.41, *p* < 0.01, Figure 5). There was no evidence of between-study heterogeneity (I^2^ = 0%; τ^2^ = 0), and Cochran’s Q test was non-significant (Q(9) = 7.37, *p* > 0.05). Sensitivity analyses showed that at r = 0.25, the pooled effect remained statistically significant (Hedges’ g = 0.205, 95% CI 0.039 to 0.372, *p* = 0.02; I^2^ = 0%; τ^2^ = 0). At r = 0.75, the pooled effect remained significant and increased in magnitude (g = 0.333, 95% CI 0.117 to 0.548, *p* < 0.01), with moderate heterogeneity (I^2^ = 36.0%; τ^2^ = 0.043).

Subgroup analysis according to exercise type showed no statistically significant difference between cycling and resistance exercise (QM(1) = 2.909, *p* > 0.05). The resistance exercise subgroup demonstrated a statistically significant pooled effect (k = 8; Hedges’ g = 0.315, 95% CI 0.130 to 0.500, *p* < 0.01; I^2^ = 0%), whereas the cycling subgroup did not (k = 2; g = −0.070, 95% CI −0.470 to 0.331, *p* > 0.05; I^2^ = 0%). Similarly, the CHO mouth rinse type did not significantly moderate the intervention effect (QM(1) = 2.909, *p* > 0.05). The maltodextrin subgroup showed a significant positive pooled effect (k = 8; Hedges’ g = 0.315, 95% CI 0.130 to 0.500, *p* < 0.01; I^2^ = 0%), whereas the dextrose subgroup did not (k = 2; g = −0.070, 95% CI −0.470 to 0.331, *p* > 0.05; I^2^ = 0%).

Subgroup analysis according to CHO mouth rinse solution concentration also showed no statistically significant effect modification (QM(1) = 2.416, *p* > 0.05). Solutions with concentrations ≤ 8% produced a significant pooled effect (k = 7; Hedges’ g = 0.336, 95% CI 0.134 to 0.537, *p* < 0.01; I^2^ = 0%), whereas concentrations > 8% did not (k = 3; g = 0.047, 95% CI −0.257 to 0.350, *p* > 0.05; I^2^ = 0%). A subgroup analysis according to participant fitness level was not undertaken because only one study was available in the athlete subgroup. Finally, Egger’s regression test showed no evidence of funnel-plot asymmetry (z = −0.649, *p* > 0.05, Figure S11).

**Figure 6:**
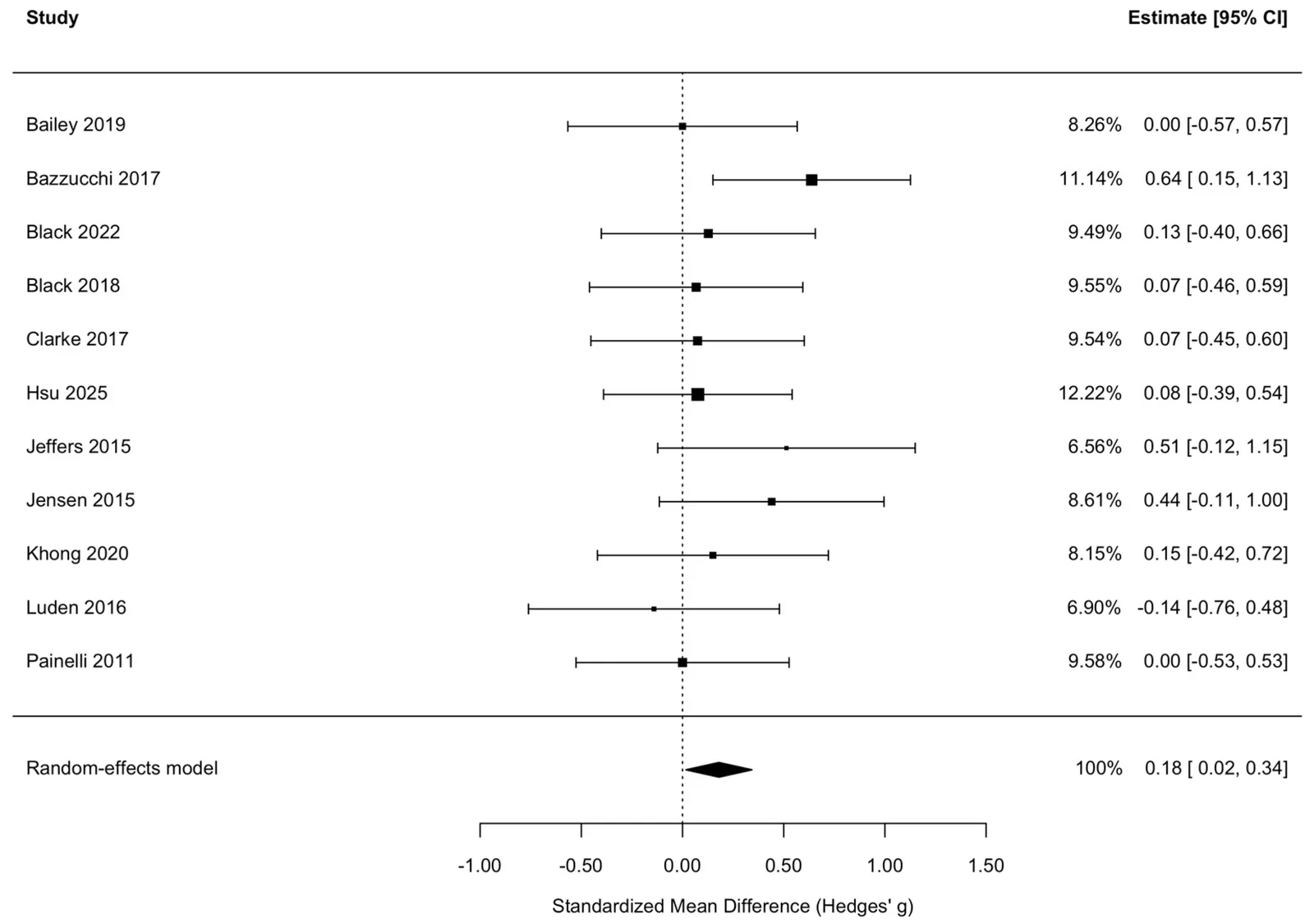
Forest plot for MVC [29,36,37,38,39,84,96,97,98,99,100].

Our meta-analysis also revealed that CHO mouth rinse increased MVC compared with the control condition (Hedges’ g = 0.18, 95% CI 0.02 to 0.34, *p* = 0.03, Figure 6). There was no evidence of between-study heterogeneity (I^2^ = 0%; τ^2^ = 0), and Cochran’s Q test was non-significant (Q(10) = 7.73, *p* > 0.05). Sensitivity analyses showed that at r = 0.25 the pooled effect was smaller and did not reach statistical significance (Hedges’ g = 0.15, 95% CI −0.01 to 0.31, *p* > 0.05; I^2^ = 0%; τ^2^ = 0). At r = 0.75, the effect remained statistically significant and was slightly larger (g = 0.24, 95% CI 0.05 to 0.43, *p* = 0.01), with low heterogeneity (I^2^ = 24.2%; τ^2^ = 0.025).

Subgroup analysis according to exercise type found no evidence that the effect differed between exercise modalities (QM(2) = 0.25, *p* > 0.05). A statistically significant effect was observed among resistance-exercise studies (k = 7; Hedges’ g = 0.20, 95% CI 0.003 to 0.40, *p* = 0.05; I^2^ = 0%), whereas the pooled effect for cycling studies was not significant (k = 3; g = 0.17, 95% CI −0.18 to 0.52, *p* > 0.05; I^2^ ≈ 0%). The single grip-test study showed an effect of g = 0.08 (95% CI −0.39 to 0.54, *p* > 0.05). Subgroup analysis according to CHO mouth rinse type similarly showed no significant difference between dextrose and maltodextrin (QM(1) = 0.38, *p* > 0.05). The maltodextrin subgroup demonstrated a small statistically significant pooled effect (k = 9; Hedges’ g = 0.20, 95% CI 0.02 to 0.38, *p* = 0.03; I^2^ = 0%), whereas the dextrose subgroup did not (k = 2; g = 0.07, 95% CI −0.32 to 0.46, *p* > 0.05; I^2^ = 0%).

Participant fitness level did not significantly moderate the pooled effect (QM(1) = 0.72, *p* > 0.05). Among athletes, the pooled effect was g = 0.30 (k = 3; 95% CI −0.02 to 0.61, *p* > 0.05; I^2^ = 0%), whereas among recreationally active participants it was g = 0.14 (k = 8; 95% CI −0.05 to 0.33, *p* > 0.05; I^2^ = 0%). CHO mouth rinse solution concentration subgroup analysis was not performed because all the included studies in this meta-analysis displayed solution concentrations of ≤8%. Finally, Egger’s regression test showed no evidence of funnel-plot asymmetry (z = −0.27, *p* > 0.05, Figure S12).

GRADE analysis outcomes and the final interpretation outcomes can be found in Table 1 and the detailed GRADE analysis can be found in Table S4.

## 4. Discussion

The aim of the current systematic review and meta-analysis was to examine the acute effects of CHO mouth rinse on exercise capacity and performance in humans at any health and fitness status, by synthesizing evidence from 90 controlled trials.

### 4.1. Completeness and Applicability of Evidence

Overall, our findings indicate that CHO mouth rinse produced trivial to small but statistically significant improvements in distance covered, performance time, speed, workload, and MVC, whereas no significant effects were observed for muscular endurance, power output, or VO_2_max. Importantly, the beneficial effects on distance covered, performance time, speed, and workload were generally robust to alternative assumptions (r = 0.25, r = 0.75) regarding within-participant correlations. This was not the case for MVC, given that one of the alternative assumptions (r = 0.25) did not reach statistical significance as the main assumption (r = 0.50). Overall, the findings support an ergogenic effect of CHO mouth rinsing, but this effect is trivial to small and outcome-specific. Generally, we found an acceptable number of CTs (>8) and participants (>100) included in each meta-analysis, indicating an adequate sample size to examine the particular research question [17,54].

Regarding distance covered, the overall effect size was trivial, while we detected no differences between exercise type, athletes and recreational sport participants or CHO mouth rinse type. Even though the increase in distance covered is little to no different, this potential improvement may have implications in endurance sports in elite athletes, where even minor performance gains can influence competitive outcomes. However, this was not confirmed in our meta-analysis, given that we found no differences in distance covered between athletes and recreational participants. This finding aligns closely with the overall effects reported by Hartley et al. (2022) [7] in their systematic review focused specifically on maltodextrin-based CHO mouth rinse and concluded that maltodextrin provides a small but consistent improvement in exercise performance. Performance time also improved following CHO mouth rinse, although the magnitude of the effect was trivial according to Cochrane criteria [17]. There is a possibility that, in competitive endurance sports, even such a trivial improvement may be practically meaningful. However, given the absence of a significant subgroup difference between athletes and recreational participants in our meta-analysis, the suggestion that athletes may possess greater pacing precision, thereby allowing subtle changes in central motivation to translate into measurable improvements in performance [101], should be interpreted with caution. Interestingly, our finding differs from Brietzke et al.’s (2019) [6] systematic review, which reported no significant improvement in cycling time-trial completion time. However, the magnitude of their pooled estimate (SMD = −0.13) was very similar to ours. Therefore, this discrepancy may relate more to statistical precision rather than to the estimated magnitude of effect. Finally, our subgroup analyses indicated no evidence of differences between exercise type, fitness level and CHO mouth rinse type as well as solution concentration.

The CHO mouth rinse produced a trivial but significant improvement in exercise speed. Speed in sports such as cycling is very important and even trivial changes may be practically meaningful, as relatively minor improvements can influence an athlete’s probability of competitive success [102]. We found however, no effect of CHO mouth rinse with regard to exercise type, fitness level or CHO mouth rinse type as well as solution concentrations. Similarly, we found improvement in MVC that was trivial but statistically significant, with no differences in subgroup analyses between exercise type, fitness level, or CHO mouth rinse type. The proposed mechanism for this finding is that CHO receptors located within the oral cavity appear to activate reward- and motor-related brain regions involved in exercise regulation [103]. This potentially activates the anterior cingulate cortex, orbitofrontal cortex and striatum independently of sweetness perception, suggesting a central neural mechanism that enhances motor drive and motivation rather than energy supply [3]. However, our results for MVC should be treated with caution, given that our sensitivity analysis for within-participants correlation showed less robust evidence than the other outcomes in the current meta-analysis.

Workload was also significantly increased following CHO mouth rinse, with subgroup analyses indicating no differences between exercise type or CHO mouth rinse type as well as solution concentration. Similar to MVC, the proposed mechanism for increased workload likely reflects enhanced central motor activation rather than improved muscle energetics [104]. Since workload represents the cumulative mechanical work completed during exercise, improvements may result from the ability to sustain higher contraction intensities or complete additional repetitions before fatigue. These findings may support the concept that oral CHO exposure predominantly influences central determinants of exercise performance; however, these influences cannot directly be supported by the current meta-analysis. In contrast to these positive findings, no significant effects were identified for muscular endurance, power output and VO_2_max. For muscular endurance and power output, this finding may depend primarily on neuromuscular and metabolic characteristics that may not be sufficiently influenced by transient activation of oral CHO receptors [4]. The absence of improvement in VO_2_max is not surprising because maximal oxygen uptake reflects the integrated function of the cardiovascular, respiratory and muscular systems and is unlikely to be altered by an acute sensory intervention [4].

### 4.2. Agreements and Disagreements with Previous Systematic Reviews

Our results agree with the systematic review by de Ataide-Silva et al. (2013) [5], which concluded that CHO mouth rinse exerts small but meaningful benefits on endurance performance while highlighting considerable methodological heterogeneity across studies. Likewise, our findings are generally consistent with the meta-analysis of Suen et al. (2026) [9], which reported no significant benefits in exercise performance by including different high-intensity intermittent exercise protocols, indicating that the magnitude of benefit varied according to exercise protocol. The present review showed no differences in exercise types across all outcomes.

Hartley et al.’s (2022) [7] systematic review focused specifically on maltodextrin-based CHO mouth rinses and concluded that maltodextrin provides a small but consistent improvement in exercise performance, particularly when using concentrations of approximately 6–6.5%. Our subgroup analyses did not support this finding, given that we found no effect differences in CHO mouth rinse type and solution across all outcomes. The systematic review by Oliveira-Silva et al. (2022) [8] demonstrated that CHO mouth rinse improves muscular endurance but not maximal strength. Our findings confirm these observations but further demonstrate that CHO mouth rinse produces a trivial yet significant increase in MVC.

Finally, Brietzke et al. (2019) [6] reported no significant effects of CHO mouth rinse on cycling power output or time-trial performance. Consistent with these findings, our meta-analysis demonstrated that CHO mouth rinse does not meaningfully influence power output. Although we observed a statistically significant improvement in performance time, the magnitude of the effect was trivial. Unlike the study by Brietzke et al., our analysis incorporated multiple endurance exercise modalities in addition to cycling, suggesting that even these small reductions in performance time may be practically relevant in competitive endurance events, where race outcomes are frequently determined by differences in only a few seconds or even milliseconds.

### 4.3. Strengths and Limitations

Several strengths distinguish the present systematic review from previous syntheses. To our knowledge, this represents the largest systematic review and meta-analysis examining CHO mouth rinse, incorporating 90 controlled trials across a broad spectrum of exercise modalities and participant characteristics. The extensive subgroup analyses enabled detailed exploration of exercise type, CHO type and concentration as well as fitness level as potential moderators of treatment effects. Furthermore, the incorporation of GRADE methodology provides transparent evaluation of evidence certainty, enhancing the clinical applicability of the findings.

Several limitations should also be acknowledged. Publication bias was detected for several outcomes, indicating that small-study effects cannot be excluded. Additionally, many subgroup analyses were based on relatively few studies, limiting statistical power and increasing the possibility of type II error. Differences in rinse duration, frequency of mouth rinsing, CHO exposure time and blinding procedures (single vs. double) were inconsistently reported across studies and may have contributed to variability. Finally, the predominance of young healthy adults and male participants—96.45% of the overall sample size—limits generalizability to older populations, females and clinical groups.

### 4.4. Deviation from the Published Protocol

We report the following deviations and clarifications from the initially published protocol, as documented in the updated protocol. First, we clarified the eligibility criteria regarding CHO consumption during the intervention. Second, we clarified the prespecified outcomes. Specifically, “time to complete a specific exercise” was revised to “exercise performance in (a) time and (b) distance” to more accurately reflect the outcomes assessed in the eligible studies. In addition, “muscular endurance” was included as a separate outcome alongside “power,” whereas the initial protocol specified only “power,” thereby allowing these distinct exercise outcomes to be appropriately differentiated. Third, we added “CHO mouth-rinse concentration” as a prespecified subgroup variable in the updated protocol; this subgroup analysis had not been specified in the initial protocol. Fourth, we expanded the description of the data synthesis strategy. In particular, RStudio (version 4.6.1 (24 June 2026); Posit Software, PBC, Boston, MA, USA) was used instead of IBM SPSS Statistics, version 31.0 (IBM Corp., Armonk, NY, USA) to enable the appropriate statistical handling of the eligible crossover-design studies. The updated approach incorporated methods for calculating effect sizes and their variances, as well as revised procedures for assessing heterogeneity and conducting subgroup and sensitivity analyses. In addition, the updated protocol provides detailed information on the methods used to calculate or impute SDs, as well as methods to calculate means and SDs from a figure. Finally, the updated protocol includes GRADE as an approach to evaluate the certainty of the evidence.

## 5. Conclusions

In conclusion, the present findings both confirm and refine previous meta-analytic evidence regarding CHO mouth rinsing. Earlier syntheses generally suggested a small ergogenic effect, with standardized estimates typically around 0.15–0.25. Our findings are highly consistent with this magnitude but no consistent evidence of effect modification by exercise type was found. CHO mouth rinse produced trivial improvements in distance covered, performance time and speed as well as small improvements in workload, with less robust evidence for MVC, while no effects were identified for muscular endurance, power output, or VO_2_max; evidence that mainly applies to male participants. Therefore, the available data suggest that CHO mouth rinsing can produce a small, outcome-dependent ergogenic effect, the practical importance of which is likely to vary according to the exercise context and competitive context. At the same time, the lack of consistent moderation by CHO type, solution concentration, exercise type, or fitness level indicates that there is currently insufficient evidence to prescribe a clearly superior mouth-rinse protocol or identify a population that consistently derives greater benefit. As such, future research should prioritize standardized CHO mouth rinse protocols, including consistent CHO concentrations, rinse durations and administration frequencies. Direct comparisons between maltodextrin, dextrose and other CHO formulations are needed to clarify whether molecular structure influences oral receptor activation. Additional studies should also investigate sex differences, older adults, clinical populations and sporting environments, where tactical and environmental factors may influence responsiveness.

## Figures and Tables

**Table 1:** Meta-analysis and GRADE analysis outcome.

Outcome	Relative Effect SMD (95% CI)	Magnitude of Effect *	Publication Bias (Egger Test)	Certainty of Evidence (GRADE)	Certainty of Evidence Comment	Final Interpretation #
Muscular endurance	0.49 (−0.31, 1.29)	Moderate	No (*p* > 0.05)	Moderate ⨁⨁⨁◯	Due to inconsistency	CHO mouth rinse does not affect muscular endurance
Distance covered	0.17 (0.03, 0.30)	Trivial	No (*p* > 0.05)	Moderate ⨁⨁⨁◯	Due to imprecision	CHO mouth rinse probably results in little increase to no difference in distance covered
Performance time	−0.15 (−0.25, −0.05)	Trivial	Yes (*p* < 0.01)	Moderate ⨁⨁⨁◯	Due to publication bias	CHO mouth rinse probably results in little reduction to no difference in performance time
Power output	0.008 (−0.13, 0.14)	Trivial	Yes (*p* < 0.01)	Low ⨁⨁◯◯	Due to publication bias and imprecision	CHO mouth rinse does not affect power output
VO_2_max	−0.003 (−0.18, 0.18)	Trivial	No (*p* > 0.05)	Moderate ⨁⨁⨁◯	Due to imprecision	CHO mouth rinse does not affect VO_2_max
Speed	0.17 (0.05, 0.30)	Trivial	Yes (*p* = 0.01)	Moderate ⨁⨁⨁◯	Due to publication bias	CHO mouth rinse probably results in little increase to no difference in speed
Workload	0.25 (0.08, 0.41)	Small	No (*p* > 0.05)	High ⨁⨁⨁⨁		CHO mouth rinse slightly increases workload
Maximum voluntary contraction	0.18 (0.02, 0.34)	Trivial	No (*p* > 0.05)	Moderate ⨁⨁⨁◯	Due to imprecision	CHO mouth rinse probably results in little increase to no difference in maximum volitional contraction

* Magnitude of effect is defined according to *Cochrane Handbook*, chapter 15.5.3.1 (<0.2 = Trivial effect; 0.2–0.5 = Small effect; 0.5–0.8 = Moderate effect; >0.8 = Large effect); Confidence interval = CI; Standardized mean differences = SMD; VO_2_max = maximum oxygen consumption. # Final interpretation is based on *Cochrane Handbook*, chapter 15, Table 15.6 [17].

## Data Availability

No new data were created or analyzed in this study. Data sharing is not applicable to this article.

## Supplementary Materials

The following supporting information can be downloaded at https://www.mdpi.com/article/10.3390/nu18183099/s1, Search algorithm; Table S1: Characteristics of included studies [105,106,107,108,109,110,111,112,113,114]; Table S2: Risk of bias; Figure S1: Risk of bias summary; Figure S2: Forest plot for muscular endurance; Figure S3: Forest plot for muscular endurance without Pereira et al. 2020 study; Figure S4: Funnel plot for distance covered; Figure S5: Funnel plot for performance time; Figure S6: Forest plot for power output; Figure S7: Funnel plot for power output; Figure S8: Forest plot for VO_2_max; Figure S9: Funnel plot for VO_2_max; Figure S10: Funnel plot for speed; Figure S11: Funnel plot for workload; Figure S12: Funnel plot for MVC; Table S3: PRISMA checklist; Table S4: GRADE analysis.

## Abbreviations

The following abbreviations are used in this manuscript:

PRISMA	Preferred Reporting Items for Systematic Reviews and Meta-Analyses
CHO	Carbohydrates
VO_2_max	Maximal oxygen consumption
PICOS	Population, intervention, comparator, outcome, study design
CT	Controlled trials
MVC	Maximum Voluntary Contraction
SMD	Standardized mean differences
SD	Standard deviation
CV	Coefficient of variation
GRADE	Grading of Recommendations Assessment, Development and Evaluation
