# Supplementary material for: Effects of Carbohydrate Mouth Rinse on Human Capacity and Performance—A Systematic Review and Meta-Analysis of 90 Controlled Trials
Source: Nutrients. 2026 Sep 21;18(18):3099. doi: 10.3390/nu18183099 (PMC13610015; doi:10.3390/nu18183099)
Supplement: Supplementary file 1 [file nutrients-18-03099-s001.zip › nutrients-4495202-supplementary.pdf]

SUPPLEMENT

Effects of carbohydrates mouth rinse on human capacity and performance. A systematic review and meta-analysis of 90 controlled trials

Constantine Zalagkitis, Anastasia Kraniotaki, Nikolaos Simitzis, Konstantinos Dallas, Athanasios Moustogiannis, Eirini Chatzinikita, Giorgos Paradisis, Roxane Tenta, Maria Maridaki, Aristeidis Veskoukis, George Metsios, Anastassios Philippou, Costas Chryssanthopoulos, Petros C. Dinas

Search word algorithm in PubMed

(("CHO"[Title/Abstract] OR "carbohydrate*"[Title/Abstract] OR "glucose"[Title/Abstract] OR "maltodextrin"[Title/Abstract] OR "dextrose"[Title/Abstract] OR "high glycaemic index carbohydrate*"[Title/Abstract] OR "high glycaemic index carbohydrate*"[Title/Abstract] OR "high carbohydrate*"[Title/Abstract] OR "high carbohydrate*"[Title/Abstract] OR "low glycaemic index carbohydrate*"[Title/Abstract] OR "low glycaemic index carbohydrate*"[Title/Abstract] OR "carbohydrate electrolyte*"[Title/Abstract] OR "carbohydrate electrolyte*"[Title/Abstract] OR "carbohydrate solution"[Title/Abstract] OR "low carbohydrate*"[Title/Abstract] OR "low carbohydrate*"[Title/Abstract] OR "cho solution"[Title/Abstract] OR "fructose"[Title/Abstract] OR "galactose"[Title/Abstract] OR "mannose"[Title/Abstract] OR "sucrose"[Title/Abstract] OR "dextrin"[Title/Abstract]) AND ("Mouth-Rinse"[Title/Abstract] OR "Mouth-Rinse"[Title/Abstract] OR "Mouth-Rinsing"[Title/Abstract] OR "Mouth-Rinsing"[Title/Abstract] OR "Mouth-wash"[Title/Abstract] OR "Mouth-wash"[Title/Abstract] OR "mouth-washing"[Title/Abstract] OR "mouth-washing"[Title/Abstract]) AND ("Exercise"[Title/Abstract] OR "Performance"[Title/Abstract] OR "endurance performance"[Title/Abstract] OR "exercise capacity"[Title/Abstract] OR "endurance exercise"[Title/Abstract] OR "Capacity"[Title/Abstract] OR "high intensity"[Title/Abstract] OR "intermittent exercise"[Title/Abstract] OR "Power"[Title/Abstract] OR "Strength"[Title/Abstract] OR "fitness"[Title/Abstract])) NOT ("animals"[MeSH Terms] NOT "humans"[MeSH Terms])

Search word algorithm in Ovid

1	CHO.ab,ti.	6072
2	carbohydrate*.ab,ti.	30632
3	glucose.ab,ti.	158326
4	maltodextrin.ab,ti.	586
5	dextrose.ab,ti.	4320
6	high-glycaemic index carbohydrate*.ab,ti.	6
7	high glycaemic index carbohydrate*.ab,ti.	6
8	high-carbohydrate*.ab,ti.	1552
9	high carbohydrate*.ab,ti.	1552
10	low-glycaemic index carbohydrate*.ab,ti.	5
11	low glycaemic index carbohydrate*.ab,ti.	5
12	carbohydrate-electrolyte*.ab,ti.	167
13	carbohydrate electrolyte*.ab,ti.	167
14	carbohydrate solution.ab,ti.	73

15 low-carbohydrate*.ab,ti. 1666
16 low carbohydrate*.ab,ti. 1666
17 CHO solution.ab,ti. 42
18 fructose.ab,ti. 6231
19 galactose.ab,ti. 4605
20 mannose.ab,ti. 4822
21 sucrose.ab,ti. 14173
22 dextrin.ab,ti. 343
23 1 or 2 or 3 or 4 or 5 or 6 or 7 or 8 or 9 or 10 or 11 or 12 or 13 or 14 or 15 or 16 or 17 or 18 or 19 or 20 or 21 or 22 212659
24 Mouth Rinse.ab,ti. 394
25 Mouth-Rinse.ab,ti. 394
26 Mouth Rinsing.ab,ti. 164
27 Mouth-Rinsing.ab,ti. 164
28 Mouth wash.ab,ti. 69
29 Mouth-wash.ab,ti. 69
30 Mouth washing.ab,ti. 13
31 mouth-washing.ab,ti. 13
32 24 or 25 or 26 or 27 or 28 or 29 or 30 or 31 594
33 Exercise.ab,ti. 178694
34 Performance.ab,ti. 409836
35 endurance performance.ab,ti. 1281
36 Exercise Capacity.ab,ti. 8600
37 Endurance exercise.ab,ti. 2725
38 capacity.ab,ti. 158597
39 High intensity.ab,ti. 15946
40 Intermittent exercise.ab,ti. 586
41 Power.ab,ti. 122444
42 Strength.ab,ti. 131465
43 fitness.ab,ti. 36106
44 33 or 34 or 35 or 36 or 37 or 38 or 39 or 40 or 41 or 42 or 43 924792
45 23 and 32 and 44 90
46 limit 45 to human 90

Search word algorithm in SportDiscus

XB ("CHO" OR "carbohydrate*" OR "glucose" OR "maltodextrin" OR "dextrose" OR "high glycaemic index carbohydrate*" OR "high glycaemic index carbohydrate*" OR "high carbohydrate*" OR "high carbohydrate*" OR "low glycaemic index carbohydrate*" OR "low glycaemic index carbohydrate*" OR "carbohydrate electrolyte*" OR "carbohydrate electrolyte*" OR "carbohydrate solution" OR "low carbohydrate*" OR "low carbohydrate*" OR "cho solution" OR "fructose" OR "galactose" OR "mannose" OR "sucrose" OR "dextrin") AND XB ("Mouth-Rinse" OR "Mouth-Rinse" OR "Mouth-Rinsing" OR "Mouth-Rinsing" OR "Mouth-wash" OR "Mouth-wash" OR "mouth-washing" OR "mouth-washing") AND XB ("Exercise" OR "Performance" OR "endurance performance" OR "exercise capacity" OR "endurance exercise"

OR "Capacity" OR "high intensity" OR "intermittent exercise" OR "Power" OR "Strength" OR "fitness")

Table S1: Characteristics of included studies [1-90]

Code	ID	Design-location	Participants	Physical activity/exercise intervention	Nutrition/diet status	Mouth Rinse intervention	Outcomes
1	Ali 2017	Design: RCT<br>Location: New Zealand	Moderately trained males recreationally cyclists and triathletes<br>(n=9)<br>(age 32.7 ±13.0 y, height 1.80 ± 0.05 m, body mass 72.7±7.3 kg)<br>Experience:5-20h/week interpersed with competitive events	**Duration:** ~2 weeks (2 experimental trials):<br>**Exercise protocol:** On the first day a 90-minute glycogen-reducing exercise protocol took place. The main trial started with a 5-minute warm-up at 40% of Wmax and then a ~1-hour cycling time trial at the participant's prescribed intensity<br><br>**Conditions**: air-conditioned laboratory (temperature 18.2–20,4 °C, humidity 45,2-50%).	fasting for 12 hours<br>The last meal was a low CHO meal	**Volume:**1.5 mL·kg−1 body mass<br>**Solutions:**<br>1) CHOR: 15% CHO<br>2)PLAR: 0% CHO<br>**Timing:** immediately before exercise and after every 12.5 % of exercise completed<br>**Rinsing duration**:8sec	**Time trial performance (mean ± SD)**<br>CHOR: 68.4 ± 3.9 min<br>PLAR: 68.3 ± 5.2 min<br>No significant differences between conditions (p = 0.21)<br>**Power output**<br>There was a decline in power output throughout the time trial for all conditions(main effect of time ,p<0,01)
2	Ali 2016	Design: RCT<br>Location: New Zealand	Moderately trained males recreationally cyclists and triathletes<br>(n=9)<br>(age 32.7 ±13.0 y, height 1.80 ± 0.05 m, body mass 72.7±7.3 kg)<br>Experience:5-20h/week interpersed with competitive events	**Duration:** ~2 weeks (2 experimental trials):<br>**Exercise protocol:**On the first day a 90-minute glycogen-reducing exercise protocol took place. The main trial started with a 5-minute warm-up at 40% of Wmax and then a ~1-hour cycling time trial at the participant's prescribed intensity<br><br>**Conditions**: air-conditioned laboratory (temperature 18.2–20,4 °C, humidity 45,2-50%).	fasting for 12 hours<br>The last meal was a low CHO meal	**Volume:**1.5 mL·kg−1 body mass<br>**Solutions:**<br>1) CHOR: 15% CHO<br>2)PLAR: 0% CHO<br>**Timing:** immediately before exercise and after every 12.5 % of exercise completed<br>**Rinsing duration**:8sec	**Performance time**<br>No statistical difference between trials (p = 0.21)<br>**Power output**<br>• Power output decreased over time in all conditions (main effect of time, p < 0.01)<br>• No treatment × time interaction (p = 0.41)<br>**HR**<br>Increased with duration of exercise (main effect of time p<0.01),but was not different between trials(p=0,79)
3	Ataide-Silva 2016	Design: RCT<br>Location: Australia	Healthy, physically active males (n=8)<br><br>(age 31.5 ± 7.3 years, height 172.9 ± 5.2 cm, weight 74.1 ± 11.7 kg, body fat 12.8 ± 4.5%)	**Duration:** The study included 6 experimental trials, each lasting approximately 2 hours. The overall study duration in weeks was not specified.<br>**Exercise Protocol:**	1)<u>FED</u> (dinner + breakfast with a normal carbohydrate content (~56% CHO)<br>2)<u>FAST</u> (12-h overnight fast, no breakfast)<br>3)<u>DEP</u>(evening exercise to deplete muscle glycogen (~50–	**Volume:** 25 mL<br>**Solutions:**<br>1) CHO mouth rinse: 6.4% maltodextrin<br>2) Placebo (PLA): water<br>**Timing:**<br><u>Time-based:</u> immediately before exercise and at 5, 15, 25	Values are presented as means ±SD<br>***<u>Constant Load Exercise</u>***<br>**Baseline (PLA)**<br>**FED** 76 ± 6 (HR) ,<br>1.26±0.10(VO2)<br>**FAST** 71±7(HR)<br>1.21±0.08(VO2)

				30-minute cycling bout at 90% of GET, followed by a 20-km cycling time trial<br>**Conditions:** Temperature 22.4 ± 1.7°C, 45.4 ± 7.4% relative humidity	70%), low-CHO dinner (~12% CHO), 12-h overnight fast)	min of exercise<br>Distance-based: immediately before exercise and at 5, 10, 15, and 18 km<br><br>**Rinsing duration:** 10 seconds	**DEP** 74±7(HR)<br>1.24±0.17(VO2)<br>**Post-intervention(PLA)**<br>**FED** 138 ± 12(HR)<br>2.07±0.26(VO2)<br>**FAST** 135±16(HR)<br>2.03±0.26(VO2)<br>**DEP** 145±12(HR)<br>2.19±0.36(VO2)<br><br>**Baseline (CHO)**<br>**FED** 73 ±8(HR)<br>1.19±0.20(VO2)<br>**FAST** 72 ±5(HR)<br>1.24±0.10(VO2)<br>**DEP** 74±4(HR)<br>1.28±0.14(VO2)<br>**Post-intervention(CHO)**<br>**FED** 136 ±12 (HR)<br>2.06±0.20(VO2)<br>**FAST** 139±13(HR)<br>2.08±0.20(VO2)<br>**DEP** 146±11(HR)<br>2.22±0.43(VO2)<br>There was a main effect of pre-exercise CHO availability for heart rate ($p = 0.005$). The mean heart rate was higher in DEP than in both FED and FAST ($p = 0.017$ and $p = 0.001$, respectively), but there was no difference between FED and FAST ($p = 0.927$).<br>***20-km Time Trial***<br>**Baseline(PLA)**<br>**FED** 95±6(HR),<br>1.77±0.30(VO2)<br>**FAST** 96±10(HR)<br>1.77±0.34(VO2)<br>**DEP** 95±11(HR)<br>1.55±0.17(VO2)<br>**Post intervention(PLA)**

							**FED** 160±17(HR)<br>2.33±0.31(VO2)<br>**FAST** 147±23(HR)<br>2.07±0.41(VO2)<br>**DEP** 150±23(HR)<br>2.12±0.47(VO2)<br>**Baseline(CHO)**<br>**FED** 92±10(HR)<br>1.85±0.39(VO2)<br>**FAST** 93±12(HR)<br>1.69±0.32(VO2)<br>**DEP** 94±4(HR)<br>1.61±0.15(VO2)<br>**Post intervention(CHO)**<br>**FED** 154±20(HR)<br>2.36±0.38(VO2)<br>**FAST** 148±21(HR)<br>2.15±0.32(VO2)<br>**DEP** 144±22(HR)<br>2.00±0.38(VO2)<br>**Post / performance time:**<br>**DEP:** 46.34 ± 1.74<br>**FAST:** 42.43 ± 1.43 , slower than DEP(p = 0.044)<br>**FED:** 40.77 ± 1.50 , slower than DEP(p = 0.007)<br>**FAST vs FED:** p = 0.058<br>**CHO mouth rinse effect (post):**<br>**CHO rinse:** 42.47 ± 1.39<br>**Placebo (PLA):** 43.90 ± 1.51,CHO faster (p = 0.009)<br>**Simple effects:**<br>**DEP**: CHO faster than PLA, p = 0.019 → **very likely beneficial**<br>**FAST:** possibly beneficial<br>**FED:** negligible/trivial<br>**Power Output**<br>• CHO>PLA p = 0.027<br>• Higher in FED than in the FAST and DEP(no

							difference between the FAST and DEP
4	Bailey 2019	Design: RCT<br>Location: USA	Healthy moderately to highly physically active college-aged participants (n=10)<br>(<u>female</u> n=5,age=24±1 years; height=165±4 cm; body mass=61±3 kg; BMI=22.6±0.8)<br>(<u>male</u> n=5, (age=26±1 years; height=181±3 cm; body mass=85±5 kg; BMI=26.0±1.9)	**Duration**: 3 weeks(4 experimental trials)<br><br>**Exercise protocol:**<br>Each session included 3 MVICs followed by 10 quadriceps contractions at 50% of maximal force with 30 seconds rest between contractions. This protocol was performed before and after the mouth rinse condition.	Fasting 4 hours prior to the trial	**Volume**:25ml<br>**Solutions:**<br>1)PLA<br>2) GLU:6,4% glucose<br>3) MAL:6,4% maltose<br>4) MDX:6,4% maltodextrin<br><br>**Timing:** after the initial exercise protocol and a 10-minute rest period, with participants rinsing before immediately performing the next set of contractions.<br><br>**Rinsing duration**:20sec	**Torque generated during MVIC (M±SE)**<br><u>BASELINE(PRE)</u><br>PLAC 192±19 Nm<br>GLU 178±15 Nm<br>MAL 180±15 Nm<br>MDX 175±16 Nm<br><u>POST INTERVENTION (IMMEDIATELY AFTER)</u><br>PLAC 198±19 Nm<br>GLU 183±14 Nm<br>MAL 190±14 Nm<br>MDX 188±17 Nm<br>**EMG activity (M±SE) in the rectus femoris during 50% contraction and TMS**<br><u>BASELINE(PRE)</u><br>PLAC 6238±506 V<br>GLU 5682±615 V<br>MAL 5095±736 V<br>MDX 5028±510 V<br><br><u>POST INTERVENTION (IMMEDIATELY AFTER)</u><br>PLAC 6318±587μV<br>GLU 5613±693 μV<br>MAL 5207±718 μV<br>MDX 5384±541 μV
5	Baltazar-Martins 2019	Design: RCT<br>Location: USA	Well trained male cyclists (n=16)<br>(age = 37.6 ± 3.5 years; body mass = 76.9 ± 7.9kg)<br><br>**Experience**:5 years of cycling, with >5 competitions per year	**Duration**: about 2 weeks<br><br>Participants completed a 25.3-km simulated cycling time trial on a 3D cycling simulator with automatically adjusted resistance according to terrain slope. Prior to the trial, participants performed a 10-minute warm-up on their own bike. They were instructed to complete the course as fast as	Pre competition meal,3h before containing 3g/kg of body mass CHO	**Volume:25ml**<br>**Solutions:**<br>1) CHO(6.4% carbohydrates mix with sucrose, glucose, fructose)<br>2) PLA(no CHO)<br><br>**Duration:**5 sec<br>**Timing:**every 12,5% of the total cycling distance was completed	**Time to complete the 25.3-km trial(mean±SD)**<br>Completion time was reduced by 2.5 ± 5.1% compared with placebo (P = 0.04)<br>**Mean cycling power output (mean±SD)**<br>PlA 222 ± 51 W<br>CHO: 231 ± 46 W<br>Increase of 3.7 ± 9.4% with CHO (P = 0.04).

				possible, modulating power output through cadence and gear changes, as in real cycling conditions. The course included flat sections, climbs (>4% slope), and a final all-out sprint during the last 500 m.<br><br>**Conditions:**21.3 ± 0.3◦C air temperature and 50 ± 10% relative humidity			**Power output during climbing sections (>4% slope)(mean±SD)**<br>PLA: 238 ± 46 W<br>CHO: 248 ± 47 W<br>Increase of 4.2 ± 3.1% (P = 0.03).<br>**Power output during non-hill sections(mean±SD)**<br>PLA: 219 ± 52 W<br>CHO: 227 ± 45 W<br>Significant improvement with CHO (P = 0.05).<br>**Peak sprint power output(mean±SD)**<br>PLA 699 ± 234 W<br>CHO: 731 ± 272 W<br>No significant difference between conditions (P = 0.46).
6	Bastos-Silva 2016	Design: RCT<br>Location: Brazil	Physically active healthy men (n=13)<br>(age = 23.1±2.6 years; body mass = 74.7± 10.9 kg; height = 175± 6 cm; body fat = 14.2% ±6.8%)	**Duration**: About 2 weeks,5 trials with a minimum of 72 and maximum of 96 h intervals<br><br>**Exercise protocol:**<br>After 4 min of warm-up at 30W was conducted a high-intensity exercise at 110% of PPO **or** moderate-intensity exercise at 80% of respiratory compensation point	2 hours before they had their last meal	**Volume:** 25 mL<br><br>**Solutions:**<br>1) CHO mouth rinse: 6.4% maltodextrin<br>2) Placebo (PLA): no CHO<br><br>**Timing:**<br>Moderate-intensity exercise: immediately before exercise and every 15 minutes until exhaustion<br>High-intensity exercise: only immediately before exercise<br><br>**Rinsing duration:** 10 sec	**Time to Exhaustion (TE)(mean±SD)**<br>High-intensity exercise (110% PPO)<br>CHO: 177.3 ± 42.2 s<br>PLA: 163.0 ± 26.7 s<br>!No significant difference (p = 0.10)<br>Moderate-intensity exercise (80% RCP)<br>CHO: 76.6 ± 19.7 min<br>PLA: 65.4 ± 15.2 min<br>!CHO significantly increased TE by ~14% (p = 0.01)<br>**EMG Activity (Muscle Activation) (mean±SD)**<br>High-intensity exercise: No effect of CHO on RF (rectus femoris) or VL (vastus lateralis) EMG activity.<br>Moderate-intensity exercise: VL EMG was higher at 30 min and at exhaustion with CHO vs PLA. And RF EMG showed no effect from CHO

7	Bastos-Silva 2019	Design: RCT<br>Location: Brazil	Recreational resistance trained males<br><br>(age 22.08 ± 1.88 years; height 172.75 ± 6.20 cm; weight 76.65 ± 14.07 kg<br><br>**Experience:** 2 years	**Duration:** about 1,5 week(3 experimental trials)<br><br>**Exercise protocol:**<br>Muscular endurance (ME) was measured for each exercise (LP, leg press and BP, bench press).Participants performed repetitions at 80% of their 1-RM until muscular fatigue, and the maximum number of repetitions was recorded. Each repetition was performed with 2 seconds concentric + 2 seconds eccentric.<br><br>**Conditions:**22º ± 1º C	2 hour before they had their last meal	**Volume:**25ml<br>**Solutions:**<br>1)CHO :6.4% maltodextrin<br>2)Placebo (PLA) trial: No CHO<br>3)Control(CONT) trial: No mouth rinse.<br><br>**Duration:**10sec<br>**Timing:** immediately before the ME test	**Leg Press (LP)(mean±SD)**<br>Number of repetitions: No significant difference between CHO, PLA, or CONT (p = 0.68)<br>CHO: 13.5 ± 4.8<br>PLA: 11.5 ± 4.4<br>CONT: 12.4 ± 4.4<br>Total Load Volume (TLV)**:** No significant difference (p = 0.99)<br>CHO: 2006.7 ± 825.2 kg<br>PLA: 1712.5 ± 772.9 kg<br>CONT: 1817.1 ± 672.6 kg<br>**Bench Press (BP)(mean±SD)**<br>Number of repetitions:<br>CHO > CONT (p = 0.002)<br>CHO: 8.2 ± 1.6<br>PLA: 7.1 ± 2.4<br>CONT: 6.8 ± 1.8<br>Total Load Volume (TLV)**:**<br>CHO > CONT (p = 0.035)<br>CHO: 557.1 ± 155.4 kg<br>PLA: 495.9 ± 206.1 kg<br>CONT: 476.1 ± 175.3 kg
8	Bataineh 2018	Design: RCT<br>Location: USA	Sub-elite male runners(n=18)<br>(Age: 21±2 years, Weight: 68.1±5.7 kg)<br>**Experience:** aerobic physical activities 5-6 times a week(>90min/day)	**Duration:** about 2 weeks (3 experimental trials)<br><br>**Exercise protocol:**<br><br>Participants completed a 15–20 min warm-up consisting of light running and stretching. They then performed a modified Conconi test on a Life Fitness 95 T treadmill to determine peak oxygen uptake (VO_2max), maximal heart rate (HRmax), peak treadmill speed (VPeak), and running time to exhaustion. The test started at 8 km/h, with the speed increasing by 1 km/h every 400 m until volitional exhaustion.	Ramadan fasting period at least 12 h into fasting before the trial	**Volume:** 25ml<br><br>**Solutions:**<br>1) CHO: 7.5% sucrose solution<br>2) PLA: 7.5 g saccharine in 1lt water)<br>3) CON: no rinse.<br><br>**Timing:**5 min before exercise<br>**Rinsing duration:**10sec	**VO_2 (ml/kg/min)**<br>Running speed 8 km/h<br>CON: 15.6 ± 1.5<br>PLA: 15.5 ± 1.7<br>CHO: 15.3 ± 1.9<br>No significant difference between conditions (p = 0.345).<br>Running speed 12 km/h<br>CON: 35.7 ± 3.9<br>PLA: 35.7 ± 3.8<br>CHO: 35.5 ± 2.8<br>No significant difference between conditions (p = 0.857).<br>Running speed 16 km/h<br>CON: 49.4 ± 3.9<br>PLA: 48.6 ± 4.0<br>CHO: 47.5 ± 4.4<br>A trend toward lower VO_2 with

				**Conditions**:20º C temperature and 30% relative humidity)<br><br>**Conditions**: 20oC room temperature and 25-30% relative humidity			CHO was observed, but without statistical significance ($p = 0.065$).<br>**VO_2 (%VO_2max)**<br>Running speed 8 km/h<br>CON: 28.3 ± 3.3<br>PLA: 28.2 ± 3.4<br>CHO: 27.7 ± 3.8<br>No significant difference between conditions ($p = 0.346$).<br>**Running speed 12 km/h**<br>CON: 64.7 ± 5.8<br>PLA: 64.5 ± 6.5<br>CHO: 64.4 ± 4.7<br>No significant difference between conditions ($p = 0.875$).<br>**Running speed 16 km/h**<br>CON: 88.7 ± 5.9<br>PLA: 87.9 ± 6.6<br>CHO: 85.9 ± 7.1<br>No significant difference between conditions ($p = 0.076$).<br>**Heart Rate (HR)**<br>Baseline(8 km/h)<br>CON: 124.3 ± 6.2<br>PLA: 123.6 ± 5.1<br>CHO: 123.3 ± 5.3<br>No significant difference between conditions ($p = 0.682$).<br>Running speed 12 km/h<br>CON: 163.2 ± 13.3<br>PLA: 160.2 ± 10.4<br>CHO: 159.8 ± 11.3<br>No significant difference between conditions ($p = 0.176$).<br>Running speed 16 km/h<br>CON: 186.2 ± 6.1<br>PLA: 185.6 ± 5.1<br>CHO: 183.4 ± 5.9<br>No significant difference between conditions ($p = 0.147$).<br>**Time to exhaustion**

							CHO 1282.0±121.3s (Significantly higher than PLA and CON)<br>PLA 1258.1 ± 113.4s (Not different from CON)<br>CON 1228.7 ± 98.5s
9	Bavaresco Gambassi 2018	Design: RCT<br>Location: Brazil	Healthy, physically active, non-cyclist men (n = 21) (age 22.4 ± 2.7 years, height 176.7 ± 6.0 cm, body fat 12.1 ± 3.7%,BMI 23.9 ± 2.4 kg·m^{-2})<br>**Experience:** some of them engaged in physical activity at least 3d/week for 60 minutes	**Duration:** 3 weeks, with 1 trial per week.<br>**Exercise Protocol:** Each session began with a 5-minute warm-up at 25 W and 22 km·h^{-1} on a cycle ergometer. The test was then performed continuously, without rest intervals, at an intensity corresponding to each participant's individual anaerobic threshold (AT), until exhaustion.	Fasting 3 hours before each test session.	**Volume:** 25 mL at 4 °C<br>**Solutions:**<br>1)CHO: 6.4% maltodextrin<br>2)PLA: CHO-free<br>3)CON: no solution<br><br>**Timing:** at baseline and every 3 minutes during the exercise session<br>**Rinsing duration:**10 sec	**Heart Rate (HR)**<br>no significant differences, *p > 0.05*, across all three conditions both at the beginning and at the end of the exercise.<br>Baseline (mean ± SD)<br>CON: 76.1 ± 8.8 bpm<br>PLA: 74.0 ± 9.4 bpm<br>CHO: 74.1 ± 8.8 bpm<br>Post intervention(mean ± SD):<br>CON: 164.0 ± 12.3 bpm<br>PLA: 167.2 ± 11.8 bpm<br>CHO: 168.6 ± 13.0 bpm<br>**Time to Volitional Exhaustion**<br>significantly different between conditions (*p < 0.0001*).<br>**Time to exhaustion (mean ± SD)**<br>CON: 43.0 ± 27.5 minutes<br>PLA: 57.4 ± 30.6 minutes<br>CHO: 70.9 ± 30.3 minutes<br>CHO condition cycled about 13 minutes longer than in the PLA condition (*p < 0.05*).
10	Bazzucchi 2017	Design: RCT<br>Location: Italy	Moderately active men(n=18) (age 26.1 ± 5.0 yrs; body mass 70.6 ± 7.9 kg; height 1.79 ± 0.07 m)	**Duration:**12 days, with 1 trial every 72 hours<br>**Exercise protocol:**<br>5 sets of 30 maximal isokinetic elbow flexions at 180°/s (Isokinetic Fatiguing Task) with a duration about 5 min 15 s (including rests)	8-hour overnight fasting before each session<br><br>Standardized evening meal prior to each test	**Volume:**25ml<br>**Solutions:**<br>1) W (Water)<br>2)PLA (Placebo): (saccharin + aspartame)<br>3)MAL (Maltodextrin): 6.4% w/v maltodextrin<br>4)GLU (Glucose): 7.1% glucose<br>5)CON (Control): No mouth rinse	**Isokinetic Fatiguing Task (IFT)**<br>**Total Work :**<br>CON: 3729 ± 142<br>W: 3888 ± 110<br>PLA: 3853 ± 179<br>MAL: 4249 ± 184<br>GLU: 4316 ± 176<br>**End of IFT (5th set, % MVICPRE):**<br>**Torque:**

						**Timing**: before and during the isokinetic fatiguing task<br>**Rinsing duration:** 10 sec	CON: 71.7 ± 1.6 %<br>PLA: 69.7 ± 1.6 %<br>GLU: 64.1 ± 1.9 %<br>**MFCV:**<br>CON: 24.3 ± 2.3 %<br>PLA: 23.0 ± 2.3 %<br>GLU: 16.0 ± 3.0 %<br>**<u>MVICPOST (after IFT)</u>**<br>**Torque (% MVICPRE)**:<br>CON and PLA < MAL and GLU<br>**MFCV (% MVICPRE)**:<br>CON and PLA < GLU<br>*Mean Fiber Conduction Velocity (MFCV)
11	Beaven 2013	Design: RCT<br>Location: UK	Recreationally trained males(n=12)<br>(age, 32 ± 7.5 years; height, 175 ±7.3cm;mass,82.2±7.16kg	**Duration:**<br>~4 days (2 experimental trials)<br>**<u>Exercise protocol:</u>**<br>Participants performed repeated 6-second maximal cycling sprints on a calibrated Monark cycle ergometer. The protocol began with a 5-minute low-intensity warm-up, followed by five 6-second maximal sprint efforts. Resistance equivalent to 10% of body mass was applied to the front wheel, and each sprint was separated by 24 seconds of unloaded pedaling for recovery.	2 hour fasting prior to the session	**Volume:**?<br>**Solutions:**<br>1)CHO mouth rinse (6% w/v glucose solution)<br>2)PLA mouth rinse (noncaloric saccharin solution<br>**Timing:** During each 24-s recovery period<br>**Rinsing duration**: 5sec<br>.	**<u>Sprint 1:</u>**<br>**Peak power:**<br>CHO 22.1 ± 19.5 W ,<br>p = 0.067 ,it enhanced<br>**Mean power:**<br>CHO : +39.1 ± 26.9 W<br>p = 0.021 , significant improvement<br>**<u>Sprint 5:</u>**<br>**Peak power:**<br>CHO : −37.3 ± 30.2 W<br>p = 0.048 ,decreased in comparison to the PLA<br>**Mean power:**<br>CHO: −39.6 ± 27.4 W<br>p = 0.069, decreased in comparison to the PLA<br>**<u>Maximal heart rate</u>**<br>No difference<br>PLA: 158 ± 10 bpm<br>CHO: 162 ± 15 bpm
12	Beelen 2009	Design: RCT<br>Location: Netherlands	Endurance-trained male cyclists(n=14)	**Duration** :2 weeks(2 experimental trials)<br>**Conditions**:21,6±0,1°C,humidity 46%±1%	Standardized dinner and snacks the evening before	**Volume:**25ml<br>**Solutions:**<br>1) <u>CHO</u>:6.4% maltodextrin solution	<u>Data analyzed as M±SEM</u><br>**Performance Time**<br>CHO: 68.14 ± 1.14 min

			(age 24 ± 1 year, body weight 72.6 ± 2.4 kg, height 1.85 ± 0.02 m, BMI 21.1 ± 0.4 kg/m2)<br>**Experience: c**ompetitive cyclists ,exercised at least 2times/week and more than 100km/week	**Exercise protocol**<br>Performed on a cycle ergometer in linear mode.<br>10min warm up at 40% Wmax and then<br>Self-paced cycling time-trial to complete a fixed workload (1,053 ± 48 kJ) at 75% Wmax	Standardized breakfast <u>2 hour before the exercise</u> which provided 2.36 ± 0.04 g/kg of carbohydrates.	2)<u>PLA:</u> water<br><br>**Timing**<u>:</u> At the start and after every 12.5% of the time trial completed<br>**Rinsing Duration:** 5sec	PLA: 67.52 ± 1.00 min<br>No significant difference (p = 0.57)<br>**Average Power Output**<br>CHO: 265 ± 5 W<br>PLA: 266 ± 5 W<br>No significant difference (p = 0.58)<br>**Power Output over Time**<br>No treatment-by-time interaction (p = 0.44)<br>**Heart Rate**<br><u>Average</u><br>CHO 169±2beats/min<br>PLA 168±2beats/min<br><u>Post-intervention</u><br>CHO 180 ± 3 beats/min<br>PLA178 ± 3 beats/min
13	Black 2022	Design: RCT<br>Location: USA	Recreationally active men (n=12)<br>(age 23± 2 years; height 184 ± 7 cm;body mass 82 ±14kg; mean ± SD)<br>**Experience**: physically active, but not engaged in a structured training program	**Duration**: about 2 weeks (4 experimental trials)<br><br>**Exercise protocol:**<br>Isometric knee extensor contractions at 20% or 80% MVC held to volitional exhaustion (torque drop ≥10% for >5 s)	a light, mixed (50% carbohydrate, 30% fat, and 20% protein) meal 2 hours before<br><br>No caffeine for 12 hours before	**Volume:**25ml<br>**Solutions:**<br>1)CHO (8%maltodextrin and 0.2% sucralose artificial sweetener)<br>2) PLA (0.2% sucralose)<br><br>**Rinsing duration**: 20sec<br>**Timing**: immediately after exhaustion	<u>**BASELINE**</u><br>**Twitch torque (N·m)**<br>20% PLA: 62.0 ± 12.0<br>20% CHO: 62.7 ± 12.9<br>80% PLA: 61.9 ± 14.4<br>80% CHO: 61.8 ± 11.1<br>**Rate of torque rise (N·m·s⁻¹)**<br>20% PLA: 560.7 ± 113.2<br>20% CHO: 566.5 ± 128.4<br>80% PLA: 572.0 ± 145.9<br>80% CHO: 602.9 ± 148.3<br>**Rate of torque decline (N·m·s⁻¹)**<br>20% PLA: 448.8 ± 107.1<br>20% CHO: 449.8 ± 105.2<br>80% PLA: 457.8 ± 145.7<br>80% CHO: 457.8 ± 121.6<br><u>**IMMEDIATELY AFTER**</u><br>**Twitch torque (N·m)**<br>20% PLA: 56.7 ± 12.1<br>20% CHO: 54.1 ± 14.9<br>80% PLA: 57.4 ± 14.5<br>80% CHO: 54.7 ± 11.7

							**Rate of torque rise (N·m·s⁻¹)**<br>20% PLA: 542.5 ± 105.3<br>20% CHO: 556.2 ± 173.7<br>80% PLA: 503.6 ± 123.7<br>80% CHO: 509.3 ± 139.3<br>**Rate of torque decline (N·m·s⁻¹)**<br>20% PLA: 370.9 ± 116.4<br>20% CHO: 337.3 ± 90.6<br>80% PLA: 302.2 ± 76.9<br>80% CHO: 278.8 ± 62.9
14	Black 2018	Design: RCT<br>Location: USA	Recreationally active adults(n=13)<br>(6 men, 7 women; mean ± SD age 27.0 ± 3.0 years, height 168.2 ±12.2 cm, and body mass 76.9± 15.0 kg)	**Duration**: about 1 week (2 experimental trials)<br>**Exercise protocol:**<br>single prolonged isometric effort at submaximal intensity (50% MVC) until exhaustion	light, mixed (50% carbohydrate, 30% fat, and 20% protein) meal 2 hours before<br><br>No caffeine for 12 hours before	**Volume:**25 ml<br>**Solutions:**<br>1)CHO :8% maltodextrin and 0.2% sucralose)<br>2)PLA:.2% sucralose<br><br>**Rinsing duration**:20 sec<br>**Timing**: immediately after cessation of exercise	**Time to exhaustion**<br>CHO 96 ± 31sec<br>PLA 101 ± 44 sec<br>They did not differ(p=0.38)<br>**Twitch torque (N$m)**<br>Baseline<br>CHO 74.0 ±22.9<br>PLA 72.8 ± 25.3<br>Post-fatigue<br>CHO 61.5 ± 18.3<br>PLA 60.2±19.9<br>**MVC**<br>Post-fatigue<br>CHO 82.8 ± 7.3%,similar to PLA (p=0.18)<br>PLA 81.3 ± 9.9%
15	Bowling 2025	Design: RCT<br>Location: USA	NCAA Division I female soccer players (n=13)<br>(age 20.3 ± 1.5 years, height 170.3 ± 5.9 cm, body mass 64.8 ± 5.0 kg, body fat % 23.3 ± 3.6)	**Duration:** 2 weeks(2 experimental trials)<br><br>**Exercise protocol:**5-minute warm-up. Then 1 min rest.30-minute high-intensity, soccer-specific practice, which included scrimmage bouts to simulate match conditions. Immediately following the practice, participants had a 1-minute rest period. Then participants performed soccer-specific performance tasks.	For dinner they had one sandwich, a single serving of chips, a cookie, and water ad libitum with a nutritional value of 930 – 1080 kcal<br><br>500 mL water upon waking up on the morning	**Volume**:25ml<br>**Solutions:**<br>1)CHO(Powerade Zero with added maltodextrin (60 g per 1000 mL).<br>2)PLA(the same drink without CHO)<br>3)CON(no mouth rinse)<br><br>**Rinsing duration**:10-15sec<br>**Timing:** immediately after the practice during the 1 min rest	**Agility**<br>CON 10.9 ± 0.4<br>PLA 11.2 ± 0.4<br>CHO 11.1 ± 0.4<br>**Juggling (reps)**<br>CON 88.2 ± 20.3<br>PLA 88.3 ± 20.0<br>CHO 88.4 ± 18.4<br>**Shots made (right)**<br>CON 2.2 ± 1.5<br>PLA 1.7 ± 1.2<br>CHO 2.4 ± 1.4<br>**Shots made (left)**<br>CON 2.9 ± 1.1

							PLA 2.6 ± 1.3<br>CHO 2.7 ± 1.0<br>**Passes made (right)**<br>CON 3.2 ± 0.8<br>PLA 3.5 ± 0.8<br>CHO 3.3 ± 1.1<br>**Passes made (left)**<br>CON 2.9 ± 1.0<br>PLA 3.3 ± 1.3<br>CHO 3.0 ± 1.0<br>**HR(avg) (bpm)**<br>CON: 129.4 ± 13.3<br>PLA: 127.5 ± 11.8<br>CHO: 124.7 ± 11.8<br>**HRmax (bpm)**<br>CON: 178.3 ± 12.8<br>PLA: 179.6 ± 11.2<br>CMR: 175.7 ± 16.3
16	Carter 2004	Design: RCT<br>Location: UK	Endurance trained volunteers (n=9)<br>(7 male and2female, age 24.0± 3.8 yr, 70.5± 5.9 kg)	**Duration**: 2 weeks (2 experimental trials)<br><br>**Exercise protocol:**<br><br>After a brief 5min warm-up, participants performed a predetermined amount of work (914 ± 40 kJ) as fast as possible on a cycle ergometer.<br>The intensity was set to correspond to 75% of their maximal power output (Wmax), with a self-selected cadence of approximately 80–100 rpm.	4h fast before the visit<br><br>carbohydrate-rich meal the evening before<br><br>Average carbohydrate intake in the 24 hours before testing was 347 ± 25 g.	**Volume**:25ml<br>**Solutions:**<br><br>1) CHO :6.4% maltodextrin<br>2)PLA ,water<br><br>**Timing:** every 12.5% of the time trial completed,<br>**Rinsing duration**:5 sec	**Performance time**<br>CHO 59.57 ± 1.50min<br>PLA 61.37 ± 1.56min<br>(average improvement of 2.9% in the CHO trial P 0.003)<br>**Power output**<br>CHO 59.57 ± 1.50min<br>PLA 61.37 ± 1.56min<br>(CHO>PLA)<br>**HR(bmp)**<br>CHO 259 ± 16 W<br>PLA 252 ±16 W<br>(no difference in heart rate response in the two trials)
17	Cherif 2018	Design: RCT<br>Location: Qatar	Healthy Adults, Male (=15); Age 30.7±4 years; Weight 82.8±5 kg; Height: 181.0±6 cm<br><br>**Participants** Frequency of exercise 4.6±1.0 times/week	**Duration**: 4 sessions<br>**Type**: On an instrumented non-motorized treadmill with embedded force sensors; 10 min warm-up on the treadmill; Repeated-Sprints (RS); RS-protocol [RSP; 2 sets (SET1 and	**Duration**: 4 sessions<br>**Type**: 3 days of intermittent fasting abstaining from food and fluid 14 h per day	**Type**: 10 mL of solution for 5s<br>**Intervention**: CHO-MR 10% maltodextrin; The 10% CHO-MR solution (10% w/v) contained 12 g of commercially available maltodextrin powder dissolved	Maximal Power (w/kg) mean±SD<br>**Intervention:**<br>**Placebo:**<br>**Control:**<br>Maximal Speed (m/s) mean±SD<br>**Intervention:**<br>**Placebo:**

			*a randomized and counter-balanced manner	SET2) of 5×5 s maximal sprints with 25 s rest; 3 min of recovery between SET1 and SET2; 10 to 13 min of interval treadmill- sprints<br>**Days/week**: The experimental trials were separated by 15 days each<br>**Intensity**: maximal sprints		in a final volume 120-ml of distilled water.<br>**Placebo:** PLA-MR; 0% maltodextrin; The PLA-MR solution was obtained from a commercially avail- able non-caloric effervescent tablet one tablet per 1000 ml of distilled water<br>**Control:** CON; no-MR	**Control:**<br><u>Vertical Stiffness (kN/m)</u> mean±SD<br>**Intervention:**<br>**Placebo:**<br>**Control:**<br><u>Mid-RPE mean±SD</u><br>**Intervention:** 5.15±2.13<br>**Placebo:** 4.80±1.21<br>**Control:** 5.00±1.56<br><u>Post-RPE mean±SD</u><br>**Intervention:** 5.53±1.51<br>**Placebo:** 5.50±1.40<br>**Control:** 5.87±1.64<br><u>HR-Pre (bpm) mean±SD</u><br>**Intervention:** 76.0±10.1<br>**Placebo:** 74.5±9.8<br>**Control:** 81.3±15.3<br><u>HR-Post (bpm) mean±SD</u><br>**Intervention:** 135.1±14.9<br>**Placebo:** 136.4±17.4<br>**Control:** 136.1±14.5<br><u>RTI Movement time (ms) mean±SD</u><br><u>Pre</u><br>**Intervention:** 373.6±286.7<br>**Placebo:** 269.7±136.8<br>**Control:** 302.5±196.9<br><u>Post</u><br>**Intervention:** 207.8±86.0<br>**Placebo:** 231.9±84.9<br>**Control:** 223.2±86.3<br><u>RTI Reaction time (ms) mean±SD</u><br><u>Pre</u><br>**Intervention:** 299.9±45.2<br>**Placebo:** 279.0±52.1<br>**Control:** 294.9±36.8<br><u>Post</u><br>**Intervention:** 280.4±35.9<br>**Placebo:** 296.0±46.2<br>**Control:** 305.4±55.5

18	Chiu 2022	Design: RCT<br>Location: Taiwan	Trained taekwondo athletes(n=13)<br>(age: 20.9 ± 1.0 years; height: 176.7 ± 4.6 cm; weight: 69.8 ± 6.9 kg; % body fat: 11.6% ± 3.8%)<br><br>**Experience**:≥6 years of professional taekwondo training	**Duration:** within a month, about 3 weeks(2 experimental trials)<br><br>**Exercise protocol** :<br>Taekwondo-specific high-intensity protocol, including a warm-up (jogging, dynamic stretching, short sprints), taekwondo performance tests (vertical jump, loaded jump, reverse hook kick speed, FSKT), a simulated competition (three rounds of 5-s Wingate sprints with 25-s rests), and post-exercise assessments (vertical jump, kick speed, fatigue index).	On the experiment day, breakfast (692 kcal; 50% carbs, 32% fat, 18% protein) and lunch (829 kcal; 50% carbs, 30% fat, 20% protein) to ensure equal calorie intake.<br><br>Lunch about 3 hours before	**Volume**:25ml<br>**Solutions:**<br><br>1)CHO: 6.4% maltodextrin<br>2)CON: mineral water<br><br>**Timing:** after the warm up and after the specialty tests of taekwondo<br><br>**Rinsing duration :15 sec**	**HR**<br><u>BASELINE(ROUND 1)</u><br>CHO 161.6±10.2<br>CON 162.3±11.7<br>P 0.575<br><u>POST(ROUND 3)</u><br>CHO 164.1±13.7<br>CON 165.2±11.6<br>P 0.929<br>**The kicking number of frequency speed**<br><u>BASELINE(PRE-MATCH)</u><br>CHO 114.1±53.0<br>CON 107.1±51.3<br>P 0.347<br><u>POST(POST-MATCH)</u><br>CHO 101.3±46.5<br>CON 94.1±45.5<br>P 0.477<br>(not significantly different)<br>**Countermovement jump height**<br>CHO trial was no different than that of the CON trial on pre-match (P = 0.388) and post-match (P = 0.530)<br>**Kicking speed of the dominant foot and non dominant foot**<br>no difference on pre-match and post-match of dominant foot (P = 0.311 and P = 0.352) and of non-dominant foot (P = 0.382 and P = 0.507)<br>**Peak power and average power**<br>No significant differences were found between trials in peak or average power across rounds 1–3 (all p > 0.05).
19	Chong 2011	Design: RCT<br>Location: Australia	Trained male cyclists (n=14)<br>(mean±SD age: 28.7±4.0	**Duration:** 4 weeks(4 experimental trials)	overnight fasting	**Volume:**25ml	**POWER OUTPUT(W)**

			years, height: 178.3±6.4cm,body mass 74.8±6.9kg)	**Exercise protocol:**<br>30-second maximal "all-out" cycling sprint<br>Performed from a standing start, without pacing		**Solutions:**<br>1) CHO (6.4% (w/v) maltodextrin)<br>2) GLU (7.1% (w/v) glucose)<br>3) WA (water)<br>4)CON(no mouth rinse)<br><br>**Timing:** immediately before exercise.<br>**Rinsing duration:** 5 sec	(Mean relative change from Con (%))<br>BASELINE(P 0-30)<br>CON 854±19<br>GLU 855±18 (−1.40)<br>MAL 859±21 (-1.29)<br>WA 855±18 (-0,24)<br>POST(P 20-30)<br>CON 695±15<br>GLU 697±17 (+0.21)<br>MAL 699±16 (+0.57)<br>WA 696±14 (+0.10)<br>PMAX<br>CON 1206±39<br>GLU 1189±29 (-1.40)<br>MAL 1191±35 (-1.29)<br>WA 1203±30 (-0.24)<br>All of them were similar between Glu,Mal,Wa and Con trials (p>0.05)
20	Chryssanthopoulos 2018	Design: RCT<br>Location: Greece	Female recreational runners (n=15)<br>5 postmenopausal and 10 premenopausal<br>(Age 43.1 ± 2.2 years, body mass 59.2 ± 2.0 kg, height 167 ± 0.0 cm)<br><br>**Experience:** running actively for at least 3 years, be involved in endurance training on a regular basis and have participated at least in one race of ≥1-hour duration within the last 3 months.	**Duration:2 weeks (2 experimental trials)**<br>**Exercise protocol:**<br>10 min warm-up (7min running,3 min stretching). Then 1-hour maximal distance run on a 216-m indoor track.	No high-carbohydrate loading the days before<br><br>10 hours overnight fast<br><br>Standardized pre-race meals<br><br>After the second meal and until race start (~8 hours), only water was allowed.	**Volume**:25ml<br><br>**Solutions:**<br>1)CHO (6.4% maltodextrin)<br>2)PLA(~0% CHO)<br><br>**Rinsing duration**: 5 sec<br><br>**Timing:** Immediately before the 1-hour run and at 15, 30, and 45 minutes during the run and expectorated	**Distance covered in 60min race (Mean ±SE)**<br>CHO 10,621.88 ± 205.98<br>PLA 10,454.00 ± 206.64<br>(no difference ,RCHO vs RPLA (t =1.784,P = 0.096)<br>**HR (Mean ±SE)**<br>BASELINE(15MIN)<br>CHO 166±3<br>PLA 165±2<br><br>POST(60MIN)<br>CHO 178±31<br>PLA 174±31<br><br>(no interaction effect between treatments (RCHOvs RP) and time (15-60min)

21	Clarke 2017	Design: RCT<br>Location: UK	Recreationally resistance-trained males(n=12, mean ± SD age: 23±3 years, height: 175.5 ±7.4 cm, body mass: 75.4±7.5 kg)<br>**Experience:** trained three to four times a week for between 8 and 12 months,	**Duration:** 3 weeks(3 experimental trials)<br><br>**Exercise protocol:** Consisted of a countermovement jump (CMJ) with 3 vertical jumps to measure jump height, an isometric mid-thigh pull with a maximal 5-second effort and 90-second rest between attempts, a 10 m maximal sprint from a standing start, and bench press and back squat exercises at 60% of 1-RM performed to failure with 5 minutes of rest between exercises.	500 mL of water 60 minutes prior to arrival<br><br>11 hour overnight fast.	**Volume:**25ml<br>**Solutions:**<br>1)CHO :6% maltodextrin<br>2)PLA: water<br>3)CO: no mouth rinse<br>**Rinsing duration**: 10 sec<br>**Timing:** Prior to each exercise<br>,	**HR**<br>Baseline(pre-rinse)<br>CHO 77± 7<br>PLA 80± 11<br>CON<br>Post-intervention(post-rinse)<br>CHO 86± 8<br>PLA 88± 14<br>CON 81± 7<br><br>**Jump height**<br>Significantly higher following CHO and PLA compared with CON<br><br>Trivial difference in jump height between CHO and PLA<br><br>67% of the participants had a greater CMJ height in the CHO trial.<br>**Sprint**<br>significantly faster following CHO compared with CON and PLA<br><br>significantly faster following PLA than CON<br><br>58% of the participants were faster following CHO rinsing<br>**Peak force**<br>There were no significant differences between CHO and CON ,CHO and PLA and PLA and CON<br>**Bench press**<br>CHO was significantly greater than PLA and CON<br>**Squat repetitions**<br>more squat repetitions were performed following CHO and PLA compared with CON

							significantly higher number of squat repetitions were observed following CHO compared with PLAand CON
22	Clarke 2017	Design: RCT<br>Location: UK	15 recreational running people (men = 9; mean 6 SD age; 42 ± 10 years; height, 177.6 ±6.1 cm; body mass, 73.9 ± 8.9 kg) and (women = 6; mean ±SD age, 43 ± 9 years; height, 166.5 ± 4.1 cm; body mass,65.7 ± 6.8 kg<br><br>**Experience**:running training for the past 2years ,familiar with a 5k running	**Duration**: about 1,5week (4 experimental trials)<br>**Exercise protocol**: 5 minute warm-up of low-to-moderate jogging followed by 5 minutes of self-selected stretching, which was replicated across trials. Participants were verbally encouraged to complete the 5-km time trial as fast as possible on a track.<br>**Conditions**: 19.4± 0.5 C , humidity 64.0 ± 0.8%	a standardized diet 24 hours before each trial<br><br>5-hour fasting before the trial	**Volume:**25ml<br>**Solutions**<br>1)PLA: 0% (placebo),<br>2)3%maltodextrin<br>3)6% maltodextrin<br>4)12% maltodextrin<br><br>**Timing**: Immediately before starting the time trial and then after each 1 km(total 5 rinses per trial)<br>**Rinsing duration:**10sec	**Performance time(mean ±SD)**<br><u>Men</u><br>0%, 27:02 ± 4:02 min<br>3%, 27:49 ± 4:34min<br>6%, 27:47 ± 3:59min<br>12%, 27:25 ± 4.29min<br><u>women</u><br>0%, 25:50 ± 4:31min<br>3%, 26:29 ± 4:49min<br>6% 26:02 ± 3:46min<br>12%, 25:50 ± 4.49min<br>there was no significant difference in 5-km TT performance time between men and women, there was a nonsignificant interaction between sex and 5-km TT performance time)<br>**HR(mean ±SD)**<br>0% 160 ±9<br>3% 154 ±12<br>6% 155 ±13<br>12% 153± 11<br>No significant differences were observed (p = 0.095; P = 0.16)
23	Clarke 2015	Design: RCT<br>Location: UK	Healthy Adults, Male (n=15); Age 21 ± 2 years; Weight 77 ± 6 kg; Height 176 ± 5 cm<br><br>**Participants** Taking part in upper-body resistance training at least once a week and for a minimum of 6 months<br><br>*a double- blind, randomized,	**Duration**:<br>**Type**: A 1 repetition maximum (RM) bench press followed by 60% of their 1RM to failure<br>**Days/week**: minimum 2 days to avoid fatigue<br>**Intensity**: repetitions completed at the 60% of 1MR until failure<br><br>Subjects were instructed to refrain from strenuous exercise 24 hours before the trials	**Duration**:<br>**Type**: Subjects were instructed to avoid CAF ingestion for a minimum of 12 hours before the trials	**Type**: Before exercise, 25 ml solution mouth rinse for 10 sec.<br>**Intervention**: 6% (15 g: 0.20 ± 0.02 g ·kg $^{-1}$) CHO solution Maltodextrin<br>**Placebo**: Water<br>All solutions were flavored with 0.8% (200 mg) sucralose	<u>Heart rate (bpm)</u><br>**Intervention** mean±SD<br>152 ± 19<br>**Placebo** mean±SD<br>151 ± 27<br>**Control** mean±SD<br>149 ± 24<br><br><u>RPE</u><br>**Intervention** mean±SD<br>17 ± 3<br>**Placebo** mean±SD<br>18 ± 2

			counterbalanced crossover design				**Control** mean±SD<br>17 ± 2
24	Cramer 2015	Design: RCT<br>Location: Qatar	Well-trained male cyclists (n=83,age 1.4 ± 4.1 y, weight75.3 ± 6.3kg)<br><br>**Experience:** cycling a minimum of 250 kmwk−1 for 6 week prior and were well-practized in time trial competitions	**Duration**: ~2 weeks(2 experimental trials)<br>**Exercise protocol**: a distance of 40 km as quickly as possible.<br>**Conditions**: environmental chamber at 35◦C and 60% humidity)	~3h fast<br><br>300ml of water upon arrival at the laboratory.<br><br>water during all testing	**Volume**:20-ml bolus of 6.5g100ml−1<br>**Solutions**:<br>1)maltodextrin<br>2) PLA<br>**Timing**: at 5-km intervals of the time trial<br>**Rinsing duration**:5s	**Time trial completion time (min)(Mean±SD)**<br>CHO: 63.9 ± 3.2 min<br>PLA: 64.3 ± 2.8 min<br>P = 0.57 (no significant difference)<br>**Performance difference (%)**<br>CHO vs PLA: 0.62 ± 2.93% faster<br>**HR(Mean±SD)**<br>increased over time<br>CHO 184 ± 3 beatsmin−1<br>PLA 184 ± 4 beatsmin−1 in<br>(P < 0.0)<br>No treatment-by-time interactions were found for heart rate (P = 0.18
25	De Oliveira 2020	Design: RCT<br>Location: Brazil	University futsal players (n=14)(age = 20.85 ± 1.46 yrs; body mass = 77.07 ± 6.94 kg; height = 1.77 ± 0.83 cm; BMI = 24.4 ± 2.60<br>**Experience:** at least 1 year with the sport	**Duration:**8 days(8 experimental trials)<br><br>**Exercise protocol:** Eight experimental conditions were performed before the repeated sprint ability (RSA) test, including conditions with and without a post-activation potentiation (PAP) protocol. When applied, the PAP protocol consisted of one set of three back squat repetitions at 90% of one-repetition maximum (1RM), followed by a 7-min recovery period. Subsequently, participants completed the RSA test, consisting of 6 × 40 m sprints (20 + 20 m) with 20 s of passive recovery between sprints.	no food for at least 2 hrs before	**Volume:**25 mL<br>**Solutions:**<br>1)CHO (6% (w / v) maltodextrin)<br>2)PLA(non-caloric sweetener (sucralose))<br>**Timing:**before undergoing a Repeat Sprints Test<br>**Rinsing duration:**10 or 40 sec(when PAP took place)	**Best sprint time(sec)(Mean ± SD)**<br>PLA CON 7.77 ± 0.28<br>CHO (10 sec) 7.79 ± 0.19<br>CHO (40 sec) 7.71 ± 0.15<br>PAP + PLA 7.21 ± 0.14<br>PAP + CHO (10 sec) 7.35 ± 0.15<br>PAP + CHO (40 sec) 7.38 ± 0.44<br>**Mean sprint time(sec)(Mean ± SD)**<br>PLA CON 8.18 ± 0.21<br>CHO (10 sec) 8.18 ± 0.37<br>CHO (40 sec) 8.15 ± 0.62<br>PAP + PLA 7.73 ± 0.20<br>PAP + CHO (10 sec) 7.73 ± 0.26<br>PAP + CHO (40 sec) 7.79 ± 0.20<br>**Total sprint time(sec)(Mean ± SD)**

							PLA CON 49.02 ± 0.82<br>CHO (10 sec) 49.12 ± 0.21<br>CHO (40 sec) 48.92 ± 0.36<br>PAP + PLA 46.39 ± 1.22<br>PAP + CHO (10 sec)46.40 ± 1.58<br>PAP + CHO (40 sec)46.78 ± 1.25
26	De oliveira 2019	Design: RCT<br>Location: Brazil	Male soccer players(n=20) (age = 18.9 ± 0.9 years; body weight = 71.8 ± 5.2 kg; height = 178.2 ± 6.3 cm<br>**Experience**: at least 2 years with their current sports	**Duration:** about 4 days (4 experimental trials)<br><br>**Excercise protocol:**<br>2 trials with PAP and 2 without The intervention consisted of a standardized warm-up(tradmill run at 60–70% of maxHR), followed by either a PAP protocol (two sets of five back squat repetitions at 80% 1RM) or a control condition without PAP, then a carbohydrate or placebo mouth rinse, and finally a repeated sprint ability (RSA) test with six sprints of 40 m with 20 s of passive rest between them	no food at least 2 h before	**Volume:**25ml<br>**Solutions:**<br>1)CHO (6% (w/v) maltodextrin)<br>2)PLA(non-caloric sweetener (sucralose))<br>**Timing:**7min after the PAP test and 1 minute before the RSA<br>**Rinsing duration:**10sec	**Best sprint time(sec)(Mean ± SD)**<br>PLA CON 7 .12 ± 0.26<br>CHO 7.14 ± 0.23<br>PAP-PLA 7.03 ± 0.24<br>PAP-CHO 7.00 ± 0.25<br>**Mean sprint time(sec)(Mean ± SD)**<br>PLACON 7.46 ± 0.29<br>CHO 7.47 ± 0.24<br>PAP-PLA 7.36 ± 0.27<br>PAP-CHO 7.35 ± 0.26<br>**Total sprint time(sec)(Mean ± SD)**<br>PLA CON 44.76 ± 1.73<br>CHO 44.80 ± 1.42<br>PAP-PLA 44.16 ± 1.63<br>PAP-CHO 44.12 ± 1.53<br>**Performance Decrement (%)(Mean ± SD)**<br>PLACON 4.80 ± 1.23<br>CHO 4.62 ± 1.32<br>PAP-PLA 4.74 ± 1.53<br>PAP-CHO 5.01 ± 1.53
27	Decimoni 2018	Design: RCT<br>Location: Brazil	Fifteen recreationally trained women (age 26 ± 4y; height 1.61 ± 5m, weight 59 ± 8kg)<br>**Experience:** 1-2 years strength training	**Duration:**3 days (2 experimental trials)<br>**Exercise protocol:**<br>Warm up and then two resistance exercise bouts consisting of three sets of five	8h overnight fast<br><br>Standarized breakfast prior to each test	**Volume:**100ml<br><br>**Solutions:**<br>1)CHO:6%maltodextrin<br>2)PLA: arificially flavored with aspartame and saccharin<br>**Rinsing duration**:10 sec	**Volume Workload**<br>CHO 7.589 1.914<br>PLA 6.678 1.1741<br>p=0.039<br>no difference between CHO and PLA conditions,12% greater after the CHO session than

				exercises (half-squat, leg press, bench press, military press, and seated row) to volitional fatigue with a 10 repetition-maximum load and 2 min rest between sets		**Timing:** at the commencement of each experimental trial and immediately before performance of the BP exercise	
28	Deighton 2016	Design: RCT<br>Location: UK	Eighteen healthy men (mean(SD); age 23(4)years, body mass index 23.1(2.4)kg.m2)	**Duration:**3 weeks(3 experimental trials)<br>**Exercise protocol:** 60 minutes of self-selected treadmill walking at a 1% incline, performed at a self-regulated intensity corresponding to RPE 13 ("fairly hard"), reached within 2 minutes and maintained by adjusting speed throughout the trial<br>**Conditions:** 20 ºC and 39 % humidity	Overnight fast (at least 10h)	**Volume:**25ml<br>**Solutions**<br>1)CHO(6.4% maltodextrin sweetened with saccharin)<br>2)WAT (water)<br>3)PLA (sweetened with saccharin)<br>**Rinsing duration:**10sec<br>**Timing:** at 10, 5, 0, and every 7.5 min thereafter throughout the 60min walk	**Total walking distance :** 5814 m<br>no significant difference between CHO, PLA, and WAT<br>**CHO vs WAT:**<br>Mean difference: +163 m<br>Percentage difference: +2.9%<br>Probability of benefit/trivial/harm: 86.5% / 12.1% / 1.4%<br>**PLA vs WAT:**<br>Mean difference: +134 m<br>Percentage difference: +2.3%<br>Probability of benefit/trivial/harm: 84.4% / 14.7% / 0.9%<br>**CHO vs PLA:**<br>Mean difference: +29 m<br>Percentage difference: +0.5%<br>Unclear effect
29	Devenney 2016	Design: RCT<br>Location: Ireland	Healthy Adults, Male (n=12); Age 22 ± 7 years; Weight 69 ± 9 kg; Height 1.75 ± 0.07 cm; BMI 22 ± 1.7 kg/m2<br>**Participants** Active cycling for recreational purposes; All of whom had engaged in cycling activities 3–5 days a week/5–8 h per week<br>*a randomized, repeated measures and double blinded study	**Duration**: 4 visits<br>**Type**: All tests carried out on a cycle ergometer; 5 min warm-up at approximately 40% of Wmax; Individuals completed a set amount of work, which was individualized and calculated based on their maximum work capacity, in the shortest timeframe possible;1 h work;<br>**Days/week**:<br>**Intensity:** 65% TO 75% aerobic capacity test due to recreational nature of the participants	**Duration**: 4 visits<br>**Type**: Participants were asked to refrain from consuming alcohol and caffeine in the 24 h prior to each visit; Participants consumed a meal 2–3 h before testing 49 ± 2% carbohydrates, 18 ± 1% protein and 33 ± 2% fat;	**Type**: A bolus was provided to the participant after the warm-up and every 12.5% of completion in the trial for 5 sec mouth rinse; 25 ml solution<br>**Intervention**: Participants rinsed a 6% or 16% CHO solution at predetermined intervals<br>**Placebo**: 0% CHO	Performance time and power 6%<br>**Intervention CHO** mean±SD<br>58.8 ± 7.0 min<br>Performance time and power 16%<br>**Intervention CHO** mean±SD<br>57.9 ± 7.6<br>Performance time and power<br>**Placebo** mean±SD<br>62.3 ± 7.6 min<br>Average power 6%<br>**Intervention** mean±SD<br>174±20W<br>Average power 16%<br>**Intervention** mean±SD

				They were allowed to undertake low-intensity exercise (heart rate below 150 beats/ min) for up to 2 h two days prior trail			163±23W<br><u>Average power</u><br>**Placebo** mean±SD<br>177 ± 23 W<br><br><u>Heart rate 6%</u><br>**Intervention** mean±SD<br>171 ± 19<br><u>Heart rate 16 %</u><br>**Intervention** mean±SD<br>174 ± 14<br><u>Heart rate</u><br>**Placebo** mean±SD<br>168 ± 18
30	Dorling 2013	Design: RCT<br>Location: UK	Physically active males (n=8)<br>(Age; 22 ± 1 y; body mass 75.0 ±8.8 kg)<br><br>**Experience**:(n=7) field based sports(f.e.football),the other were recreationally active runners)	**Duration**:2 weeks to ~2,5weeks(2 experimental trials)<br><br>**Exercise protocol: s**occer-specific intermittent running protocol (LIST) combined with repeated sprint ability (RSA) tests. After a standardized warm-up, participants performed the LIST, which included 15-min sections of intermittent 20-m shuttle running (walking, jogging, cruising, and sprinting at prescribed speeds), with 3-min recovery between sections. RSA tests were performed before the LIST and after the 2nd and 4th sections, with short recovery periods.	568 ml of water the day of the trial<br><br>Arrived on a fasted condition**.**	**Volume**:25 ml<br>**Solutions:**<br>1)CHO (6.4% maltodextrin)<br>2)PLA(water).<br><br>**Rinsing duration:**~5sec<br><br>**Timing:** before every LIST and RSA test.(total 27 rinses)	**Fastest RSA sprint time**<br>PLA: 3.38 ± 0.2 s<br>CHO: 3.37 ± 0.2 s<br>p = 0.39<br>**Mean RSA sprint time**<br>PLA: 3.46 ± 0.19 s<br>CHO: 3.44 ± 0.17 s<br>p = 0.49<br>**Mean sprint time during LIST**<br>PLA: 3.52 ± 0.2 s<br>CHO: 3.54 ± 0.2 s<br>p = 0.63<br>**Effect size (sprint measures)**Effect sizes were trivial (≤ 0.2).
31	Dolan 2017	Design: RCT<br>Location: USA	Healthy Adults, Male (n-14); Age 19.9±1.3 years; BM 84.9±15.7 kg; Height: 182± 9 cm; Body fat 11.3±3.8%	**Duration**: 5 test<br>**Type**: Yo-Yo IRT-1 test (Intermittent Recovery Test-Level 1); Running lanes with a width of 1.5 m and a length of 20 m<br>**Days/week**: 5	**Duration**: 5 tests<br>**Type**: The tests were performed following an overnight fast (10 hours); Recorded their food intake in the 24 hours preceding the experimental trial	**Type**: 25 ml solution and 10 sec moth rinse; Each week, the Yo-Yo IRT-1 began immediately after completing the mouth rinsing protocol.<br>**Intervention**: Solution of carbohydrate (CHO) 6%	<u>RPE</u><br>**Intervention** mean±SD<br>18.6 ± 1.3<br>**Placebo** mean±SD<br>18.6 ± 1.4<br>**Control** mean±SD<br>19.1 ± 1.0

			**Participants** NCAA Lacrosse players; All participants were familiar with intense intermittent exercise *a double-blind, randomized crossover design	separate occasions over a 29-day period. **Intensity**: Progressively increasing speeds controlled by auditory cues Participants avoided strenuous exercise in the 12 hours preceding each experimental trial		**Placebo**: (H2O) flavored water; **Control:** no rinse control (CON) condition.	Meters complete **Intervention** mean±SD 1,480 ± 253 m **Placebo** mean±SD 1,397 ± 360 m **Control** mean±SD 1,436 ± 292 m
32	Durkin 2021	Design: RCT Location: UK	Resistance-trained young men(n=12) (age: 22±4 years; height: 1.79±0.05m; weight: 78.7±7.8kg **Experience**: a minimum of two years	**Duration:**2 days(2 experimental trials**)** **Exercise protocols:** The session was conducted after a glycogen-depleting exercise, that took place the day before Participants performed a warm-up of 15 repetitions per exercise at 20 kg, followed by six sets of bench press and six sets of squats to failure at 40% 1-RM. Repetitions were performed at a self-selected pace without pausing, with 2 minutes of rest between sets and 5 minutes of rest between exercises.	minimum 2-hour fast standardized, energy restricted and low carbohydrate breakfast (14.4g CHO) 68.5g CHO consumption the last ~16h	**Volume:**25ml **Solutions:** 1)CHO (6.4% maltodextrin) 2)PLA **Timing:**30sec before each set of lifting **Rinsing duration:**10sec	**Total Volume Load (Resistance Training Capacity)** CHO: 9354 ± 2051 kg PLA: 8525 ± 1911 kg p = 0.010 Effect size: d = 0.418 Significantly greater workload with CHO. **Bench Press Performance:** CHO: 120 ± 24 repetitions PLA: 115 ± 22 repetitions p = 0.146 Effect size: d = 0.198 No significant difference. **Squat Performance:** CHO: 107 ± 26 repetitions PLA: 92 ± 16 repetitions p = 0.017 Effect size: d = 0.685 95% CI: 3.1–26.2 repetitions Greater repetitions with CHO.
33	Dunkin 2017	Design: RCT Location: UK	Twelve recreationally resistance-trained men (age: 22±1 years, height 179.2±1.8 cm, body mass 80.9±6.1 kg)	1RM and repetitions to failure protocols were conducted on a bench press rack. 40% 1RM	Subjects were asked to refrain from exercise, alcohol, and caffeine intake for 24 hours before each session, to complete a 24-hour dietary record before the first testing session, and to replicate this diet for 24 hours before each subsequent session to standardize endogenous energy content. Adherence to	An 18% maltodextrin (Bulk Powders maltodextrin; Bulk Powders TM, Colchester, United Kingdom) solution (CHO) and a water placebo solution (PLA).	CHO mouth rinse does not significantly affect maximal muscular strength or endurance

					these procedures was verbally confirmed at the beginning of each trial.		
34	Evans 2021	Design: RCT<br>Location: USA	33 participants; 16 males and 17 females. Adults, age ≥ 70 years, and were able to ambulate unassisted.<br>(age: 78±5 years, height 166±10 cm, body mass index 29±4 kg)	6-minute walking test, 2 trials	No specific dietary instructions. All participants were at least 4 h post-prandial and had refrained from caffeine and alcohol intake for at least 12 h prior to each session	CHO (6.4% maltodextrin) condition and a placebo condition	Mouth rinsing with a 6.4% maltodextrin CHO solution did not significantly impact 6-minute walking test performance
35	Ferreira 2018	Design: RCT<br>Location: Brazil	Male trained cyclists(n=11)<br><br>(Age (years)30.4 ± 6.2 Body mass (kg)73.6 ± 7.8 Height (m)1.73 ± 0.08 Body Mass Index (kg/m2)24.7 ± 2.2 Body Fat Percentage (%) 11.2 ± 3.4)<br>**Experience:** cycling for at least 150 km about 5 h/wk	**Duration:** not specified (3 experimental trials)<br>**Exercise protocol:**30-km cycling time-trial performed on a computer-coupled cycle ergometer, with self-selected cadence and maximal effort to complete the distance as fast as possible<br>**Conditions:** average temperature of 22.1 °C and humidity of 72.5%	2–3 h from the last meal<br>CHO 74.6±20.1g<br>PLA 62.4±12.9g<br>DAL 68.2±18.8g<br>P 0.107<br>No significant difference was observed for energy and nutrients in 24 h and in the meal before test	**Volume:**25ml<br>**Solutions:**<br>1)CHO (6.4% of unflavored maltodextrin)<br>2)PLA<br>3)DAL (water, drinking “ad libitum”)<br>**Timing:** after every 3.75 km (12.5% of the time trial completed<br>**Rinsing duration:**10sec	<u>**Performance in cycling 30km**</u><br>**Time to complete(min)**<br>CHO 54.5±2.9<br>PLA 54.7±2.9<br>DAL 54.5±2.5<br>p 0.82<br>No difference between interventions.<br>**Avarage power output (W)**<br>CHO 198.6±25.9<br>PLA 196.6±26.9<br>DAL 196.9±22.4<br>p 0.84<br>No difference between conditions.<br>**Avarage speed(km/h)**<br>CHO 33.1±1.3<br>PLA 32.9±1.8<br>DAL 32.9±1.5<br>P 0.84<br>No significant difference.<br>**Avarage heart rate(bpm)**<br>CHO 153.9±13.0<br>PLA 150.1±13.6<br>DAL 153.7±16.5<br>P 0.38<br>No difference between conditions
36	Ferreira 2018	Design: RCT<br>Location: Brazil	Recreationally trained male cyclists(n=11)<br>(Age = 30.4 ± 6.2 yr; height = 1.73 ± 0.08 m; weight = 73.6 ±	**Duration:**~8 days(3 experimental trials)<br>**Exercise protocol:** 30 km time trial cycle ergometer with self-reg	500 ml of fluid two hours before the tests	**Volume:** 1)CHOMR25ml<br>2)DWAL 150ml<br>3)DAL 150ml<br>**Solutions:**	**Mean intensity of 30km trials**<br>CHOMR 81.3 ± 5.8<br>DWL 82.5 ± 7.9<br>DAL 81.1 ± 7.4

			7.81 kg; body mass index = 24.67 ± 2.22 kg/m²; Body fat percentage = 11.2 ± 3.4%)<br>**Experience:** at least 150 km for 5 hours a week and were heat acclimated	ulated pace in the shortest time possible<br>**Conditions**: average temperature of 22.1º C and humidity of 72.5%	Pre-exercise meal 2-3 hours before each trial	1)CHOMR(6.4% of unflavored maltodextrin, mouth rinse)<br>2)DWL (drinking to replace all weight loss),if the weight loss was higher than 2%, 1 electrolyte capsule containing 321 mg of sodium (14 mEq) was used<br>3)DAL(drinking "ad libitum")<br>**Timing:** after every 12.5% of the 30 km time trial completed(for the MR)<br>**Rinsing duration:**10sec(for the MR)	not significant different, p = 0.60<br>**Average power**<br>CHOMR 198.6 ± 25.9W<br>DWL 210.7 ± 41.3 W<br>DAL 196.9 ± 22.4 W<br>not significant different, p = 0.25<br>**Average velocity**<br>CHOMR 33.1 ± 1.7<br>DWL 33.8 ± 2.5<br>DAL 33.0 ± 1.5 km·h-1<br>not significant different, p = 0.06<br>**Time to complete 30km trial**<br>CHOMR 54.5 ± 2.9min<br>DWAL 53.6 ± 4.0min<br>DAL 54.5 ± 2.5 min<br>not significantly different, p=0.131<br>**Maximal HR**<br>No significant difference among interventions, p = 0.24
37	Fares 2011	Design: RCT<br>Location: Lebanon	Healthy Adults, Male (n=13); Age 21 ± 3 years; BM 83 ± 8 kg; Height 180 ± 6 cm<br>**Participants** non-athletic<br>*a randomized balanced order and the subjects were blinded to the mouth-rinse solution.	**Duration**: 5 sessions<br>**Type**: In a cycle ergometer; After 5min warm-up at 50 Watts, power was set at 60% Wmax and subjects would cycle until exhaustion.<br>**Days/week**: Time between tests was not less than 72hrs and not more than 96hrs<br>**Intensity**: Subjects did the time-to-exhaustion trials at 120 ± 11 Watt (60% Wmax).	**Duration**: 5 sessions<br>**Type**: One time after an overnight fast (FCHO and FPLA) and another after a carbohydrate rich breakfast (CHO and PLA); Breakfast included sandwiches/ cereals/oatmeal along with fat-free milk/fruit juice (no coffee or tea), fresh fruits (bananas/figs), and dried fruits (raisins/ apricots/dates). Breakfast was taken 3 hours before the test.	**Type**: Subjects rinse their mouth with a 25 ml solution for 5-10 sec every 5 min<br>**Intervention:** Maltodextrin solution 6.4%, CHO<br>**Placebo**: Just plain water	Time-to-exhaustion mean±SD<br>FCHO 53.9 ± 12.8<br>FPLA 48.3 ± 15.3<br>CHO 56.6 ± 12.2<br>PLA 54.7 ± 11.3<br>Mean HR mean±SD<br>FCHO 155 ± 10<br>FPLA 152 ± 9<br>CHO 152 ± 7<br>PLA 159 ± 8<br>Max HR mean±SD<br>FCHO 166 ± 12<br>FPLA 165 ± 9<br>CHO 164 ± 9<br>PLA 172 ± 9<br>Mean RPE mean±SD<br>FCHO 5.2 ± 0.9<br>FPLA 5.4 ± 0.8

							CHO 5.0 ± 0.7<br>PLA 5.5 ± 0.7<br>Mean RPE mean±SD<br>FCHO 8.4 ± 1.0<br>FPLA 8.9 ± 0.2<br>CHO 8.6 ± 0.5<br>PLA 8.9 ± 0.4
38	Fraga 2015	Design: RCT<br>Location: Brazil	Healthy Adults, Male (n=6); Age 26 ± 6 years; Weight 73.9 ± 7.6 kg; Height 177± 6.8 cm<br><br>**Participants** Well-trained subjects<br><br>*a counterbalanced, randomized double-blind design.	**Duration**: 4 sessions<br>**Type**: Exercise at a treadmill; Subjects performed an exercise in which treadmill velocity was adjusted corresponding to 85% of VO2max at 1% gradient and remained constant<br>**Days/week**: an interval of 7 days<br>**Intensity**: 85% of VO2max<br>Abstained from exercise in the previous 24 h	**Duration**: 4 sessions<br>**Type**: Overnight fast; abstained from alcohol, caffeine in the previous 24 h;	**Type**: Subjects received 25 ml of MR or PLA; mouth rinse 10 sec; Individuals received the solutions 15 min prior to the test and every 15 min during it.<br>**Intervention:** The carbohydrate mouth rinse (MR) consisted of an 8% dextrose solution<br>**Placebo:** Was a commercially available non-caloric sweetener, not different from MR in flavour and appearance	Time (s) mean ± SD<br>**Intervention:** 2625 ± 804<br>**Placebo:** 1935 ± 809<br>Distance (m) mean± SD<br>**Intervention:** 9855 ± 4119<br>**Placebo:** 7295 ± 3727<br>HR (bpm) mean ± SD<br>Rest<br>**Intervention:** 63 ± 9<br>**Placebo:** 62 ± 11<br>30 min:<br>**Intervention:** 185 ± 10<br>**Placebo:** 190 ± 9
39	Green 2022	Design: RCT<br>Location: USA	Resistance-trained **men and women** (N = 36; **18 male / 18 female**); age **21.5 ± 1.6 y**, height **1.72 ± 0.09 m**, body mass **72.8 ± 13.4 kg**, body fat **16.7 ± 5.8%**. Inclusion: **19–25 y**, “intermediate” resistance-training status (upper-body strength training **2–3×/week for ≥6 months**) and regular bench press use.	**Duration: Single acute session per trial** (3 experimental visits, **48–72 h apart**).<br>**Type / Intensity: Bench press protocol** — **4 × 10 reps at 65% 1RM** with **120 s seated recovery**, then (2 min after set 4) **1 set to momentary failure at 60% 1RM**.	**Duration: Overnight (12 h) fast** before each testing session.<br>**Type: No dietary intervention/standardized meal** (diet not controlled; participants followed habitual self-selected intake outside testing).	**carbohydrate rinse** — **25 mL** rinse for **10 s** (expectorated) **30 s before each set (sets 1–5)**.<br>•Placebo: **calorie-free sports beverage** (Powerade Zero).<br>• Carbohydrate: **6.4% maltodextrin** added to the same beverage.	**INTERVENTION (CHO mouth rinse)**<br>**Repetitions to failure (Set 5 60% 1RM), mean ± SD = 18.7 ± 4.8**<br>*(reported as 18.7 ± 0.8 SEM; SD calculated using SD = SEM × √36 = SEM × 6)*<br>**RPE (Borg 6–20): NR**<br>**Feeling Scale (−5 to +5): NR**<br>**Post-exercise blood glucose: NR**<br>**Post-exercise blood lactate: NR**<br>**PLACEBO (noncaloric placebo rinse)**<br>**Repetitions to failure (Set 5 @ 60% 1RM), mean ± SD = 19.0 ± 4.2**<br>*(reported as 19.0 ± 0.7 SEM; SD = 0.7 × 6 = 4.2)*<br>**RPE: NR**<br>**Feeling Scale: NR**<br>**Post-exercise blood glucose: NR**<br>**Post-exercise blood lactate: NR**

							**CONTROL (water rinse)**<br>**Repetitions to failure (Set 5 @ 60% 1RM), mean ± SD = 17.7 ± 4.8**<br>*(reported as 17.7 ± 0.8 SEM; SD = 0.8 × 6 = 4.8)*<br>**RPE: NR**<br>**Feeling Scale: NR**<br>**Post-exercise blood glucose: NR**<br>**Post-exercise blood lactate: NR**
40	Gam 2013	Design: RCT<br>Location: Australia	Moderately trained male cyclists (n=10)<br>(age 29.6 ± 6.6 years, weight 79.04 ± 10.80 kg)	**Duration**:3 weeks(3 experimental trials)<br>**Exercise protocol**:<br>a warm-up at a standarized power(100-200W)and then they completed a 1,000-kJ time trial as fast a possible	4-hr postprandial period during which no food was consumed	**Volume:**25ml<br>**Solutions:**<br>1)CHO (CHO)<br>2)WAT (plain water)<br>3)CON(no-rinse)<br><br>**Timing**: every 12,5% of the time trial completed<br>**Rinsing duration**: 5 sec	**Total time to completion(mean ± SD)**<br>CHO 65.7 ± 11.07 min<br>WAT 69.4 ± 13.81 min<br>CON 67.6 ± 12.68 min<br>WAT condition was significantly slower than both the CHO and CON.No significant difference in time to completion between CHO and CON<br>**Average Power output(mean ± SD)**<br>At 10sec rinse<br>CHO 250 ± 9.50<br>WAT 254 ± 11.19<br>CON 258 ± 9.69<br>Midway<br>CHO 259 ± 9.66<br>WAT 260 ± 11.21<br>CON 255 ± 10.81<br>**Effect sizes:**CHO: 0.53<br>WA: 0.49<br>CON: –0.36<br>**HR**<br>did not differ between the three expermental treatments. Increased steadily, reaching maximal values at 100% of the trial completed
41	Gam 2014	Design: RCT<br>Location: Australia	Healthy competitive male cyclists (n=12)	**Duration**:4 weeks(4 experimental trials)	Overnight fasting	**Volume**:25ml<br>**Solutions**:	**Mean power (mean ± SEM)**<br>WAT 885 ± 37

			(mean ± SD age 26 ± 3 years; body mass index 24 ± 3 kg/m2)	**Exercise protocol:** a 4-min light cycling warm up at 40 % of VO2peak followed by a 2–3-s practice start. The main part was a 30-s maximal cycling **Conditions:**. Laboratory temperature and relative humidity were similar between trials		1)WAT(plain water) 2)GLU:7,1% glucose solution 3)CON(no-rinse control) **Rinsing duration:** 10sec **Timing:**10 minutes after the warm-up and immediately before the exercise protocol	GLU 873 ± 42<br>CON 873 ± 39<br>Not significantly different between the 3 treatments(p>0.05)<br>**Peak power(mean ± SEM)**<br>WAT 1,220 ± 70<br>GLU 1,202 ± 59<br>CON1,208 ± 65<br>Not significantly different between the 3 treatments(p>0.05)<br>**HR**<br>similar at all time points between treatments (p>0.05)
42	Hawkins 2017	Design: RCT Location: USA	**Participants:** Trained endurance athletes (n=21; 12 men, 9 women), 18–45 years, BMI 18.5–24.9 kg/m²; trained ≥4 days/week for ≥1 hour/day. Women were tested in the follicular phase (days 3–9).	**Physical activity/exercise intervention Duration:** Acute exercise bout per visit; 12.8 km running time trial (≈60–67 min on average). 4 trials total, ≥4 days between trials. **Type / Intensity:** Self-paced "as fast as possible" 12.8 km TT (vigorous/high-intensity endurance effort)	**Duration:** Standardized pre-trial conditions: 8–12 h fast; standardized/replicated diet prior to each visit (evening meal repeated).	**Type:** 4 conditions (randomized crossover): sucrose (sweet + energy), sucralose 1:1 sweetness (no energy), sucralose 100:1 sweetness (no energy), water control. 25 mL rinse for 5 s, expectorate; at time 0 and every 12.5% of TT (8 rinses total).	**INTERVENTION — Sucrose mouth rinse (S)**<br>**Time Trial completion time (12.8 km): 63.47 ± 2.17 min**<br>(στο abstract: **1:03:47 ± 00:02:17**)<br>**Heart Rate (Average): 164.4 ± 2.5 bpm**<br>**PLACEBO — Sucralose mouth rinse**<br>**Sucralose 1:1 (S1:1)**<br>**Time Trial completion time: 64.55 ± 2.45 min**<br>**RPE (Max): 15.05 ± 0.39**<br>**Sucralose 100:1 (S100:1)**<br>**Time Trial completion time: 65.38 ± 2.12 min**<br>**RPE (Max): 15.81 ± 0.45**<br>**CONTROL — Water (C)**<br>**Time Trial completion time: 66.56 ± 2.18 min**<br>(στο abstract: **1:06:56 ± 00:02:18**)<br>**Heart Rate (Average): 159.7 ± 2.8 bpm**<br>**RPE (Max): 16.05 ± 0.44**
43	Hirata 2021	Design: RCT Location: Japan	Healthy Adults, Male (n-30); Age 21.3 ± 0.8 years; BM 69.6 ± 8.3 kg; Height 175.7 ± 5.6 cm	**Duration**: 3 trials **Type**: Muscle fatigue exercise, we used isometric handgrip exercises; 10-second MVC	**Duration**: 3 trials **Type**: They were instructed to refrain from, caffeine intake, and alcohol consumption 24	**Type**: Mouth rinsing was performed a total of six times, starting with the fifth set in the second half of the exercise	

			**Participants** College Students<br><br>*a randomized, single-blind, crossover design	followed by rhythmic grip movements of 40% MVC (2-second contraction, 2-second relaxation) 14 times per minute, followed by a 4-second rest period. Ten sets were performed. Finally, a final MVC measurement (the 11th measurement) signaled the end of the test<br>**Days/week**: The tests were spaced at least 7 days apart<br>**Intensity**: First baseline 10-second maximal voluntary contraction (MVC) and then followed by rhythmic grip movements of 40% MVC<br><br>They were instructed to refrain from strenuous physical activity 24 hours prior to testing	hours prior to testing and to finish eating at least two hours prior to testing;	protocol and ending with the tenth set; For mouth rinsing, 25 mL of solution was held in the mouth for 10 sec;<br>**Intervention**: A 6% glucose solution was used for GMR.<br>**Placebo**: Artificially sweetened mouthwash for PLA a 0.03% acesulfame solution<br>**Control**: no mouthwash	Mouth rinse improved maximal voluntary contraction
44	Hsu 2025	Design: RCT<br>Location: Taiwan	Fifteen trained female collegiate fast-pitch softball players (n = 15; age: 20.6 ± 0.9 years; height: 159.5 ± 5.2 cm; body mass: 58.1 ± 5.9 kg; training age: 6.8 ± 0.8 years). Inclusion: >6 years fast-pitch softball training; >6 months continuous training; from top four teams nationally.	**Duration:** Acute, single-session testing per condition (two trials total with ≥7-day washout).<br>**Type / Intensity:** Laboratory performance testing sequence after rinsing: Posner cueing task, maximal grip strength, countermovement jump (CMJ), and tee-based softball batting (5 sets × 5 swings; ≥3 min rest between sets). Batting performed at all-out effort while aiming to a target (10°; 10 m).	**Duration:** Diet standardized across trials by replicating pre-trial food intake; standard lunch at 12:00 on test day.<br>**Type:** Carbohydrate mouth rinse exposure (no ingestion) vs placebo; meals replicated between trials.	**Type:** Double-blind randomized crossover: 25 mL rinse for 20 s with either 6.4% maltodextrin (CMR) or mineral water placebo (PLA), spat out; rinsing performed before the test segments (including before each batting set).	**INTERVENTION (CMR)**<br>Launch angle (degrees) **mean±SD = 7.0 ± 2.6**<br>Launch angle variability / consistency (SD of launch angle) **mean±SD = 5.0 ± 0.9**<br>Average exit velocity (mph) **mean±SD = 97.4 ± 3.99**<br>Maximum exit velocity (mph) **mean±SD = 100.0 ± 4.0**<br>Posner cueing task – congruent RT (ms) **mean±SD = 288.0 ± 33.59**<br>Posner cueing task – incongruent RT (ms) **mean±SD = 353.4 ± 39.3**<br>Posner cueing task – correct rate (%) **mean±SD = 96.07 ± 2.66**<br>**PLACEBO (PLA)**<br>Launch angle (degrees) **mean±SD = 6.6 ± 2.5**<br>Launch angle variability /

							consistency (SD of launch angle) **mean±SD = 5.8 ± 1.5**<br>Average exit velocity (mph) **mean±SD = 97.5 ± 4.01**<br>Maximum exit velocity (mph) **mean±SD = 99.9 ± 4.8**<br>Posner cueing task – congruent RT (ms) **mean±SD = 288.6 ± 31.04**<br>Posner cueing task – incongruent RT (ms) **mean±SD = 348.8 ± 33.4**<br>Posner cueing task – correct rate (%) **mean±SD = 95.87 ± 3.10**<br>**CONTROL**<br>Not included (crossover design: CMR vs placebo only).
45	Ispoglou 2015	Design: RCT<br>Location: UK	Trained male cyclists (n=7 completed; n=9 initially recruited). Inclusion: ≥3 years cycling experience; ≥3 sessions/week, ~60 min/session. Mean ± SD: age 30 ± 6.7 y; body mass 82.8 ± 3.5 kg; BMI 24.0 ± 0.8 kg/m²; Wmax 347 ± 35 W; VO_2peak 58.7 ± 4.3 ml·kg⁻¹·min⁻¹.	**Duration:** ~1 hour (set amount of work designed to be completed in ~60 min)<br>**Type / Intensity:** Laboratory simulated cycling time trial on cycle ergometer; linear mode set using Wmax; workload anchored at ~75% Wmax (self-paced completion of fixed work).	**Duration:** 24 h pre-trial + ~3 h pre-exercise (postprandial)<br>**Type:** Replicated habitual "pre-race" diet using 24-h diet diary; last meal ~3 h before testing; water only permitted pre-trial (recorded and replicated); no fluid ingestion during the time trial.	**Type:** 0% CHO placebo vs 4%, 6%, 8% CHO electrolyte solutions; 25 ml per rinse, 5 s rinse then expectorate; administered pre- and post-warm-up and after each 12.5% of the time trial; no additional fluids allowed.	**INTERVENTION (CHO mouth rinse):**<br>**BASELINE**<br>Blood glucose (mmol·L⁻¹) mean±SD= **4%: 4.6±0.6 ǀ 6%: 4.6±1.3 ǀ 8%: 4.8±0.6**<br>Blood lactate (mmol·L⁻¹) mean±SD= **4%: 0.9±0.4 ǀ 6%: 0.8±0.2 ǀ 8%: 0.7±0.2**<br>**POST-INTERVENTION**<br>Time trial completion time (min) mean±SD= **4%: 62.8±4.0 ǀ 6%: 63.4±3.4 ǀ 8%: 63.0±4.0**<br>Mean power output (W) mean±SD= **4%: 248±28 ǀ 6%: 246±31 ǀ 8%: 247±33**<br>**PLACEBO (0% CHO mouth rinse):**<br>**BASELINE**<br>Blood glucose (mmol·L⁻¹) mean±SD= **4.5±0.5**<br>Blood lactate (mmol·L⁻¹) mean±SD= **1.0±0.3**<br>**POST-INTERVENTION**<br>Time trial completion time (min) mean±SD= **62.0±3.0**

							Mean power output (W)<br>mean±SD= **251±28**<br>**CONTROL:**<br>**No-control condition reported**
46	James 2017	Design: RCT<br>Location: UK	**Participants:** Competitive male cyclists (n=11; 12 recruited, 1 excluded). Endurance-trained; accustomed to ≥1 h cycling. Age 40 ± 8 y; body mass 77.6 ± 7.0 kg; height 1.79 ± 0.07 m; VO_2peak 58 ± 11 ml·kg⁻¹·min⁻¹.	**Duration:** ~1 h (fixed workload equivalent to 60 min at 75% Wpeak; ~844 ± 63 kJ)<br>**Type / Intensity:** Self-paced cycling time trial on an electronically braked cycle ergometer; workload set relative to **75% Wpeak** (high-intensity endurance).	**Duration:** Overnight fast prior to each trial; standardised/replicated diet and low-intensity activity before subsequent trials (no feeding intervention during the trial).<br>**Type: No carbohydrate ingestion** (fasted trials; diet replication and abstinence from alcohol/strenuous exercise pre-trial).	**Type:** 25 mL mouth rinse for 5 s, then expectorated, at start and every 12.5% of the TT: **7% maltodextrin**, **14% maltodextrin**, or **taste-matched placebo (0% CHO)**.	**INTERVENTION (CHO mouth rinse)**<br>**7% CHO:** Time to complete ~1 h cycling time trial **57.3 ± 4.5 min**<br>**14% CHO:** Time to complete ~1 h cycling time trial **57.4 ± 4.1 min**<br>**PLACEBO**<br>**PLA (0% CHO, taste-matched):** Time to complete ~1 h cycling time trial **59.5 ± 4.9 min**
47	Jeffers 2015	Design: RCT<br>Location: UK	Healthy adults, **male** cyclists/triathletes (**n = 9**); moderate–well trained; competing at regional/national level; experienced in time-trial testing.<br>Age: **33.2 ± 7.4 y**; Body mass: **76.7 ± 6.0 kg**; Height: **178.6 ± 4.6 cm**; VO_2max: **61.3 ± 5.3 mL·kg⁻¹·min⁻¹**	**Duration: ~60 min** total per trial (**45 min pre-load + 15 min TT**)<br>**Type / Intensity:**<br>**Pre-load:** cycling at **70% Wmax** (fixed work rate; hyperbolic mode) for **45 min**<br>**Time trial: 15 min** self-paced cycling TT ("as much work as possible"; linear mode where power increases with cadence)	**Duration: 24 h pre-trial diet replication** + **fasting ≤ 4 h** pre-visit (post-absorptive state); hydration standardised (**35 mL·kg⁻¹ water the day before**)<br>**Type:** dietary standardisation only (food diary recorded before Visit 2 and replicated before Visit 3; advised CHO-rich meal the evening before)	**Type:**<br>**CHO:** tasteless, colourless **6.4% maltodextrin** solution; **25 mL**, rinse **5 s**, then spit out<br>**PLA: water**, same rinse protocol<br>**Timing:** immediately before exercise and every **7.5 min** during the 45-min pre-load; immediately before and halfway through the 15-min TT	**INTERVENTION (CHO mouth-rinse)**<br>**15-min TT mean power output (W)**<br>**BASELINE:** N/A (TT outcome only)<br>**POST-INTERVENTION: 248 ± 23**<br>**MVC (N)**<br>**BASELINE: 505 ± 11**<br>**POST-INTERVENTION: 444 ± 63**<br>**Voluntary Activation (%)**<br>**BASELINE: 87 ± 6**<br>**POST-INTERVENTION: 83 ± 6**<br>**Qtw,pot (N)**<br>**BASELINE: 187 ± 29**<br>**POST-INTERVENTION: 158 ± 23**<br>**Mmax (mV)**<br>**BASELINE: 1.68 ± 1.33**<br>**POST-INTERVENTION: 1.37 ± 0.71**<br>**MEP Amplitude (mV)**<br>**BASELINE: 0.26 ± 0.11**<br>**POST-INTERVENTION: 0.12 ± 0.07**

							**MEP Latency (ms)**<br>**BASELINE: 38.1 ± 17.4**<br>**POST-INTERVENTION: 38.0 ± 18.4**<br>**PLACEBO (Water mouth-rinse)**<br>**15-min TT mean power output (W)**<br>**BASELINE:** N/A (TT outcome only)<br>**POST-INTERVENTION: 248 ± 39**<br>**MVC (N)**<br>**BASELINE: 511 ± 54**<br>**POST INTERVENTION: 407 ± 67**<br>**Voluntary Activation (%)**<br>**BASELINE: 89 ± 4**<br>**POST-INTERVENTION: 80 ± 8**<br>**Qtw,pot (N)**<br>**BASELINE: 190 ± 27**<br>**POST-INTERVENTION: 152 ± 23**<br>**Mmax (mV)**<br>**BASELINE: 2.49 ± 1.09**<br>**POST-INTERVENTION: 1.65 ± 1.36**<br>**MEP Amplitude (mV)**<br>**BASELINE: 0.31 ± 0.28**<br>**POST-INTERVENTION: 0.17 ± 0.20**<br>**MEP Latency (ms)**<br>**BASELINE: 27.3 ± 9.0**<br>**POST-INTERVENTION: 34.3 ± 14.0**<br>**CONTROL**<br>Not reported as a separate non-rinse control group (only **CHO vs Water placebo** crossover).
48	Jensen 2015	Design: RCT<br>Location: Canada	12 competitive male athletes (mean ± SD: age 26.7 ± 6.7 y; body mass 78.2 ± 5.5 kg; height 184.9 ± 6.1 cm). Sport background: 9 rowers, 1 cyclist, 1 runner, 1 volleyball	**Duration:** Two acute laboratory trials (double-blind crossover) separated by 4.6 ± 2.2 days; each trial performed in the morning (7–10 a.m.) after an overnight fast.	**Duration:** Acute, single exposure per trial: mouth rinse for 10 s immediately post-fatigue (no ingestion).<br>**Type:** Carbohydrate mouth rinse using 8% maltodextrin	**Type:** 10-s mouth rinse administered immediately after the fatiguing contraction and before the post-fatigue MVC set; solution expelled (double-blind,	**INTERVENTION (CHO mouth rinse – WASH)**<br>**Peak torque (Nm)**<br>**BASELINE (PRE):** 364.0 ± 12.6<br>**POST-INTERVENTION (POST-**

			player. Regularly active (training 3–4 times/week); varsity to national-level experience.	**Type / Intensity:** Standardized 15-min submax cycling warm-up (70–80 rpm; 2%, 3%, 4% body weight). Then 3 × 5-s isometric knee extension MVCs (baseline), followed by a fatiguing isometric contraction at 50% MVC until volitional exhaustion (torque drop >10% for >5 s). Post-fatigue: 3 × 5-s MVCs (10 s rest between).	(taste-matched with 0.2% sucralose); placebo was non-caloric artificial sweetener solution (also taste-matched).	randomized crossover: CHO rinse vs placebo).	**fatigue MVC decrease vs baseline):**<br>MVC1: −31.7 ± 9.9<br>MVC2: −30.3 ± 9.5<br>MVC3: −31.6 ± 7.4<br>**3-s Average torque (Nm)**<br>**BASELINE (PRE):** 344.2 ± 11.2<br>**POST-INTERVENTION (POST-fatigue MVC decrease vs baseline):**<br>MVC1: −25.9 ± 10.1<br>MVC2: −27.2 ± 8.8<br>MVC3: −28.2 ± 7.3<br>**PLACEBO (PLA)**<br>**Peak torque (Nm)**<br>**BASELINE (PRE):** 351.5 ± 10.9<br>**POST-INTERVENTION (POST-fatigue MVC decrease vs baseline):**<br>MVC1: −38.7 ± 9.8<br>MVC2: −32.7 ± 8.3<br>MVC3: −33.1 ± 7.7<br>**3-s Average torque (Nm)**<br>**BASELINE (PRE):** 334.1 ± 10.2<br>**POST-INTERVENTION (POST-fatigue MVC decrease vs baseline):**<br>MVC1: −36.0 ± 11.5<br>MVC2: −32.5 ± 8.5<br>MVC3: −30.6 ± 8.2<br>**CONTROL**<br>**Control group: Not included** (this study used only **WASH vs PLA** in a double-blind crossover design).
49	Karayigit 2022	Design: RCT<br>Location: Turkey	Healthy **resistance-trained males** (n = **16**);<br>Age **25 ± 3 years**; height **182 ± 6 cm**; body mass **86 ± 3 kg**; body fat **16 ± 3%**; bench press **1-RM 106 ± 16 kg**.	**Duration:**<br>Acute experimental protocol; **four separate laboratory visits,** separated by **72 h;** during each visit **3 sets** of bench press performed to failure with **3-min passive rest** between sets.	**Duration:**<br>**Overnight fasting (~12 h)** prior to each experimental session.<br><br>**Type:**	**Type:**<br>**Carbohydrate mouth rinse (CMR):**<br>25 mL of **6% maltodextrin solution**, sweetened with sucralose; rinsed for **10 s**, **three times** (once between each set).	**INTERVENTION (CMR)**<br>**1-RM (Bench Press)** mean±SD = **106 ± 16 kg**<br>**Strength Endurance (Bench Press repetitions to failure; mean of 3 sets)**<br>**40% 1-RM:** mean±SD = **18.33 ± NR**

			**Participants:**<br>Resistance-trained individuals with **≥5 years training experience**, training **≥4 sessions/week**; age **19–30 years**; free from neuromuscular or musculoskeletal disorders; capable of bench pressing **≥100% body mass**.	**Type / Intensity:**<br>**Resistance exercise (bench press)** performed at **40% 1-RM (low intensity) or 80% 1-RM (high intensity)**<br>Repetition tempo standardized **(2 s eccentric + 2 s concentric**); exercise performed to **volitional failure.**	Dietary standardization only; participants replicated their **24-h pre-test diet** across all sessions; no caloric intake during testing.	**Placebo:**<br>Sweetened water matched for taste and appearance.	**80% 1-RM:** mean±SD = **5.64 ± NR**<br>**Heart Rate (beats/min) mean±SD**<br>**CMR40%:**<br>Pre-test **64.37 ± 6.42**<br>1st set **126.37 ± 20.74**<br>2nd set **129.56 ± 20.41**<br>3rd set **133.31 ± 23.36**<br>**CMR80%:**<br>Pre-test **63.18 ± 6.18**<br>1st set **133.93 ± 21.54**<br>2nd set **135.00 ± 18.30**<br>3rd set **137.68 ± 20.44**<br>**RPE (6–20) mean±SD**<br>**CMR40%:**<br>1st set **14.06 ± 2.83**<br>2nd set **16.12 ± 1.66**<br>3rd set **17.62 ± 1.74**<br>**CMR80%:**<br>1st set **14.50 ± 3.42**<br>2nd set **15.75 ± 2.64**<br>3rd set **16.87 ± 2.75**<br>**Felt Arousal (1–6) mean±SD**<br>**CMR40%:**<br>1st set **5.00 ± 0.81**<br>2nd set **4.06 ± 1.06**<br>3rd set **3.87 ± 1.25**<br>**CMR80%:**<br>1st set **4.62 ± 0.95**<br>2nd set **4.12 ± 0.95**<br>3rd set **3.87 ± 1.14**<br>**Glucose (mg/dL) mean±SD**<br>**CMR40%:** Pre-test **78.68 ± 15.75** | Post-test **88.43 ± 6.93**<br>**CMR80%:** Pre-test **88.06 ± 13.12** | Post-test **86.87 ± 15.29**<br><br>**PLACEBO (PLA)**<br>**1-RM (Bench Press)** mean±SD = **106 ± 16 kg**

							**Strength Endurance (Bench Press repetitions to failure; mean of 3 sets)**<br>**40% 1-RM:** mean±SD = **18.75 ± NR**<br>**80% 1-RM:** mean±SD = **4.97 ± NR**<br>**Heart Rate (beats/min) mean±SD**<br>**PLA40%:**<br>Pre-test **64.93 ± 7.91**<br>1st set **129.18 ± 20.66**<br>2nd set **129.62 ± 22.10**<br>3rd set **133.00 ± 24.58**<br>**PLA80%:**<br>Pre-test **64.62 ± 6.50**<br>1st set **128.62 ± 20.33**<br>2nd set **132.87 ± 20.47**<br>3rd set **135.00 ± 21.25**<br>**RPE (6–20) mean±SD**<br>**PLA40%:**<br>1st set **14.12 ± 3.15**<br>2nd set **16.68 ± 1.95**<br>3rd set **17.43 ± 1.86**<br>**PLA80%:**<br>1st set **14.43 ± 2.63**<br>2nd set **16.06 ± 1.80**<br>3rd set **17.00 ± 1.59**<br>**Felt Arousal (1–6) mean±SD**<br>**PLA40%:**<br>1st set **4.31 ± 1.07**<br>2nd set **4.00 ± 1.09**<br>3rd set **3.56 ± 1.20**<br>**PLA80%:**<br>1st set **4.50 ± 1.31**<br>2nd set **4.00 ± 1.15**<br>3rd set **3.93 ± 1.12**<br>**Glucose (mg/dL) mean±SD**<br>**PLA40%:** Pre-test **81.18 ± 14.00** | Post-test **88.37 ± 8.36**<br>**PLA80%:** Pre-test **82.75 ± 10.68** | Post-test **88.56 ± 7.56**<br>**CONTROL**

								**NR (no control / no-rinse condition included)**
50	Karayigit 2021	Design: RCT<br>Location: Turkey	**Participants:** Sixteen healthy, non-smoking resistance-trained females (age: 20 ± 1 years; height: 167 ± 3 cm; body mass: 67 ± 4 kg; BMI: 17 ± 2 kg/m²; resistance training experience: 2 ± 1 years)	**Duration:** Acute, single-session testing per condition; 4 experimental sessions total, performed on separate days (48 h apart)<br>**Type / Intensity:** Bench press **1-RM strength test** + bench press **muscular endurance test at 40% of 1-RM (repetitions to failure)**; movement tempo controlled (2 s eccentric + 2 s concentric)	**Duration:** Acute (each session); morning testing (08:00–10:00) after **10 h overnight fast**<br>**Type:** No nutrition/supplement ingestion; dietary control only (24-h dietary intake recorded and replicated before each condition)	**Type: 25 mL** mouth rinse, swished for **10 s** and **spat out** (no ingestion): **6% CHO (low), 12% CHO (moderate), 18% CHO (high)** maltodextrin solutions, plus **water placebo**; administered immediately prior to each **1-RM** test and prior to the **muscular endurance** test	**INTERVENTION (CHO mouth rinse: 6%, 12%, 18%)**<br><br>**PLACEBO (Water mouth rinse)**
51	Karayigit 2021	Design: RCT<br>Location: Iran	Healthy Adults, **Male** (n=13); Age 24 ± 3 years; Height 184 ± 7 cm ; Weight 84 ± 8 kg ;<br>**Female** (n=14); Age 21±1 years; Height 170 ± 5 cm ; Weight 68 ± 6 kg ;<br><br>**Participants** had previous experience of at least 3 times per week of resistance training for the previous 1year<br><br>*A double-blind, randomised, counter-balanced and crossover research design	**Duration**: 6 sessions<br>**Type**: Strength (1-RM) and muscular endurance (40% of 1-RM) test protocols; 10 min warm-up; Bench press and back full squat; 3 sets; 2min passive rest between sets<br>**Days/week**: separated by 48–96 h to allow recovery<br>**Intensity**: max for 1RM strength; 40% of 1-RM for endurance<br><br>Participants were asked to avoid physical exercise in the 12 h leading up to each visit	**Duration**: 6 sessions<br>**Type**: 12 h overnight fasting; Participants were asked to avoid CAF and alcohol intake in the 12 h leading up to each visit	**Type**: 25 ml solution; 10 sec mouth rinse; All solutions were flavored with 300 mg of sucralose and were similar in appearance; Each solution was administered 8 times once a min during 8 min prior to second CF test and immediately before each attempt in the 1-RM test and at each min (2 times in total) between sets in the muscular endurance test<br>**Intervention**: mouth rinsing of 6% carbohydrate (CHO)<br>**Placebo**: Water	<u>Strength (1-RM)</u><br>bench press<br>squat<br><br><u>Muscular endurance (40% of 1-RM)</u><br>bench press<br>squat
52	Karuk 2022	Design: RCT<br>Location: Turkey	Eight highly trained healthy male athletes. Age 22.3±4.2 years; Height 181±6 cm	2 trials: jump heights performed during 30 s	The participants were required to refrain from vigorous physical activity at least 24 h prior to the testing session and continue to consume the same diet and not to change their sleep patterns throughout the study period.	CHO (6.4% maltodextrin solution), CAF (1.2% caffeine solution), CHO + CAF (6.4%+1.2%) combination, and PLA (non-caloric sweetener: saccharin)	No positive effect of CHO mouth rinse on jumping performances in trained male athletes

53	Khong 2020	Design: RCT<br>Location: Malaysia	Healthy Adults, Male (n=10); Age 22±1 years; Weight: 65.3±12.4 kg; Height: 164.5±7.5 cm;<br>**Participants** exercised for at least 30 minutes 3 days per week.<br>*a randomized cross-over study	**Duration**: 3 trials<br>**Type**: 30-minute cycling exercise;<br>**Days/week**: a 7-day washout period<br>**Intensity**: 70% VO2max (participants underwent a VO2max test to determine their maximum aerobic capacity and cycling intensity for the trial)<br>*They refrain from intense exercise for 24 hours prior to the trials.	**Duration**: three trials<br>**Type**: They refrained from intense caffeine, and alcohol for 24 hours, and fasted overnight prior to the trials;	**Type**: The designated mouth rinse was given immediately before the cycling exercise, and at every 10 min 10th, 20th, and 30th min during the trial. The solution was rinsed for 5 sec<br>**Intervention:** 6% glucose<br>**Placebo**: 6% NaCl<br>**Control**: water	MVC<br>**Intervention** mean±SD<br>Sugar Pre: 131.5 ± 41.5<br>Sugar Post: 109.5 ± 37.1<br>**Control** mean±SD<br>Water Pre: 128.1 ± 53.1<br>Water Post: 83.2 ± 39
54	Krings 2017	Design: RCT<br>Location: USA	Healthy Adults, Male (n=14), Age 21.7 ± 1.8 years, BM 82.3 ± 12.3 kg; Height: 177.3 ± 6.4 cm<br>**Participants** Recreationally active males<br>*a double-blinded, placebo controlled, and randomized cross-over design	**Duration**: 5 sessions,<br>**Type**: Sprints on a cycle ergometer; The protocol lasted a total of 22 min and 15 sec; 5 min warm-up, subjects immediately began a series of five maximal 15-s sprints interspersed with 4 min of active recovery at 50 W; At the beginning of each sprint, 0.075 kg·kg-1 body mass of resistance was magnetically added<br>**Days/week**: Each trial was separated by at least 7 days<br>**Intensity**: acute repeat maximal sprint performance	**Duration**: 5 sessions,<br>**Type**: At least a ten hour fast	**Type**: Subjects received a total of six beverages; 50 mL solution; were administered immediately before the 5-min warm-up and 45 s before each sprint; 10 sec mouth rinse; Subjects mouth rinsed a total of 300 mL during each experimental session<br>**Intervention**: containing 10% CHO solution<br>**Placebo**: sugar free similarly flavored placebo solution	Mean power output W<br>**Intervention** mean±SD<br>645.8 ± 99.7<br>**Placebo** mean±SD<br>656.3 ± 104.1<br>Peak power output W<br>**Intervention** mean±SD<br>719.6 ± 159.2<br>**Placebo** mean±SD<br>721.3 ± 154.8<br>Total work (J)<br>**Intervention** mean±SD<br>9477.5 ± 1684.9<br>**Placebo** mean±SD<br>9671.2 ± 1808.8<br>Heart rate (bpm)<br>**Intervention** mean±SD<br>167.7 ± 11.8<br>**Placebo** mean±SD<br>167.0 ± 13.4<br>RPE (Borg 1–10)<br>**Intervention** mean±SD<br>6.2 ± 2.2<br>**Placebo** mean±SD<br>5.9 ± 2.0
55	Kulaksız 2016	Design: RCT<br>Location: Turkey	Healthy Adults, Male (n=9); Age: 24 ± 2 years; BM: 80 ± 11 kg;	**Duration:** Five sessions<br>**Type:** Incremental cycling protocol in a braked cycle	**Duration:** Five sessions<br>**Type:** Overnight fast 10 hours before each experimental trial	**Type:** Mouth Rinse Protocol<br>Volume: 50 mL per rinse; 5 sec mouth rinse	CHO mouth rinsing after an overnight fast did not affect the

			BMI: 25.2 ± 2.7 kg·m⁻²; Body fat: 21.8 ± 3.3%<br><br>**Participants** Recreationally active	ergometer; warm up for 5 min at 60 W; Participants cycled a 20 km distance as fast as possible with 2.5 kg (150 W) constant resistance.<br><br>**Days/week:** Cycling trials were separated by at least 48 h<br>**Intensity:** Self-paced effort, performed "as fast as possible"<br><br><br>*Abstinence from strenuous exercise for the 24 h preceding an experimental trial.	24 h pre-trial diet replication across all conditions; Abstinence from caffeine and alcohol for 24 h pre-trial	**Intervention:** Carbohydrate (maltodextrin) Solutions at 3%, 6%, and 12%; Frequency: Every 2.5 km during the 20 km time trial<br>**Placebo:** 0% maltodextrin, artificially sweetened; Frequency: Every 2.5 km during the 20 km time trial	physiological responses and performance during a 20 km cycling time trial
56	Lane 2013	Design: RCT<br>Location: Australia	Healthy Adults, Malee (n=12); Age 28 ± 5 years; BM: 74±5.1kg;<br><br>**Participants** Competitive endurance trained cyclists/triathletes; 358 ± 61 km/week training; >3 years endurance training history	**Duration:** 4 experimental trials<br>**Type:** 60-min simulated cycling; 10 min self-selected warm up, 60-min time trial,<br>**Days/week:** The experimental trials that were each separated by 7 days<br>**Intensity:** to complete as much work as possible within the 60 min (i.e., maintain their highest sustainable average power output)	**Duration:** 4 experimental trials<br>**Type:** Two trials were commenced 2 h after a meal that contained 2.5 g·kg−1 body mass of CHO (FED) and 2 after an overnight fast (FST); High-carbohydrate pre-exercise meal providing 2.5 g CHO·kg⁻¹ body mass (mixed-GI foods: cereal, milk, fruit, juice); no meal in fasted (FST) trials.<br>Acute (pre-exercise) — 2 hours before the time trial, following a 9–10 h overnight fast (for FED trials only)	**Type:** 20 mL rinsed for 10 sec and expectorated, administered at baseline and every 12.5% of the 60-min time trial.<br>**Intervention:** Carbohydrate mouth rinse; For the CHO trials, the bolus comprised of a 10% CHO- containing maltodextrin solution that was prepared by adding maltodextrin (100 g·L–1) to the noncaloric stock solution<br>**Placebo:** A commercially available noncaloric, artificially sweetened, and flavored concentrate that was diluted with water	**INTERVENTION**<br>**mean power output**<br><u>Power W</u><br>**mean power output**<br>FED+CHO mean±SE 286 ± 6<br>FST+CHO mean±SE 282 ± 6
57	Luden 2016	Design: RCT<br>Location: USA	Healthy Adults, Male (n=8); Age 24 ± 6 years; Weight, 74 ± 7 kg; Height, 176 ± 5 cm<br><br><br>**Participants** Cyclists; All subjects performed ≥3 days of cycling per week for ≥2	**Duration**: 4 sessions<br>**Type**: Cycle ergometer; 120 min of constant-load cycling 55%Wmax(190±22W) ; A simulated 30 km time trial (TT) (~57 min), 15 min of rest that included peak isometric force testing, 10 min of constant-load	**Duration**: 4 sessions<br>**Type**: Two hours after consumption of a standardized breakfast prior to each trial (500 kcal; 90–100 g carbohydrate; 8–12 g protein, and 4–8 g of fat);	**Type**: Subjects received 25 mL of each respective mouth Rinse; Three time points during the exercise trial: minutes 5:00 and 14:30 of the rest phase and at minute 8:00 of the 10 min constant-load segment. Subjects swirled each rinse in their	<u>**Constant-Load—55% Wmax**</u><br><u>Heart Rate (bpm)</u><br>**Intervention** mean±SD<br>156 ± 13<br>**Placebo** mean±SD<br>150 ± 13<br>**Protein** mean±SD<br>152 ± 60

			months prior to study enrollment<br><br>*a counterbalanced, double-blinded fashion	cycling (3 min at 35% Wmax and 7 min at 55% Wmax), and a computer simulated 2 km time trial (~4 min).<br>**Days/week**: Trials separated by seven to 10 days each<br>**Intensity**: Maximal 55%Wma<br><br>Subjects were instructed to refrain from heavy exercise for 48 h preceding each treatment trial	Subjects were also instructed to abstain from alcohol and caffeine for 24 and 12 h prior to each trial, respectively.	mouth for 5 s; The MR treatments contained 100 mL of deionized water (PLA) with the addition of either 6.4 g of maltodextrin or 6.4 g of hydrolyzed whey protein isolate. Commercially available stevia was used to minimize differences in taste and smell	<u>RPE</u><br>**Intervention** mean±SD<br>14 ± 20<br>**Placebo** mean±SD<br>14 ± 20<br>**Protein** mean±SD<br>14 ± 3<br><u>MVC (N)</u><br>**Intervention** mean±SD<br>**Pre** 445 ± 61 / **Post** 470 ± 70<br>**Placebo** mean±SD<br>**Pre** 456 ± 108 / **Post** 490 ± 106<br>**Protein** mean±SD<br>**Pre** 466 ± 84 / **Post** 475 ± 75<br><u>2 km TT Finishing Time (s)</u><br>**Intervention** mean±SD<br>192.4 ± 8.2<br>**Placebo** mean±SD<br>200.1 ± 10.8<br>**Protein** mean±SD<br>199.9 ± 18.4
58	Mazman 2024	Design: RCT<br>Location: Türkiye	20 healthy male football players (Regional Amateur League), age 20–25 years (mean 22.65 ± 1.90); height 180.15 ± 6.00 cm; body weight 73.95 ± 7.48 kg; BMI 22.74 ± 1.47 kg/m²; body fat 11.95 ± 1.76%; fat-free mass 64.81 ± 5.63 kg; football experience 10.50 ± 5.23 years; weekly training 11.30 ± 3.69 hours.	**Duration:** Single testing session (Yo-Yo Intermittent Recovery test with standardized 15-min warm-up).<br>**Type / Intensity:** Yo-Yo Intermittent Recovery Test (Level 1): progressive 2×20 m shuttle runs with 10-s active recovery between bouts; intensity increases stepwise until exhaustion (terminated when participant stops or fails twice to reach the line).<br>at three standardized time points: **before the warm-up**, **immediately before the Yo-Yo test**, and **midway through the test (after completion of the 1000-m stage)**. Blood **lactate** and **glucose** levels were measured	**Duration:** 1 week pre-test dietary monitoring + standardized diet program; 2-hour fast before testing.<br>**Type:** Individualized standardized nutrition plan prepared by a dietitian (targeting ~55–60% energy from carbohydrate, <30% fat, protein 1.5–2.0 g/kg/day) based on food records; dietary intake analyzed with BeBIS.	**Type:** 15% carbohydrate solution (CHO) vs placebo (sweetener-water). CHO prepared as 75 g dextrose in 500 mL water; rinse volume 50 mL for 5 s, performed 3 times (before warm-up, before Yo-Yo test, and mid-test after 1000 m). Placebo matched for taste/appearance.	**INTERVENTION (CHO mouth rinse, 15% dextrose)**<br>**Yo-Yo test distance (m):** mean±SD = **2866.00 ± 695.55** (Median **3000**; Min–Max **1500–3640**)<br>**VO_2max (mL·kg⁻¹·min⁻¹):** mean±SD = **60.51 ± 5.85** (Median **61.77**; Min–Max **49.00–66.98**)<br>**Blood Lactate (mmol/L)**<br>**BASELINE:** mean±SD = **2.55 ± 1.47** (Median **2.00**; Min–Max **1.40–6.00**)<br>**POST-INTERVENTION:** mean±SD = **5.38 ± 1.87** (Median **5.85**; Min–Max **2.20–7.60**)<br>**Blood Glucose (mg/dL)**<br>**BASELINE:** mean±SD = **93.80 ± 10.02** (Median **92**; Min–Max **81–113**)

				**before and after** the exercise session to assess physiological responses to the intervention.			**POST-INTERVENTION:** mean±SD = **98.50 ± 8.30** (Median **95.50**; Min–Max **89–115**)
59	Muhamad 2020	Design: RCT<br>Location: Malaysia**.**	Healthy adult **male recreational runners** *(n = 10)*; **age 18–26 years**; **non-smokers**; recruited from **Universiti Sains Malaysia (USM)**; screened with **PAR-Q**; **overnight fast** before each trial (water allowed).	**Duration:** Each trial = **1 h** steady-state run + **15 min** time trial (**75 min total**)<br><br>**Type / Intensity: Treadmill running** at **75% HRmax** for 60 min, immediately followed by a **15-min self-paced time trial** (distance recorded).	**Duration: Overnight fast** (from 11:00 p.m.); trials started **8:30 a.m.** (water permitted)<br>**Type: No carbohydrate ingestion** during trials (the carbohydrate exposure was via mouth rinse only). expectorate	**Type:** Three conditions (randomised, cross-over):<br>**CHO mouth rinse: 25 mL** every **15 min**, rinse **10 s**, **expectorate** (glucose solution **64 g/L**)<br>**Placebo (PLA) mouth rinse: 25 mL** every **15 min**, rinse **10 s**, **expectorate** (stevia **17 g/L**, non-caloric; matched taste/colour)<br>**Control (CON): No mouth rinse**	**INTERVENTION (CHO mouth rinse)**<br>**HR (beats/min)**<br>**BASELINE:** 75.3 ± 3.4<br>**POST-INTERVENTION:** 162.7 ± 1.5<br>**RPE (6–20 scale)**<br>**BASELINE:** 6.0 ± 0.0<br>**POST-INTERVENTION:** 15.2 ± 1.2<br>**Mood (BRUMS) – Pre vs Post**<br>**Vigour:** 57.2 ± 13.1 → 57.2 ± 7.1<br>**Anger:** 44.4 ± 1.2 → 44.7 ± 2.2<br>**Confusion:** 44.8 ± 4.3 → 48.0 ± 9.1<br>**Fatigue:** 48.8 ± 6.2 → 55.7 ± 9.2<br>**Tension:** 49.1 ± 9.5 → 49.4 ± 10.6<br>**Depression:** 44.5 ± 2.5 → 47.5 ± 6.9<br>**Running performance (15-min time trial distance, km) POST-INTERVENTION:** 2.34 km<br>**PLACEBO (PLA mouth rinse)**<br>**HR (beats/min)**<br>**BASELINE:** 78.5 ± 4.4<br>**POST-INTERVENTION:** 165.3 ±2.4<br>**RPE (6–20 scale)**<br>**BASELINE:** 6.0 ± 0.0<br>**POST-INTERVENTION:** 15.8 ± 2.7<br>**Mood (BRUMS) – Pre vs Post**<br>**Vigour:** 60.6 ± 11.3 → 53.4 ± 7.6<br>**Anger:** 44.8 ± 1.6 → 45.8 ± 4.4<br>**Confusion:** 47.2 ± 4.2 → 48.6 ± 10.9<br>**Fatigue:** 48.4 ± 7.8 → 56.9 ± 9.9<br>**Tension:** 54.8 ± 14.0 → 51.4 ± 13.8<br>**Depression:** 44.5 ± 2.5 → 51.3 ± 14.8

							**Running performance (15-min time trial distance, km)**<br>**POST-INTERVENTION:** 2.33 km<br>**Salivary lysozyme (µg/mL)**<br>**Figure only (graph); no exact table values provided**.<br>**CONTROL (CON – no mouth rinse)**<br>**HR (beats/min)**<br>**BASELINE:** 76.1 ± 4.6<br>**POST-INTERVENTION:** 163.4 ± 3.7<br>**RPE (6–20 scale)**<br>**BASELINE:** 6.0 ± 0.0<br>**POST-INTERVENTION:** 14.5 ± 5.1<br>**Mood (BRUMS) – Pre vs Post**<br>**Vigour:** 57.6 ± 11.3 → 57.0 ± 10.2<br>**Anger:** 45.1 ± 2.4 → 47.4 ± 8.5<br>**Confusion:** 44.7 ± 3.3 → 47.7 ± 13.0<br>**Fatigue:** 50.2 ± 9.6 → 55.9 ± 10.6<br>**Tension:** 46.8 ± 6.3 → 48.3 ± 9.9<br>**Depression:** 44.8 ± 8.9 → 50.1 ± 11.9<br>**Running performance (15-min time trial distance, km)**<br>**POST-INTERVENTION:** 2.23 km
60	Murray 2018	Design: RCT<br>Location: USA	Healthy, trained, competitive **male cyclists/triathletes** (final **n = 8**; mean age **24 ± 2 y**; **VO_2max 64.5 ± 2.2 ml·kg⁻¹·min⁻¹**). Regular endurance training (≥2 sessions/week). Two initial volunteers excluded (injury or dietary non-compliance).	**Duration:** Single **40 km** cycling time-trial (≈ **67–68 min**)<br>**Type / Intensity: Self-paced cycling time-trial** on an electromagnetically braked cycle ergometer; performed "as fast as possible" (high-intensity competitive effort).	**Duration: ~5 h pre-test** (before each experimental visit)<br>**Type:** Standardized pre-trial meal (~**800 kcal**; **45% CHO, 34% fat, 20% protein**), same meal and timing before each trial (to minimize glycogen differences).	**Type: CHO rinse vs Placebo rinse**<br>**CHO: 6.4% glucose (dextrose)** solution, **25 mL**, rinsed **10 s** then expectorated, **pre-exercise + every 5 km**<br>**PLA: Water** solution matched for sweetness/color/flavor (non-caloric sweetener + flavoring), same rinse schedule	**INTERVENTION (CHO mouth rinse)**<br>**40 km time-trial performance time: 67.1 ± 1.1 min**<br>**Mean power output: 244 ± 10 W**<br>**PLACEBO (PLA mouth rinse)**<br>**40 km time-trial performance time: 67.9 ± 1.0 min**<br>**Mean power output: 237 ± 8 W**<br>**BASELINE (reported numbers)**<br>**Fasting plasma insulin: ~5.9 ± 0.7 µU·mL⁻¹** *(mean ± SE)*<br>**POST-INTERVENTION**

							**Plasma insulin (first 25 km):** reported as **no significant difference** between CHO and PLA (p = 0.638), **Plasma glucose (0–40 km):** reported as **no significant difference** between CHO and PLA (p = 0.801)
61	Nehme 2022	Design: RCT Location: Brazil	12 male athletes, age 18.9 ± 0.5 y; height 181 ± 0.06 cm; body mass 72.7 ± 5.3 kg	3 trials, Yo-Yo IR 1 test protocol	The participants performed the Yo-Yo test after an overnight fast.	Single mouth rinse with CHO (8% maltodextrin or eight mouth rinses with CHO (8-CHO). Placebo (commercial noncaloric juice)	CHO mouth rinsing is not an effective ergogenic strategy for intermittent exercise performance irrespective of the number of rinses
62	Nyman 2022	Design: RCT Location: Canada	12 skilled male ice hockey players (n=12); age 22.6 ± 3.4 y; height 178.9 ± 4.7 cm; body mass 84.0 ± 6.5 kg; skill level AA U18 to collegiate/major junior	**Duration:** ~90 min total (10-min warm-up + 3 × 20-min regulation periods + 1 × 12-min overtime; 2-min intermissions; repeated 2-min shift/rest intervals). **Type / Intensity:** On-ice small-sided 3-on-3 scrimmage (intermittent, high-intensity skating with repeated sprints, accelerations, and decelerations; internal intensity monitored via HR, TRIMP, and RPE).	**Duration:** N/A (no dietary manipulation; usual habits maintained; prescrimmage caffeine and CHO intake not restricted). **Type:** None (hydration maintained with water; no CHO ingestion as an intervention).	**Type:** Carbohydrate (CHO) mouth-rinse vs placebo (PLA) mouth-rinse; 25 mL per rinse, 10 s swish, expectorated; ~every 10 min; total 7 rinses (Gatorade 6% CHO electrolyte solution vs Gatorade Zero taste-/electrolyte-matched placebo).	**INTERVENTION (CHO mouth rinse)** **BASELINE (Regulation – Period 1):** Peak speed (km·h⁻¹) mean±SD = **24.3 ± 1.3** Average speed (km·h⁻¹) mean±SD = **12.6 ± 0.9** Sprints (n) mean±SD = **2.7 ± 1.9** Decelerations (n) mean±SD = **19.3 ± 4.2** **POST-INTERVENTION (Regulation – Period 3):** Peak speed (km·h⁻¹) mean±SD = **24.4 ± 1.6** Average speed (km·h⁻¹) mean±SD = **12.3 ± 0.7** Sprints (n) mean±SD = **2.4 ± 1.6** Decelerations (n) mean±SD = **15.7 ± 3.8** **POST-INTERVENTION (Overtime – OT):** High-intensity distance (m) mean±SD = **224 ± 77** Slow-speed distance (m) mean±SD = **300 ± 33** Peak speed (km·h⁻¹) mean±SD = **24.6 ± 1.6** Sprints (n) mean±SD = **1.9 ± 1.2**

							Peak HR (bpm) mean±SD = **183.3 ± 9.1**<br>Average HR (bpm) mean±SD = **153.7 ± 11.8**<br>TRIMP (AU) mean±SD = **24.9 ± 8.5**<br>RPE (Borg-10) mean±SD = **7.7 ± 0.9**<br>**PLACEBO (PLA mouth rinse)**<br>**BASELINE (Regulation – Period 1):**<br>Peak speed (km·h⁻¹) mean±SD = **24.5 ± 1.2**<br>Average speed (km·h⁻¹) mean±SD = **12.8 ± 0.7**<br>Sprints (n) mean±SD = **1.9 ± 1.1**<br>Decelerations (n) mean±SD = **19.9 ± 5.9**<br>**POST-INTERVENTION (Regulation – Period 3):**<br>Peak speed (km·h⁻¹) mean±SD = **24.6 ± 1.7**<br>Average speed (km·h⁻¹) mean±SD = **12.6 ± 1.0**<br>Sprints (n) mean±SD = **2.3 ± 1.2**<br>Decelerations (n) mean±SD = **15.8 ± 5.2**<br>**POST-INTERVENTION (Overtime – OT):**<br>High-intensity distance (m) mean±SD = **185 ± 66**<br>Slow-speed distance (m) mean±SD = **336 ± 47**<br>Peak speed (km·h⁻¹) mean±SD = **23.7 ± 1.3**<br>Sprints (n) mean±SD = **1.2 ± 0.9**<br>Peak HR (bpm) mean±SD = **185.7 ± 9.0**<br>Average HR (bpm) mean±SD = **155.2 ± 11.3**<br>TRIMP (AU) mean±SD = **26.4 ± 8.2**<br>RPE (Borg-10) mean±SD = **7.5 ± 0.9**

63	Oziukan Sahin 2024	Design: RCT<br>Location: Turkey	16 male karate athletes 18-30 years old	Repeated sprint test (6sec sprint ×10 repetitions, with 30sec rest) on a bicycle ergometer		10 seconds mouth rinsing of CHO (6.4% maltodextrin), CAF (1.2%), and placebo (water) solutions	CHO mouth rinse does not improve repeated sprint performance
64	Painelli 2011	Design: RCT<br>Location: Brazil	Healthy Adults, Male (n=12); Age 24 ± 3 years; Weight 79 ± 8 kg; height 178.9 ± 7.0 cm<br><br>**Participants** Recreationally strength-trained strength-training experience 1–2 years	**Duration:** Eight sessions<br>**Type:** Strength Bench press on a Smith machine; **Maximum strength evaluation**, 5 min warm-up, stretching exercise, warm-up set of eight repetitions at approximately 50% of the estimated 1-RM and, one set of one set of three repetitions at 70% of the estimated 1-RM and single repetitions of progressively heavier loads, until failure;<br>**Strength endurance evaluation,** six sets until failure at 70% of the 1-RM, 2-min rest interval between sets<br>**Days/week:** Sessions were conducted on different days 96 h apart<br>**Intensity:** maximum strength test and a strength endurance test until failure at 70% of 1-RM<br>*Subjects were instructed to refrain from any sort of exhaustive exercise during the experimental protocol	**Duration:** Eight sessions<br>**Type:** 8-hour overnight fast before each test session<br>Food intake standardized the night before each test session	**Type:** Each solution was administered immediately before each attempt in the 1-RM test and immediately before each set in the strength endurance test; 25 mL of solution; rinsed for 10–15 sec;<br>**Intervention:** Carbohydrate (CHO) 25 mL of a 6.4% dextrose solution<br>**Placebo:** Solution with non-caloric sweetener (aspartame), matched for taste and appearance<br>**Control:** no mouth rinse	**INTERVENTION**<br>**1-PM** mean±SD=<br>**Strength Endurance** mean±SD=<br>**PLACEBO**<br>**1-PM** mean±SD=<br>**Strength Endurance** mean±SD=<br>**CONTROL**<br>**1-PM** mean±SD=<br>**Strength Endurance** mean±SD=
65	Pereira 2020	Design: RCT<br>Location: Brazil	Healthy Adults, Female (n=29); Age 24±4.5 years; BM 60±7.9 kg; Height 161.0±6.0 cm<br><br>**Participants** Physical activity women were engaged in resistance training for at least 1 year,	**Duration**: 6 sessions<br>**Type**: Resistance training in following exercises: squat, leg press, bench press, shoulder press, and row; In subsequent visits, subjects performed all four sessions of strength training; 3 sets; The rest interval between sets was of 1 min; Each repetition was completed in 4 s,	**Duration**: 6 sessions<br>**Type**: The tests were performed following an overnight fast of 8 h'<br>Participants were encouraged to keep their meals as usual throughout the experimental period, and food intake was standardized in the morning	**Type**: subjects ingested a cellulose capsule 60 min before the strength training session and mouth rinsing for 10 s<br>**Intervention**: CHO mouth rinse; 6 grams of maltodextrin and 100 mL before the session<br>**Placebo:** In the PLA session; 100 mL of a placebo solution before the session.	CHO mouth rinse can improve performance in resistance training.

			from 3 to 5 times a week, with training lasting approximately 60 min<br><br>*a double-blind, randomized, placebo controlled, crossover study	2 s for eccentric and 2 s for concentric phase<br>**Days/week**: After 72 h for each visit<br>**Intensity**: until concentric failure with a load of 10 RM	(breakfast was supplied) before each test		
66	Phillips 2014	Design: RCT<br>Location: UK	Healthy Adults, male (n=12); Age 23.1 years, mean±SD 3.0; BM : 86.3, mean±SD 13.5 kg; Height: 1.83 ± 0.07 cm.<br>**Participants** were physically active and regularly undertook cycle ergometer sprinting as part of their normal physical activity routine.<br>*counterbalanced, cross-over design with simple randomization of trial orders and double-blind administration of mouth rinses.	**Duration**: 4 sessions<br>**Intervention: group**<br>**Type**: Cycle ergometer;<br>5 min warm up at 50- 60 rpm against a 1.5 kg resistance, 3 second countdown after which the participant cycled maximally for 30 sec 110 rpm<br>**Days/week**:1 days/week<br>**Intensity**: cycled maximally, the investigator introduced a resistance equivalent to 0.075 g·kg-1MB<br><br>Participants were requested to refrain from vigorous exercise at least 24 hours before each trial	**Duration**: 4 sessions<br>**Type**: Requested to refrain from alcohol for at least 24 hours before each trial, to consume their breakfast meal 2 hours prior to the trial and then to consume only water for the 2-hour pre-trial period.	**Type**: At 6 min, participants were provided with 25 ml of water and asked to rinse it around their mouth for 5 sec. Every 2 min until the beginning of the cycle sprint participants repeated the mouth rinse procedure 6, 8, 10, 12, 14, 16, 18, and 20 min; 200 ml water<br>**Intervention:** The experimental mouth rinse was a 6% (w/v) maltodextrin solution with a commercially available electrolyte tablet; The electrolyte tablet contained a small amount of artificial sweetener (Saccharine)<br>**Placebo:** The PLA solution comprised of one electrolyte tablet dissolved into 1 liter of water	<u>Peak power W</u><br>**Intervention group** mean±SD<br>PPO: 55.6 ± 30.4<br>**Placebo group** mean±SD<br>PPO: 48.3 ± 29.3<br><u>Mean power W</u><br>**Intervention group** mean±SD<br>MPO: 57.65 ± 6.65<br>**Placebo group** mean±SD<br>MPO :56.00 ± 6.33
67	Pires 2018	Design: RCT<br>Location: Brazil	Healthy Adults, male (n=9); Age 36.9 mean± 6.4 years; Weight 73.1±13.0 kg; Height 180.0 ± 5.0 cm;<br><br>**Participants** Cyclists; Physically active with regularly undertook cycle ergometer sprinting as part of their normal physical activity routine. 2 years taking part in regional competitions	**Duration**: 4 sessions<br>**Type**: Four Kilometer Cycling Time Trial;<br>5 min self-paced warm up, 2 min at a controlled pace warm up 80 rpm; 100 W<br>**Days/week**: visits were interspaced by 3–7 days.<br>**Intensity**: TT4km is a short trial completed under maximal effort	**Duration**: 4 sessions<br>**Type**: Consumed a standard breakfast 2 h before the onset of the procedures, after overnight fasting; A individualized diets with ~55% CHO, ~25% protein and ~20% fat, which was replicated during the 24 h preceding the sessions;<br>Participants were instructed to refrain from consumption of coffee, alcoholic and energy	**Type**: 25 ml CHO or PLA before the trial, for 10 s during the pace-controlled warm up routine. Cyclists rinsed their mouth only once, during the controlled pace warm up.<br>The CHO solution was made by diluting 64 g of glucose per 1000 mL of water, whilst PLA solution was made by diluting 36.2 g of a non-caloric sweetener containing saccharin in 1000 mL of water	**Intervention CHO** mean±SD<br>Time to complete 386.4 ± 28.0 s<br>Mean power (W) 275.4 ± 43.3<br>RPE 1.65 ± 0.53<br>**Placebo** mean±SD<br>Time to complete 385.4 ± 22.4 s<br>Mean power (W) 277.0 ± 35.0<br>RPE 2.04 ± 0.53<br><u>BASE LINE</u><br>**Intervention CHO** mean±SD<br>VA pre 91.56 ± 3.42<br>VA pre 83.97 ± 13.57<br><u>POST INTERVENTION</u>

			*a double-blind, counterbalanced fashion, after random designation	Participants were instructed to refrain from intense physical exercises for 72 h	beverages for 24 h before the sessions.		**Placebo** mean±SD<br>VA post 82.96 ± 13.24<br>VA post 90.42 ± 7.47
68	Pires 2024	Design: RCT<br>Location: Brazil	Healthy Adults, male (n=9); Age 36.9 mean± 6.4 years; Weight 73.1 ±13.0 kg; Height 180.0± 5.0 cm;<br>**Participants** Recreational cyclists having >2 years of experience in regional competitions<br>*a randomized, counterbalanced, and double-blind design	**Duration**: 4 sessions<br>**Type**: Cycling;<br>_5-min self-paced exercise followed by 1 min controlled-pace exercise 100 W pedaling at 80 rpm, the MIT increased 25W·min−1 until exhaustion.<br>**Days/week**: visits were interspaced by 3–7 days.<br>**Intensity**: maximal effort<br>They were asked to refrain from intense exercises during the 24 h preceding the experimental tests.	**Duration**: 4 sessions<br>**Type**: Consumed a standard breakfast 2 h before the onset of the procedures. Breakfast composed of ~55% CHO, ~25% protein, and ~20% fat;<br>They were asked to refrain from caffeine, alcohol, and other stimulant substances during the 24 h preceding the experimental tests.	**Type**: 25mL of CHO or PLA solution for the last 10 s of the controlled-pace warm-up.<br>**Intervention:** The CHO solution was made by diluting 64 g of glucose per 1000 mL of water;<br>**Placebo:** The PLA solution was made by diluting 36.2 g of a non-caloric sweetener containing saccharin in 1000 mL of water	**Intervention CHO** mean±SD<br>PPO (W) 328.8 ± 42.0<br>VO2MAX (ml·kg−1·min−1) 51.9 ±7.0<br>Time to exhaustion(s) 610.4 ± 99.1<br><br>**Placebo** mean±SD<br>PPO (W) 332.3 ± 42.2<br>VO2MAX (ml·kg−1·min−1) 51.2 ±5.3<br>Time to exhaustion(s) 622.4 ± 94.8
69	Plibyslavska 2016	Design: RCT<br>Location: USA	Healthy Adults, Female (n=11); Age 20 ± 1 years; Height 164 ± 6 cm; Weight 59.6 ± 3.3 kg<br><br>**Participants** NCAA Division II soccer players<br><br>*a double-blinded counterbalanced crossover design	**Duration**:4 sessions<br>**Type**: one countermovement vertical jump (1VJ); four consecutive vertical jumps (4VJ); SP18-m (sprint 18-m); SR72 (sprint 72-m) included five changes of direction<br>**Days/week**: Were separated by 7 days each<br>**Intensity**: (1VJ) to jump once as high as possible; (4VJ) 100% voluntary effort as quickly as possible; SP18 as fast as possible; SR72 as fast as possible	**Duration**: 4 sessions<br>**Type**: Investigators provided participants with an evening meal the night before each trial at 19:00 h consisting of a deli sandwiches, fruit (apples and bananas), 1 oz. bag of chips and cookies; Caloric content of 925 total calories 317 kcal of CHO, 495 kcal of fat and 113 kcal of protein; received two 500-mL bottles of water- the night before the experimental trials and upon waking the morning of the trial	**Type**: Five min prior to the first 5-min scrimmage period and during the 1.5-min recovery period; 20 mL of beverage; , mouth rinse for 10–15 sec<br>**Intervention**: 60 g of maltodextrin was mixed and diluted for every 1000 mL of sport beverage in the CMR.<br>**Placebo**: Commercially available non-caloric sport beverage	1VJ (cm)<br>**Intervention** mean±SD<br>46.5 ± 3.6<br>**Placebo** mean±SD<br>47.0 ± 3.6<br>4VJ (cm)<br>**Intervention** mean±SD<br>41.1 ± 3.0<br>**Placebo** mean±SD<br>41.4 ± 3.0<br>SP18 (s)<br>**Intervention** mean±SD<br>2.87 ± 0.07<br>**Placebo** mean±SD<br>2.86 ± 0.09<br>SR72 (s)<br>**Intervention** mean±SD<br>16.63 ± 0.34<br>**Placebo** mean±SD<br>16.71 ± 0.46<br>RPE<br>**Intervention** mean±SD<br>**Placebo** mean±SD

70	Pottier 2010	Design: RCT<br>Location: Belgium	Healthy Adults, Female (n=16); Age 20 ±1 years; BM: 67±4 kg; BMI: 17 ±2 kg/m2; Height:167± 3 cm<br><br>**Participants** Trained endurance triathletes<br><br>*a cross-over, counterbalanced, double-blind design	**Duration**: 6 sessions<br>**Type**: Electrically braked cycle ergometer; 1 h high-intensity time trial on trained subjects; Time trial in which they had to accomplish a set amount of work 975± 85 kJ as quickly as possible;<br>**Days/week**: Tests were separated by at least 48 h<br>**Intensity**: High-intensity time trial<br><br>Subjects were asked to refrain from intense exercise the day before the test.	**Duration**: 6 sessions<br>**Type**: The day before the test, subjects were recommended to take in at least 400 g of CHOs in total; The use of alcohol was limited to one unit; On the day of the test, subjects were asked to eat a meal rich in CHOs but low in fat at least 3 h before the test; Caffeine-containing drinks were not to be consumed;	**Type**: The total rinsed amount of the solution was set at 14mL/kg body weight; 5sec mouth rinse<br>**Intervention:** A 100 mL of isotonic CES contained 5.4g sucrose, 0.46g glucose, 41.7mg Na1, 12.5mg K1, citric acid, salt, sodium citrate, natural flavor, monopotassium phosphate, partially hydrogenated soybean, cottonseed oils and artificial coloring<br>**Placebo:** Contained exactly the same ingredients, except that the CHOs were replaced by the non-caloric sweetener aspartame at a concentration of 0.33g/L of beverage	Power output W<br>**Intervention** mean±SD<br>256.5 ± 30.8<br>**Placebo** mean±SD<br>265.0 ± 26.4<br>Time to complete<br>**Intervention** mean±SD<br>64.1± 6.5<br>**Placebo** mean±SD<br>61.7 ± 5.1<br>Heart Rate bpm<br>**Intervention** mean±SD<br>157.08 ± 12.44<br>**Placebo** mean±SD<br>161.33 ± 11.78<br>RPE (Borg)<br>**Intervention** mean±SD<br>15.52 ± 1.69<br>**Placebo** mean±SD<br>15.44 ± 1.39
71	Rollo 2011	Design: RCT<br>Location: UK	Healthy Adults, Male (n=10); Age 26±6 years; BM 74.2±5.7kg; Height: 1.81± 0.06 cm<br><br>**Participants**<br>Recreational runners experienced accustomed to training and/or competitions lasting at least 1 h in duration.<br><br>*a double-blind random crossover design for the ingestion trials	**Duration**: 4 sessions<br>**Type**: Motorized treadmill, 1-h run protocol; A habituation trial and three main 1-h running trials; 5-min warm-up at a speed equivalent to 60% V˙ O2peak<br>**Days/week**: All trials were separated by 7 days<br>**Intensity**: Instruction from the principal investigator: this is a running performance test, run as far as you can in 60 min Participants were asked to avoid heavy exercise in the 48 h	**Duration**: 4 sessions<br>**Type**: Recorded their food intake in the 48 h before the first trial and replicated it before the subsequent trials; An overnight fast 14–15 hr; There were no significant differences in the average daily energy intake 13.1±1.6 MJ or quantities of CHO (465±97 g, protein 128±20 g, or fat (99±41 g consumed in this 48-h period	**Type**: Mouth rinsed solution without ingestion; 25 mL of solution; 30 min before, immediately before, and at 15-min intervals during the run i.e., at 15, 30, and 45 min. The solution was mouth rinsed for 5 sec;<br>**Intervention**: commercially available 6,4% carbohydrate–electrolyte beverage<br>**Placebo**: Solution was matched in formulation to the carbohydrate–electrolyte solution except that it did not contain CHO	HR (bpm)<br>**Intervention** mean±SD<br>161± 7<br>**Placebo** mean±SD<br>162 ± 9<br>RPE<br>**Intervention** mean±SD<br>17 ± 1<br>**Placebo** mean±SD<br>17 ± 2<br>Total distances<br>**Intervention** mean±SD<br>14,283 ± 758 m<br>**Placebo** mean±SD<br>14,190 ± 800 m
72	Rollo 2010	Design: RCT<br>Location: UK	Healthy Adults, Male (n=20);<br>**Performance group** (n-10)<br>Age 25 ± 4 years; BM (kg) 74.3 ± 5.5; Height 1.79 ± 0.06 cm;	**Duration**: 7 days<br>**Performers group**<br>**Type**: One-hour running performance trials;	**Duration**: 7 days<br>**Type**: Participants fasted for 13–15 h overnight (no food, only water);	**Type**: 25 mL of solution. In both studies, participants mouth rinsed the solutions immediately before and at 15, 30, and 45 min during the 1-h run; Total volume	**Intervention CHO** mean±SD<br>Distance 14,298 ± 685 m<br>Mean running speed 14.3±0.7km·h^{-1}<br>% V˙O2peak 77 ± 7<br>Mean HR 163 ± 13

			**Resting group** (n-10) Age 26 ± 3 years; BM (kg) 76 ± 5; Height 1.74 ± 0.13 cm; **Participants** Experienced runners accustomed to training and/or competitions lasting at least 1 h in duration	5-min warm-up at 60% V˙O2peak; and 1 hour running in treadmill **Intensity**: ''This is a running performance test, run as far as you can in 60 min.'' **Resting group** For the resting study, participants sat quietly in a laboratory recovery room for 20 min before the start of the 1-h resting period. Asked to refrain from heavy exercise 48 h before each trial	There were no significant differences between trials in the average daily energy intake 14.4 ± 1.5 MJ or quantities of CHO 6.5 ± 1.5 g·kg^{-1} BM, protein 2.0 ± 0.4 g·kg^{-1} BM, or fat 1.5 ± 0.5 g·kg^{-1} BM consumed in the 48 h before each trial	of 100 mL of solution; The solution was mouth rinsed for 5 sec **Intervention:** The solutions were a commercially available 6.4% CHO–E beverage **Placebo:** A color and taste matched placebo solution, matched in formulation to the CHO–E solution except that it contained no CHO	RER 0.89 ± 0.03 **Placebo** mean±SD Distance 14,086 ± 732 m Mean running speed 14.1±0.7 km·h^{-1} % V˙O2peak 78 ± 7 Mean HR 163 ± 12 RER 0.88 ± 0.04
73	Rollo 2015	Design: RCT Location: UK	Healthy Adults, Male (n=11); Age 22 ± 3 years; Height (m) 1.78 ± 0.10 Weight (kg) 75.0 ± 7.6 **Participants** Soccer Players *double-blinded counterbalanced, crossover design.	**Duration**: 2 sessions **Type**: 90-minute field test modified, 6 blocks **Days/week**: Separated by 1week **Intensity**: It includes specific blocks of varying intensities (walking, max speed, 55% and 95% VO2max speeds) In the 24 hr preceding each experimental trial participants were asked to abstain from strenuous exercise	**Duration**: 2 sessions **Type**: In the 24 hr preceding each experimental trial participants were asked to abstain from caffeine, and alcohol. On the morning of each trial participants were asked to consume a breakfast that rep- resented their usual match-day meal 3 hr before exercise.	**Type**: 25 ml of experimental solution for 10 sec; The CHO was a 10% maltodextrin solution; The placebo (PLA) solution was water[; Rinsed a total of 11 times	BLOCK 6 (75–90 min) **Intervention CHO** mean±SD Distance covered (km) 2.0 ± 0.0 Mean speed (km·h^{-1}) 8.8 ± 0.1 15-m sprint time (s) 2.65 ± 0.13; Distance covered (km) 0.16 ± 0.10; Mean speed (km·h^{-1}) 20.4 ± 1.0 Recovery time Total (s) 76 ± 13 Heart rate (beat·min^{-1}) 165 ± 12 RPE 16.3 ± 2.2 **Placebo** Distance covered (km) 2.0 ± 0.1 Mean speed (km·h^{-1}) 8.7 ± 0.5 15-m sprint time (s) 2.69 ± 0.18 Distance covered (km) 0.16 ± 0.10; Mean speed (km·h^{-1}) 20.1 ± 1.2 Recovery time Total (s) 78 ± 22 Heart rate (beat·min^{-1}) 163 ± 13 RPE 16.4 ± 1.8
74	Rollo 2008	Design: RCT Location: UK	Healthy Adults, Male (n=10); Age 23 ± 4 years; Height (m) 1.81 ± 7 BM 75.0 ± 7 kg **Participants** Endurance-trained recreational runners	**Duration**: 4 sessions **Type**: Running; All tests were carried out on a motorized treadmill; Involved 40 min; 2-min walk at 4 km/h; 10-min warm-up run at a speed equivalent to 60% VO2max and began the 30-min trial.	**Duration**: 4 sessions **Type**: In the 48 hours leading up to each trial, each runner was asked not to consume caffeine or alcohol; On the morning of each trial runners reported to the	**Type**: 6.0-g/100-ml CHO solution or a taste-matched placebo (PLA) for 5 sec; Was administered immediately before the warm-up, and at 3 min, 6 min, and 9.5 min during the warm-up run; During the 30-min run the mouth rinse was	**Intervention CHO** mean±SD Total Distance covered (m) 6,584 ± 520 Energy expenditure (kcal · kg BM^{-1} · km^{-1}) 493 ± 54 kcal Mean Running speed (km/hr) 13.2 ± 1.1 **Placebo** mean±SD

			which regularly ran for 30–60 min, 3–5 days a week.<br><br>*a randomized, double-blinded crossover design.	**Days/week**: Separated by 7 days<br>**Intensity**: The runners were asked to select a speed that they perceived to be equivalent to a "hard pace" RPE of 15 on the 6-20 Borg scale.	laboratory after an overnight fast;<br>48 hr before exercise, 10.3 ± 3.2 MJ/day, CHO 5.1 ± 1.7, fat 1.1 ± 0.6, protein1.3 ± 0.4 g·kg $BM^{-1} \cdot day^{-1}$	administered at 5-min intervals; 25 ml bolus, 10 times equating to 250 ml of solution	Total Distance covered (m) 6,469± 515<br>Mean Running speed (km/hr) 12.9 ± 1.3<br>Energy expenditure (kcal · kg $BM^{-1} \cdot km^{-1}$) 485 ± 55 kcal
75	Rossato 2019	Design: RCT<br>Location: Brazil	Healthy Adults, Male (n=10); Age 24.1 ± 4.3 years; Body mass index (kg/m2) 21.6 ± 1.8; Weight (kg) 65.2 ± 8.0; Height (m) 1.73 ± 0.07<br><br>**Participants** Runners which had at least three years of experience in running and presented an average of training frequency of six times per week<br><br>*a randomized double-blind, placebo-controlled, and crossover study	**Duration**: 6 visits<br>**Type**: Running in treadmill;<br>5 min warm-up 65% of VO2max, rested for 3 min, and subjects commenced their run to exhaustion test on the treadmill<br>**Days/week**: a 7 days interval between visits<br>**Intensity**:100% of individual's VO2max until voluntary exhaustion.<br><br>They were instructed to refrain from physical exercise within 24 hours prior to the test	**Duration**: 6 visits<br>**Type**: Overnight fasting (mean of ~8 h across all trials);<br>They were instructed to refrain from caffeine or alcohol intake within 24 hours prior to the test	**Type**: 50 ml solution; The mouth rinse was performed for 10 sec immediately prior to each run to exhaustion test; **Intervention:** CHO with maltodextrin with a concentration of 6% plus industrialized noncaloric juice and lemon taste;<br>**Placebo:** A placebo solution with only industrialized noncaloric juice and lemon taste	**Intervention CHO** mean±SD<br>Time to exhaustion<br>195.1 ± 51.8 s<br>HR 81.6±14.6 to 185.6±13.2 b·min-1<br>**Placebo** mean±SD<br>Time to exhaustion<br>193.9 ± 46.5 s<br>HR 78.1±13.9 to 180.6±22.5 b·min-1
76	Rowlatt 2016	Design: RCT<br>Location: UK	Healthy Adults, (n=9); Male (n=7); Female (n=2); Age 31.2 ± 14.3 years; BM 81.4 ± 16.5 kg; Height 178 ± 8 cm<br><br>**Participants** National level fencers<br><br>*a single-blinded experiment with conditions randomised for order.	**Duration**: 2 sessions<br>**Type**: 5-min moderate intensity warm-up, consisting of 2 minutes jogging, 2 minutes stretching and 1 minute of fencing-specific footwork exercises; Two trials involving a fencing-specific skill test both pre- and post-completing a fencing fatiguing protocol; The fatigue protocol simulated a series of six fights replicating the first round of a standard fencing competition; Bouncing movements with a standardised number of arm extensions, retreats and lunges for a period	**Duration**: 2 sessions<br>**Type**= For each visit to the laboratory the participants were required to fast, following breakfast, for a minimum of 4 hours prior to the start of testing on each day.	**Type**= 25 ml mouth rinse between each fight for 10 sec<br>**Intervention**: Participants mouth rinsed 25 ml of 6.7% maltodextrin mixed into water<br>**Placebo:** 25 ml of water;	**Intervention CHO** mean±SD<br>RPE arm 10.9 ±2.5<br>RPE legs 12.6 ±2.8<br>RPE overall 12.6 ±1.8<br>Heart Rate (beats min^{-1}) 159 ±4<br>**Placebo** mean±SD<br>RPE arm 10.9 ±2.3<br>RPE legs 13.0 ±2.4<br>RPE overall 13.4 ±1.4<br>Heart Rate (beats min^{-1}) 158 ±7<br>**Intervention CHO** mean±SD<br>Hits on target (%) Pre<br>81.2 (±8.3)<br>Hits on target (%) Post<br>87.6 (±9.4)<br>**Placebo** mean±SD<br>Hits on target (%) Pre

				of 8 seconds before 9 seconds rest; They rested for a maximum of 6 minutes between poules<br>**Days/week**: separated by a minimum of five days<br>**Intensity**: no			82.1 (±8.8)<br>Hits on target (%) Post<br>78.8 (±6.4)
77	Shirai 2022	Design: RCT<br>Location: Switzerland	Healthy Adults, Male (n=8); Age 22.3 ± 1.3 years; Weight (kg) 67.0 ± 6.63; Height 171.9 ± 4.7 cm; Fat (%) 21.1 ± 3.15<br><br>**Participants** College students<br><br>*a randomized crossover design	**Duration**: 4 experimental trials<br>**Type**: Cycling exercise protocol; Constant load exercise for 60 min (intensity 40% VO2peak), four sets of the Wingate test (three 30 s Wingate tests with a 4 min recovery between each test), and a constant-load exercise for 30 min; the "average power value" for 30 s and the "maximum power value" in which the power value per 5 s reached the maximum value were evaluated.<br>**Days/week**: Each trial was performed at least 1 week apart.<br>**Intensity**: intensity 40% VO2peak<br><br>They were instructed not to engage in any strenuous physical activity during the 24 h period prior to each test	**Duration**: 4 experimental trials<br>**Type**: They were instructed not to consume caffeine, or drink alcohol during the 24 h period prior to each test; They were also advised to fast from 21:00 the day before the test.	**Type**: CHO mouth rinse (CMR) 25 mL of the mouth rinse solution for 10 sec; a 6% glucose solution (G); 6% CMR, 6% CMS, and water (WAT)<br>Following the first set of the Wingate test, participants used CHO mouth rinse during the three sets of constant-load exercise; In the CMS trials, participants used the CHO mouth spray during the three sets of constant-load exercise; (Participants were free to drink water in all trials, and their water intake was recorded)	Mean exercise power output (Watt)<br>Third set<br>average power values<br>WAT (88.65 ± 2.6%)<br>G 94.9 ± 4.0%,<br>CMR 96.12 ± 3.8%<br>CMS 96.5 ± 3.2%<br>Fourth set<br>average power values<br>WAT 83.5 ± 4.0%<br>G 89.6 ± 6.8%<br>CMR 91.6 ± 5.4%<br>CMS 93.4 ± 5.3%
78	Simpson 2018	Design: RCT<br>Location: USA	Healthy Adults, Male (n=7); Age 28.2 ± 6.8 years; Weight (kg) 85.3 ± 17.8; Height 185 ± 9 cm<br><br>**Participants** Athletes; 4 cyclists and competed in on and off-road cycling and 3 runners trained primarily for running competitions	**Duration**: 2 sessions<br>**Type**: Cycle ergometer; Multiple sprint performances in a protocol designed to emulate a cyclocross or mountain biking event; The workload was then increased by 0.5 kp every minute thereafter until a VO2 peak; 5 min warm-up 50% of VO2peak, The exercise protocol consisted of, 8 min	**Duration**: 2 sessions<br>**Type**: Participants refrained from consuming alcohol, tobacco, and caffeine 24 hr prior to testing; fasted overnight; An evening meal was provided although food volume was self-selected; 150 g of cooked white spaghetti, 128 g of tomato basil sauce, 96 g of cooked 93% lean hamburger, a serving of	**Type**: 25 mL of either carbohydrate or PLA solution; they rinsed the solution in their mouth for 10 sec; At minute 4:30 during each 5 minute steady state cycling segment; One solution consisted of the caloric sport beverage while the other was the caloric sport beverage with 64 g of maltodextrin that was mixed and diluted for every	**Intervention CHO** mean±SD<br>Fatigue Index W/sec 26 ± 13<br>Mean power w/kg 10.51 ± 0.82<br>RPE 15.8 ± 2.5<br>**Placebo** mean±SD<br>Fatigue Index W/sec 27 ± 15<br>Mean power w/kg 10.33 ± 0.87<br>RPE 16.4 ± 2.4

			*a randomized, double-blind crossover design	per set: 5 min of steady state exercise followed by 3 x 10 sec Wingate sprints (repeated 6 times) 48min exercise protocol **Days/week**: 2 sessions separated by at least 48 h, but no longer than 2 weeks between trials **Intensity**: high intensity intermittent exercise protocol.	strawberry yogurt, 1 banana, 1 apple, and 1 serving of chocolate cookies and had a caloric content of 988 total calories 593 kcal of carbohydrate, 258 kcal of fat and 137 kcal of protein	1000 mL of the sport beverage in the CMR	
79	Sinclair 2014	Design: RCT<br>Location: UK	Healthy Adults, Male (n=11); Age 24.1± 3.9 years; BM 77.9 ± 7.1 kg; Height 174.1± 3.0 cm<br><br>**Participants** Recreational cyclists; had previous experience of cycle ergometry	**Duration**: 4 sessions **Type**: They used a cycle ergometer; Maximum distance over 30 min, Prior to data collection participants completed a standardized warm-up 5 min of cycling (50W) **Days/week**: 1/week **Intensity**: maximum<br><br>All participants reported to the laboratory having abstained from exercise in the 24 hours prior	**Duration**: 4 sessions **Type**: All participants reported to the laboratory 4 hours post-prandial, having also abstained from alcohol, caffeine in the 24 hours prior to data collection;	**Type**: Each participant was given a 25 ml bolus; for 5 and 10 sec mouth rinse **Intervention**: A tasteless 6.4% maltodextrin (CHO) for every 6 min of the total protocol **Placebo:** Water (PLA) for every 6 min of the total protocol	<u>5-s rinse</u><br>**Intervention CHO** mean±SD<br>Power Output (W) 152.35± 17.42<br>Heart rate (beats.min^{-1}) 164± 9<br>RPE (Borg scale) 12.35± 1.17<br>Speed (km.h^{-1}) 37.95± 3.95<br>Cadence (RPM) 91.18± 9.76<br><br><u>10-s rinse</u><br>**Intervention CHO** mean±SD<br>Power Output (W) 155.63± 17.05<br>Heart rate (beats.min^{-1}) 165± 7<br>RPE (Borg scale) 12.24± 1.58<br>Speed (km.h^{-1}) 38.66± 4.13<br>Cadence (RPM) 92.95± 9.69<br>Distance cycled 20.4 ±2.3 km<br><br>**Placebo** mean±SD<br>Power Output (W) 145.73±13.55<br>Heart rate (beats.min^{-1}) 168±10<br>RPE (Borg scale) 13±1.65<br>Speed (km.h^{-1}) 36.06±4.40<br>Cadence (RPM) 87.39± 10.07<br>Distance cycled 19.2± 2.2 km
80	Trommelen 2015	Design: RCT<br>Location: Canada	Healthy Adults, Male (n=14); Age 27 ± 6 years; BM 78 ± 11 kg; Height 1.83 ± 0.08 cm;<br><br>**Participants** Cyclists or triathletes; All participants had been engaged in regular	**Duration**: 6 sessions **Type**: Electronically braked cycle ergometer; 1.2 visits experimental trials and 4-time trials of ~1 hr (1,032 ± 127 kJ); **Days/week**: Trials separated by at least 1 week	**Duration**: 6 sessions **Type**: The evening before each experimental trial, participants consumed a standardized dinner (68 ± 4 kJ/kg, providing 60 ± 5 energy% (En%) carbohydrate, 25 ± 2 En% fat, and 15 ± 2 En% protein), after	**Type**: Each participant was given a 25 mL bolus of either a 6.4% SUC (sucrose solution) or a noncaloric 0.6% aspartame sweetened PLA at the start and after every 12.5% of the time trial completed; Participants	<u>Performance time fasted</u><br>**Intervention CHO** mean±SD<br>fasted-SUC 69.6 ± 7.5 min<br>**Placebo** mean±SD<br>fasted-PLA 68.6 ± 7.2 min<br><u>Performance time fed</u><br>**Intervention CHO** mean±SD<br>fed SUC 69.0 ± 6.3 min

			cycling training (>4 times/ week) for more than 6 years<br><br>*a double-blind, counterbalanced order	**Intensity**: The ergometer was set in linear mode so that 75% Wmax; Preferred cycling cadence 100 ± 8 rpm, determined during the maximal workload capacity test<br><br>*Refrained from physical exercise and exhaustive physical labor for 24 hr before each experimental trial	which they remained fasted; A standardized breakfast was consumed 2 hr before starting the time trial. This breakfast provided 36 ± 2 kJ/kg (65 ± 7 En% carbohydrate, 18 ± 4 En% fat, and 17 ± 3 En% protein) and was composed of bread with butter, cheese, and marmalade; a slice of gingerbread; and a glass of orange juice.	rinsed the fluid around their mouth for 5 sec	**Placebo** mean±SD<br>fed PLA 67.6 ± 6.6 min<br>Average power output fasted<br>**Intervention CHO** mean±SD<br>252 ± 46 W<br>**Placebo** mean±SD<br>255 ± 43 W<br>Average power output fed<br>**Intervention CHO** mean±SD<br>253 ± 41 W<br>**Placebo** mean±SD<br>258 ± 45 W<br>Mean heart rate fasted<br>**Intervention CHO** mean±SD<br>160 ± 10 bpm<br>**Placebo** mean±SD<br>161 ± 8 bpm<br>Mean heart rate fed<br>**Intervention CHO** mean±SD<br>163 ± 8 bpm<br>**Placebo** mean±SD<br>164 ± 10 bpm<br>Mean rpm fasted<br>**Intervention CHO** mean±SD<br>96 ± 9 rpm<br>**Placebo** mean±SD<br>97 ± 7 rpm<br>Mean rpm fed<br>**Intervention CHO** mean±SD<br>98 ± 8 rpm<br>**Placebo** mean±SD<br>98 ± 9 rpm
81	Watson 2014	Design: RCT<br>Location: UK	Healthy Adults, Male (n=10); Age 21 ± 2 years; BM 74.2 ± 5.3 kg; Height, 1.79 ± 0.09 m<br><br>**Participants** All volunteers were experienced road cyclists who regularly took part in cycle training and competitive races	**Duration**: 4 sessions<br>**Type**: Electrically braked cycle ergometer; A 5-min warm-up at 50% Wmax and stretch; Started the time trial for 1-h (as much work as possible in a 1-h period;) The initial workload was set at 75% Wmax	**Duration**: 4 sessions<br>**Type**: Subjects were instructed to ingest 500 mL of plain water at 90 min before the scheduled start of the trial; Trials took place in the morning, following an overnight fast;	**Type**: At the start, and after every 10 min of the time trial, subjects ingested 100 mL of plain water; Subjects were provided with a 25-mL bolus; Subjects rinsed their mouths for 10 seconds; Immediately before, and at regular intervals throughout exercise	Total work kJ<br>**Intervention CHO** mean±SD<br>762.6 ± 141.1 kJ<br>**Placebo** mean±SD<br>758.8 ± 149.0 kJ<br>Mean power W<br>**Intervention CHO** mean±SD<br>231± 41 W<br>**Placebo** mean±SD

			*a double-blind, counterbalanced, crossover manner	**Days/week**= each visit separated by at least 7 days.<br>**Intensity**: Volunteers were encouraged to produce a maximal effort.<br><br>No strenuous exercise was permitted in the 24 h before each trial.	No alcohol consumption was permitted in the 24 h before each trial.	**Intervention**: A 6.4% CHO sugar-free fruit drink with 6.4 g/L of glucose solution<br>**Placebo**: A sugar-free fruit drink	**232** ± 42 W<br><u>Heart rate</u><br>**Intervention CHO** mean±SD<br>184 ± 13 beats/min<br>**Placebo** mean±SD<br>181 ± 13 beats/min<br><u>RPE</u><br>**Intervention CHO** mean±SD<br>19 ± 1<br>**Placebo** mean±SD<br>19 ± 1
82	Williamson-Reisdorph 2022	Design: RCT<br>Location: USA	Healthy Adults, Male (n=10); Age 30 ± 6 years; BM 80.6 ± 8.5 kg; Height 180 ± 5.4 cm; Body Fat (%) 17.7 ± 4.5<br><br>**Participants** Recreational cyclists; The inclusion criteria required that each participant trained at least twice per week for a minimum of one-hour<br><br>*a double-blind, placebo-controlled, randomized crossover design with subjects serving as their own control.	**Duration**: 3 sessions<br>**Type**: 1-hour cycling work; Cycle ergometer; The initial visit consisted of a review of the procedures, a VO2max test, and a five-minute familiarization period; Experimental Trials with warm-up of 5 min with 100 watts, the ergometer then transitioned to the 1-hour WCT<br>**Days/week**: Three separate occasions with three to nine days between visits<br>**Intensity**: The cycle ergometer was set to the linear mode that 75% Wmax for one hour<br><br>Participants were required to abstain from exercise for 24 hours.	**Duration**: 3 sessions<br>**Type**: Participants were required to fast for a period of 10-hours, abstain from alcohol for 24-hours, and avoid caffeine for 12- hours before testing sessions	**Type**: 25 mL being administered every 12.5% of work completed (8 total rinses) during the trial; Trials were performed with;<br>**Intervention**: CHO solution selected for this study was 6.4%;<br>Placebo: A placebo (PLA) solutions artificially sweetened beverage;<br>Participants were able to drink water at two time points during the 1-hour up to 450 ml of water	<u>Time to Completion (min)</u><br>**Intervention CHO** mean±SD<br>69.6 ± 6.2<br>**Placebo** mean±SD<br>68.5 ± 5.1<br><u>Average Heart Rate (bpm)</u><br>**Intervention CHO** mean±SD<br>160 ± 18<br>**Placebo** mean±SD<br>164 ± 14<br><u>Average Pedal Rate (RPM)</u><br>**Intervention CHO** mean±SD<br>76.8 ± 6<br>**Placebo** mean±SD<br>76.0 ± 5<br><u>Maximum Heart Rate (bpm)</u><br>**Intervention CHO** mean±SD<br>178 ± 18<br>**Placebo** mean±SD<br>179 ± 14
83	Wright 2013	Design: RCT<br>Location: UK	Healthy Adults, Male (n-7); Age 21 ± 1 years; BM 78 ± 7 kg; Height 1.81 ± 0.12 cm<br><br>**Participants** University students; Represented the university in a competitive sports team (e.g. football [soccer], rugby, field hockey)	**Duration**: Four trials<br>**Type**: Two different concentrations (6% and 12% weight/volume, w/v) on 90 min treadmill running performance; 5 min warm up at 6 km/h; The test began with a rolling start at a treadmill speed of 8 km/h; Allowed to freely control the treadmill speed	**Duration**: Four trials<br>**Type**: Participants were required to be fasted for at least six hours before each trial;	**Type**: Mouth rinse for 5 sec; The first mouth rinse procedures occurred after the warm-up and then at 15, 30 and 45 minutes of the performance trial<br>**Intervention**: The CHO was comprised of maltodextrin 95%, dextrose 3% and maltose 2%; CHO concentrations of 6% and 12% w/v, with approximately	<u>Distance (km)</u><br>**Intervention** 6% CHO 14.6 ± 1.7<br>**Intervention** 12 % CHO 14.9 ± 1.6<br>**Placebo** 13.9 ± 1.7<br><u>Heart Rate (bmp)</u><br>**Intervention** 6% CHO 180± 14<br>**Intervention** 12 % CHO 180±12<br>**Placebo** 179±14<br><u>RPE</u><br>**Intervention** 6% CHO 18±1

			*The main trials were conducted in randomized order and solutions were administered double-blind.	**Days/week**: not mentioned<br>**Intensity**: They were instructed to cover as much distance as possible during the 90 min test		418 mg and 836 mg of sodium per liter in the 6% and 12% solutions, respectively;<br>**Placebo**: The PLA solution did not have any of this powder added;	**Intervention** 12 % CHO 18±1<br>**Placebo** 17±1<br><u>Oxygen uptake 45 min</u><br>**Intervention** 6% CHO 3.15±0.47<br>**Intervention** 12 % CHO 2.98±0.37<br>**Placebo** 2.96 ± 0.34
84	Yang 2024	Design: RCT<br>Location: Switzerland	Healthy Adults, Male (n=20); Age 22.4 ± 3.7 years; Weight: 76.4 ± 11 ± kg; Height: 171.3 ± 5.9 cm<br>**CMR trial** (n=10)<br>**PLA trial** (n=10)<br><br>**Participants** With regular weight training habits<br><br>*a randomized, crossover trial with a double-blind experimental design.	**Duration**: 3 trails<br>**Type**: Inertial resistance exercise machine; Romanian deadlift movement test; One formal experiment, warm-up, bicycle for 5 min, joint range-of-motion warm-up and dynamic stretching for 3 min; Five sets of five reps, with a 3 min rest between<br>**Days/week**: Tests separated by 7 days<br>**Intensity**: maximum inertial resistance<br><br>Avoid high-intensity training (e.g., weight training, endurance training, and high-intensity interval training) for 3 days before the experiment	**Duration**: 3 trails<br>**Type**: The nutritional composition of breakfast and lunch was 13.9 ± 1.4% protein, 50.3 ± 12.7% carbohydrate, 35.8 ± 5.2% lipid, and 1423.6 ± 111.4 kcal; To fast for at least 3 hours beforehand; Also, all participants were asked to avoid caffeine, alcohol, energy drinks, and any form of stimulant or other substance that would affect exercise performance	**Type**: The participants rinsed their mouths with 25 mL for 20 sec;<br>**Intervention**: CHO solution before exercise with 6.4% of colorless and odorless maltodextrin solution in 93.6 mL of water<br>**Placebo**: Before exercise; The placebo group rinsed their mouths with drinking water	<u>Peak Concentric power</u><br>**Intervention CHO** mean±SD<br>584.1 ± 136.0 W<br>**Placebo** mean±SD<br>522.9 ± 122.9 W<br><u>Peak Eccentric Power</u><br>**Intervention CHO** mean±SD<br>682.4 ± 145.4 W<br>**Placebo** mean±SD<br>596.4 ± 151.1 W<br><u>Average power</u><br>**Intervention CHO** mean±SD<br>296.2 ± 70.1 W<br>**Placebo** mean±SD<br>261.7 ± 67.1 W<br><u>Average Force</u><br>**Intervention CHO** mean±SD<br>512.3 ± 211.0 N<br>**Placebo** mean±SD<br>446.7 ± 177.5 N<br><u>Total Work</u><br>**Intervention CHO** mean±SD<br>17.3 ± 3.7 KJ<br>**Placebo** mean±SD<br>15.5 ± 3.8 KJ<br><u>Heart Rate</u><br>**Intervention CHO** mean±SD<br>130.0 ± 9.6 bpm<br>**Placebo** mean±SD<br>126.6 ± 14.1 bpm<br><u>RPE</u><br>**Intervention CHO** mean±SD<br>11.9 ± 2.4<br>**Placebo** mean±SD

							11.8 ± 2.0
85	Bottoms 2014	Design: RCT<br>Location: UK	Healthy Adults, Male (n=12); Age 20.5±0.7 years; BM 87.4±18.3 kg; Height 170.5±18.8 cm<br><br>**Participants** were recreationally trained cyclists<br><br>*a blinded counterbalanced design	**Duration**: 4 sessions<br>**Type**: 30 min cycle ergometer for maximum distance; 5 min warm-up of cycling using a resistance of 50 W;<br>**Days/week**: each session was separated by 7 days<br>**Intensity**: self-paced cycling trial<br><br>Had not conducted any vigorous exercise in previous 24 h	**Duration**: 4 sessions<br>**Type**: Participants were examined 4 h post prandial; Had not consumed any alcohol or caffeine in previous 24 h	**Type**: 25 ml bolus of a tasteless CHO or PLA for every 6 min of the total protocol; 5 sec mouth rinse<br>**Intervention:** a 6.4% maltodextrin (CHO) solution<br>**Placebo:** Water	Distance (km) mean±SD<br>**Intervention** 15.9±2.9 km<br>**Placebo** 14.9±2.6 km<br>Cadence(rev/min) mean±SD<br>**Intervention** 77.0±13.7<br>**Placebo** 72.3±12.5<br>CHO significant difference from the PLA<br>Speed(km/h) mean±SD<br>**Intervention** 32.3±5.6<br>**Placebo** 30.0±5.4<br>CHO significant difference from the PLA<br>Power output(W) mean±SD<br>**Intervention** 153.3±29.0<br>**Placebo** 145.3±23.5<br>HR(bpm) mean±SD<br>**Intervention** 162±24<br>**Placebo** 160±26<br>RPE (Borg scale) mean±SD<br>**Intervention** 13±2<br>**Placebo** 13±1
86	Chambers 2009	Design: RCT<br>Location: UK	Healthy Adults,<br>**Study 1A Oral glucose and exercise performance**<br>Male (n=8); Age 29 ± 9 years; Weight 77.1 ± 8.0 kg; BMI 23.8 ± 2.5 kg m^{-2}<br>**Study 2A Oral maltodextrin and exercise performance**<br>Male (n=6); Female (n=2); Age 22 ± 3 years; weight 69.7 ± 12.1 kg; BMI 22.3 ± 2.7 kg m^{-2}<br><br>**Participants** Endurance-trained cyclists	**Duration**: 4 sessions<br>**Type**: Cycling ergometer; Amount of work, equivalent to cycling for 1 h at 75% ˙Wmax (914±29 kJ in Study 1A; 837±68 k in Study 2A)<br>**Days/week**: Each visit separated by a period of at least 3 days<br>**Intensity**: Complete as fast as possible with the ergometer set so that 75% Wmax	**Duration**: 4 sessions<br>**Type**: The subjects in both studies fasted, except for water, overnight from 22:00	**Type**: Mouth rinse protocol. At the start, and after every 12.5% of the time trial completed, subjects were given 25ml of GLU, MALT or PLA; 10 sec mouth rinse<br>**MALT:** The MALT mouth rinse contained 64 g maltodextrin per 1000 ml of the same solution<br>**GLU**: The GLU mouth rinse contained 64 g glucose per 1000 ml of the same solution<br>**PLA**: Mouth rinse solution was made from150 ml of a commercially available non-caloric concentrate sweetened with aspartame and saccharin diluted to 1000 ml with water.	**Study 1A**<br>Time and power output<br>GLU: 60.4 ± 3.7<br>PLA: 61.6 ±3.8<br>RPE<br>GLU: 16 ± 1.8<br>PLA: 16 ± 1.6<br>Heart rate<br>GLU: 180 ± 3<br>PLA:177 ± 4<br>**Study 2A**<br>Time and power output<br>MALT: 62.6 ± 4.7<br>PLA: 64.6 ± 4.9<br>RPE<br>MALT: 17 ± 2.1<br>PLA: 17 ± 1.8<br>Heart rate

			*a randomised, counterbalanced and double-blind fashion.				MALT: 181 ± 10<br>PLA:180 ± 10
87	Karayigit 2021	Design: RCT<br>Location: Turkey	Healthy Adults, Male (n=15); Age 21.6 ± 1,3 years; Weight: 83.3 ±14.4 kg; Height: 181.2 ± 10,0 cm;<br><br>**Participants** who perform resistance training 2 hours a day at least 5 days a week in the last 1 year<br><br>*a randomized, counterbalanced, crossover and single-blind study design	**Duration**:<br>**Type**: Muscular Endurance Measurement; 5-min warming-up on the treadmill;<br>1RM to evaluate upper body strength; Muscular endurance for 1RM in 3 sets; 3 min passive rest was allowed between the sets<br>**Days/week**: There were 2-5 days between the test days<br>**Intensity**: maximum 1RM; Muscular endurance was measured by lifting 40% of 1RM until exhaustion<br><br>The participants were instructed o avoid performing physical activity in the last 24 hours	**Duration**:<br>**Type**: The participants were instructed not to consume alcohol and caffeine in the last 24 hours; 10 hr Night-long fasting	**Type**: Solutions were prepared in 25 ml water by masking with sucralose 200 mg; 10 sec mouth rinse before 1RM attempt and with 1-min before the muscular endurance test<br>**Intervention**: CHO: 6% weight/volume (1.5 gr) maltodextrin mouth rinse<br>**Placebo:** Water rinse	Strength<br>Bench press 1RM (kg)<br>**Intervention:** 98.21<br>**Placebo:** 97.50<br>Muscular endurance<br>Number of repetitions<br>**Intervention:**<br>1 set 25.21<br>2 set 18.57<br>3 set 15.35<br>**Placebo:**<br>1 set 25.14<br>2 set 18. 14<br>3 set 15.85<br>Muscular endurance (kg)<br>**Intervention:**<br>1 SET 970.50<br>2 SET 723.75<br>3 SET 594.28<br>**Placebo:**<br>1 SET 969.28<br>2 SET 968.92<br>3 SET 619.28<br>RPE<br>**Intervention:**<br>1 SET 17.57<br>2 SET 18.42<br>3 SET 19.14<br>**Placebo:**<br>1 SET 17.71<br>2 SET 19.00<br>3 SET 19.10<br>Heart Rate<br>**Intervention:** mean±SD<br>After 1RM 115.28 ± 14.60<br>After 1. set 128.28 ± 16.40<br>After 2. set 131.35 ±17.09

							After 3. set 135.78 ±18.08<br>**Placebo:** mean±SD<br>After 1RM 118.71 ± 13.80<br>After 1. set 130.85 ± 12.47<br>After 2. set 133.57 ±17.87<br>After 3. set 134.00 ± 16.01
88	Karayigit 2017	Design: RCT<br>Location: Turkey	Healthy Adults, Male (n=15); Age 20.00±2.44 years; BM: 75.02±6.42 kg; Height:177.66±4.80 cm<br><br>**Participants** Regional level amateur soccer players for the last 5 years<br><br>*a double-blind, randomized, counterbalanced and crossover research design	**Duration**: 3 sessions<br>**Type**: Cycle ergometer; 5-min warm up at 90-Watt 60 rpm; maximally of 4-sec sprint; 12x4-s intermittent sprint test protocol; 90 seconds of active recovery between sprints<br>**Days/week**: 4 sessions separated by 2-4 days within a 12-day period<br>**Intensity**: max sprint<br><br>Participants were asked to abstain from vigorous exercise for a minimum of 24 hours before each condition.	**Duration**: 3 sessions<br>**Type**: 10 h night fasting; Participants were asked to abstain from caffeine ingestion for a minimum of 24 hours before each condition	**Type**: 25 ml mouth rinse; Solutions was rinsed for 10 sec, immediately before each sprint and 12 times in total.<br>**Intervention:** 6.4% (w/v) tasteless and colourless maltodextrin solution<br>**Placebo:** Water	CHO mouth rinsing does not improve peak and mean power when compared to placebo
89	Suzuki 2020	Design: RCT<br>Location: Japan	Healthy Adults, Male (n=10); Age 19.0 ± 1.0 years; Body weight 55.4 ± 4.9 (kg); BMI 19.4 ± 0.9; Height 168.9 ±6.6<br><br>**Participants** collegiate distance runners<br><br>*a randomized, double-blind crossover trial.	**Duration**:<br>**Type**: All exercise tests were carried out on a cycle ergometer; 20 min warm-up - pedaling at about 80 rpm; 5 min rest and 6-sec peak power tests with full power pedaling with a load of 4% BW k 3 times at 1-minute intervals; 3 trials was recorded.<br>**Days/week**:<br>**Intensity**: Maximum power	**Duration**:<br>**Type**: Over- night fast from 21:00 the day before; Were allowed water during the fast	**Type**: 10 sec before the end of the rest, participants rinsed their mouths with a 25-mL bolus of a test drink for 5 sec<br>**Intervention:**<br>Trehalose (6% w/v) is a disaccharide consisting of 2 glucose units<br>**or** Maltose (6% w/v)<br>**or** Acesulfame potassium (0.04 mg/mL)	Mean power (W) 6 sec mean±SE<br>Acesulfame 321.0 ±1.6<br>Maltose 318.4 ±2.4<br>Trehalose 329.9 ±1.5<br><br>Mean power (W) 30 sec mean±SE<br>Acesulfame 179.3 ± 0.3<br>Maltose 171.9 ± 0.5<br>Trehalose 177.1 ± 0.7
90	Whitham 2007	Design: RCT<br>Location: UK	Healthy Adults, Male (n-7); Mean age 28.5 years; Mean Weight 72.8 kg; Mean Height 1.74 m<br><br>**Participants** were recreationally active and had	**Duration**: 4 sessions<br>**Type**: Treadmill running; Treadmill running; warm-up of 5min at 8km .h; 15 min preload exercise at a speed equating to 65% _V O2max; 45-min time-trial with the participant	**Duration**: 4 sessions<br>**Type**: 4 hr fasted state; Abstained from caffeine and alcohol in previous 24 hr; Participants were also provided with a standardized 816- kcal breakfast (percent	**Type**: At the start of each trial, each participant was issued with a 500-ml drinks bottle containing a 200-ml bolus of either carbohydrate or placebo; mouth rinse for 5 sec; At the start of the preload and every subsequent 6	Heart Rate (bpm)<br>Speed (km·h)<br>Distance (meters)<br>**Intervention**: 9333 m<br>**Placebo**: 9309 m

			previous running experience at distances of 10 km and above<br><br>*a randomized, double-blind, counterbalanced design	covering as much distance as possible<br>**Days/week**: Visits separated by a minimum of 5 days<br>**Intensity**: participants were instructed to cover as much distance as possible<br>Abstained from heavy exercise in previous 24 hr	energy contributions: carbohydrate 77.6%, fat 13.8%, protein 8.7%) for consumption 4 h before testing	min of both the preload and time-trial<br>**Intervention**: The carbohydrate mouthwash consisted of 6% maltodextrin 97% polysaccharide, 2% disaccharide, 1% glucose<br>**Placebo:** not mentioned	

MR= Mouth rinse**, SD**= Standard Deviation**, BM**= body mass, **CHO**= carbohydrate, **PLA**= placebo, **PPO**= peak power output, **MPO**= Mean power output, **MIT**= maximal incremental test, **rpm**= revolutions/minute, **MVC**= maximal voluntary contraction, **VO2max**= Maximum Oxygen Intake, **Wpeak**= Watt-peak (Peak Power**), VJ=** vertical jump, **SP**= sprint, **LIST**= Loughborough Intermittent Shuttle Test, **RPE**= Rating of Perceived Exertion, **W**= Watt, **RAST**= Repeated anaerobic sprint test, **CMR**= Carbohydrates mouth rinse, **Wmax**= maximum work output or maximum power, **kJ=** Work in kilojoules (Average Watts × Duration in Seconds) / 1000, **SUC**= sucrose solution, **WCT**= work to completion trial, **GMR=** Glucose mouth rinse, **BMI**= body-mass index, **RAMP**= Ramp Test is a progressive, incremental cycling test used to estimate your Functional Threshold Power (FTP), **LCHO**= Low dose of Carbohydrate, **MCHO**= Moderate dose of Carbohydrate, **HCHO**= High dose of Carbohydrate, **NaCl=** Salt, **Yo-Yo IRT-1**= Intermittent Recovery Test level-1 (maximal aerobic endurance test), **PTV**= peak treadmill velocity, **VA**= Voluntary Activation, **RPM= [**revolutions per minute (**Cadence** measures how fast a cyclist pedals)], **FST**= Fastest sprint time, **AST**= Average sprint time, **PRS**= Perceived recovery status, **SUC**= sucrose solution, **bpm=** beats per minute, **RTI**= Reaction time, **CF=** Cognitive function, **MD**= maltodextrin

Table S2: Risk of bias [1-90]

	First author	Randomization process	Intervention assignment	Intervention adherence	Missing data	Outcome	Reported results
1	Ali 2017	?	+	+	+	+	+
2	Ali 2016	?	+	+	+	+	+
3	Ataide-Silva 2016	?	+	+	+	+	+
4	Bailey 2019	?	+	+	+	+	+
5	Baltzar Martins 2019	?	+	+	+	+	+
6	Bastos-Silva 2016	?	+	+	+	+	+
7	Bastos-Silva 2019	?	+	+	+	+	+
8	Bataineh 2018	?	+	+	+	+	+
9	Bavaresco Gambassi 2018	?	+	+	+	+	+
10	Bazzucchi 2017	+	+	+	+	+	+
11	Beaven 2013	?	+	+	+	+	+
12	Beelen 2009	+	+	+	+	+	+
13	Black 2022	?	+	+	+	+	+
14	Black 2018	+	+	+	+	+	+
15	Bowling 2025	+	+	+	+	+	+
16	Carter 2004	?	+	+	+	+	+
17	Cherif 2018	?	+	+	+	?	+
18	Chiu 2022	?	+	+	+	?	+
19	Chong 2011	?	+	+	+	+	+
20	Chryssanthopoulos 2017	?	+	+	+	+	+
21	Clarke 2017	?	+	+	+	+	+
22	Clarke 2017	?	+	+	+	?	+
23	Clarke 2015	+	+	+	+	+	+
24	Cramer 2015	?	+	+	+	+	+
25	de Oliveira 2020	?	+	+	+	+	+
26	de Oliveira 2019	+	+	+	+	+	+
27	Decimoni 2018	+	+	+	+	+	+
28	Deighton 2016	?	+	+	+	+	+
29	Devenney 2016	+	+	+	+	+	+
30	Dorling 2013	+	+	+	+	+	+
31	Dolan 2017	+	+	+	+	+	+
32	Durkin 2021	?	+	+	+	+	+
33	Dunkin 2017	+	+	+	+	+	+
34	Evans 2021	?	+	+	+	+	+
35	Ferreira 2018	?	+	+	?	+	+
36	Ferreira 2018	?	+	+	+	+	+
37	Fares 2011	+	+	+	+	+	+
38	Fraga 2015	?	+	+	+	+	+
39	Green 2022	+	+	+	+	+	+
40	Gam 2013	?	+	+	+	+	+
41	Gam 2014	?	+	+	+	+	+
42	Hawkins 2017	?	?	?	+	+	?
43	Hirata 2021	+	+	+	+	+	+
44	Hsu 2025	+	+	+	+	+	?
45	Ispoglou 2015	+	+	+	+	+	?
46	James 2017	?	+	+	+	+	?
47	Jeffers 2015	?	+	+	+	+	?
48	Jensen 2015	+	+	+	+	+	+

49	Karayigit 2022	?	+	+	+	+	?
50	Karayigit 2021	?	+	+	+	+	?
51	Karayigit 2021	+	+	+	+	+	+
52	Karuk 2022	?	+	+	+	+	+
53	Khong 2020	+	+	+	+	+	+
54	Krings 2017	+	+	+	+	+	+
55	Kulaksız 2016	+	+	+	+	+	?
56	Lane 2013	?	+	+	+	+	?
57	Luden 2016	+	+	+	+	+	+
58	Mazman 2024	+	+	+	+	+	?
59	Muhamad 2020	+	+	+	+	+	?
60	Murray 2018	+	+	+	+	+	+
61	Nehme 2022	+	+	+	+	+	+
62	Nyman 2022	+	+	+	+	+	+
63	Oziukan Sahin 2024	?	+	+	+	+	+
64	Painelli 2011	+	+	+	+	+	+
65	Pereira 2020	+	+	+	+	+	+
66	Phillips 2014	+	+	+	+	+	+
67	Pires 2018	+	+	+	+	+	+
68	Pires 2024	+	+	+	+	+	+
69	Pribyslavska 2015	+	+	+	+	+	+
70	Pottier 2010	+	+	+	+	+	+
71	Rollo 2011	+	+	+	+	+	+
72	Rollo 2010	+	+	+	+	+	+
73	Rollo 2015	+	+	+	+	+	+
74	Rollo 2008	+	+	+	+	+	+
75	Rossato 2019	+	+	+	+	+	+
76	Rowlatt 2016	+	+	+	+	+	+
77	Shirai 2022	+	+	+	+	+	+
78	Simpson 2018	+	+	+	+	+	+
79	Sinclair 2014	+	+	+	+	+	+
80	Trommelen 2015	+	+	+	+	+	+
81	Watson 2014	+	+	+	+	+	+
82	Williamson-Reisdorph 2022	+	+	+	+	+	+
83	Wright 2013	+	+	+	+	+	+
84	Yang 2024	+	+	+	+	+	+
85	Bottoms 2014	+	+	+	+	+	+
86	Chambers 2009	+	+	+	+	+	+
87	Karayigit 2021	+	+	+	+	+	+
88	Karayigit 2017	+	+	+	+	+	+
89	Suzuki 2020	+	+	+	+	+	+
90	Whitham 2007	+	+	+	+	+	+

Figure S1: Risk of bias summary [1-90]

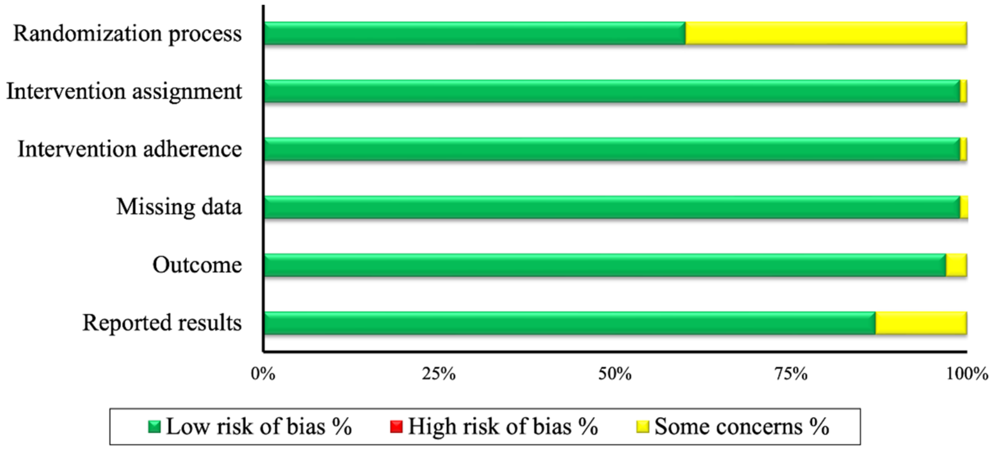

Figure S2: Forest plot for muscular endurance [6, 24, 34, 43, 51-55, 69]

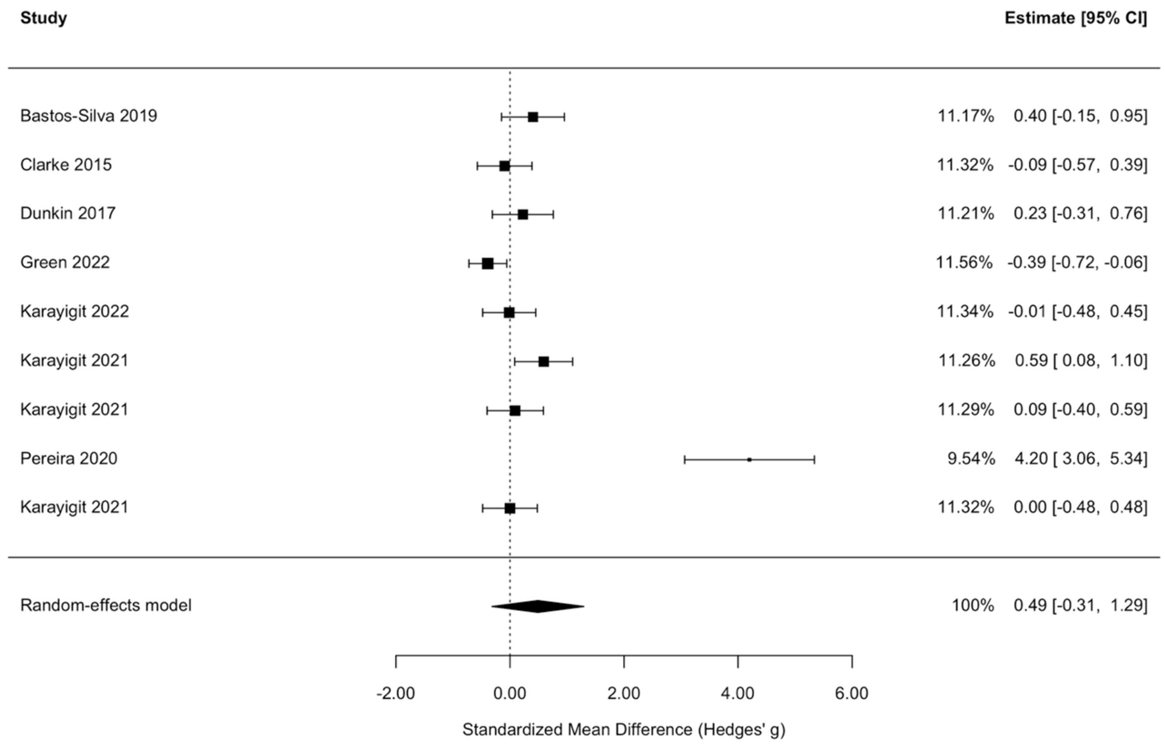

Figure S3: Forest plot for muscular endurance without Pereira et al. 2020 study [6, 24, 34, 43, 51-55]

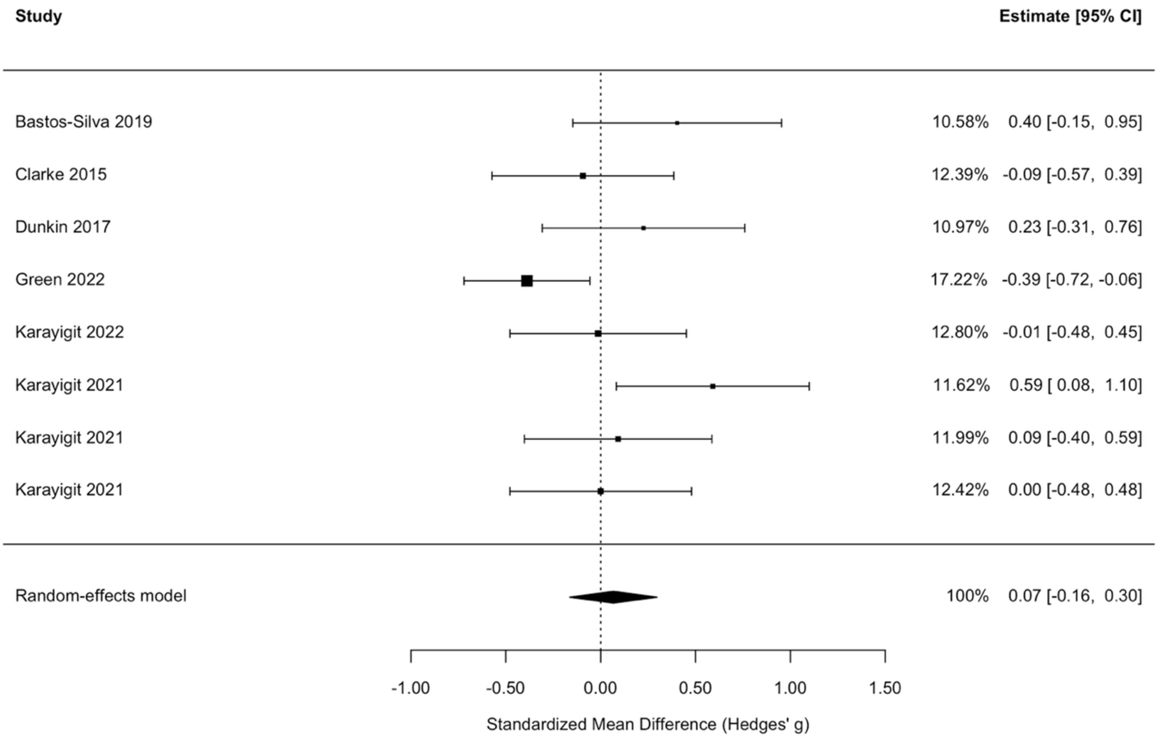

Figure S4: Funnel plot for distance covered [15, 22, 30, 32, 36, 40, 63, 65, 66, 75, 77, 78, 83, 87, 89]

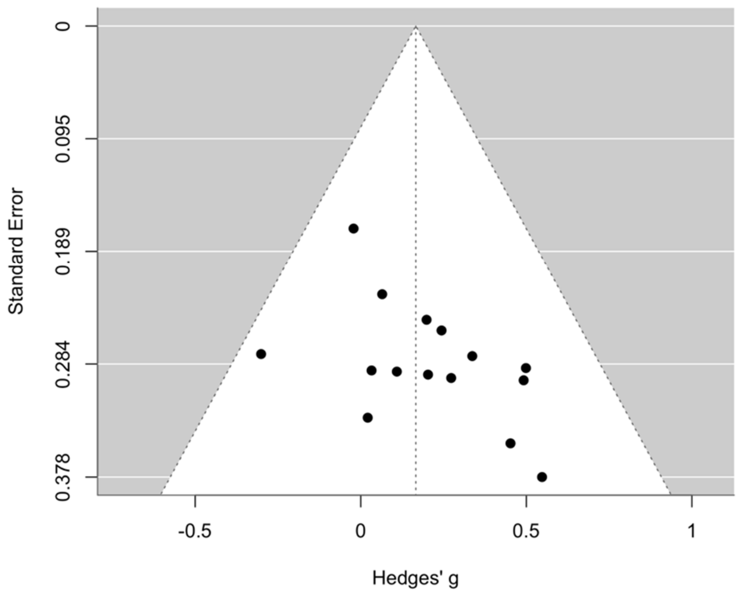

Figure S5: Funnel plot for performance time [2, 3, 5, 6, 8, 9, 12, 17, 18, 23, 25-28, 31, 33, 37, 39, 40, 41, 44, 47, 48, 59, 61, 64, 71-74, 76, 79, 80, 85, 88]

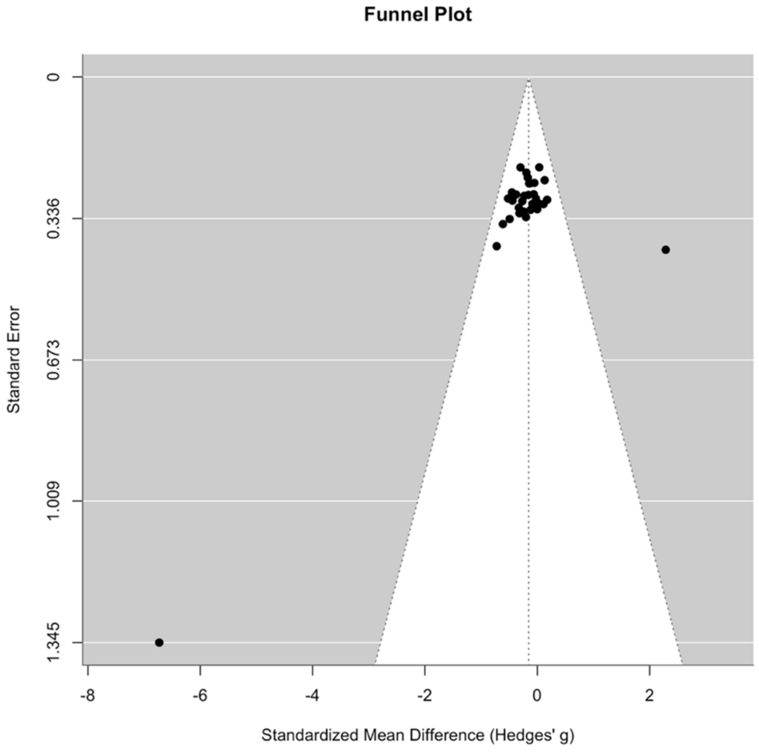

Figure S6: Forest plot for power output [1, 3, 5, 11, 12, 15, 17, 19-21, 26, 31, 38, 41, 42, 47, 49, 54, 58, 59, 60, 64, 67, 70-73, 81-86, 90]

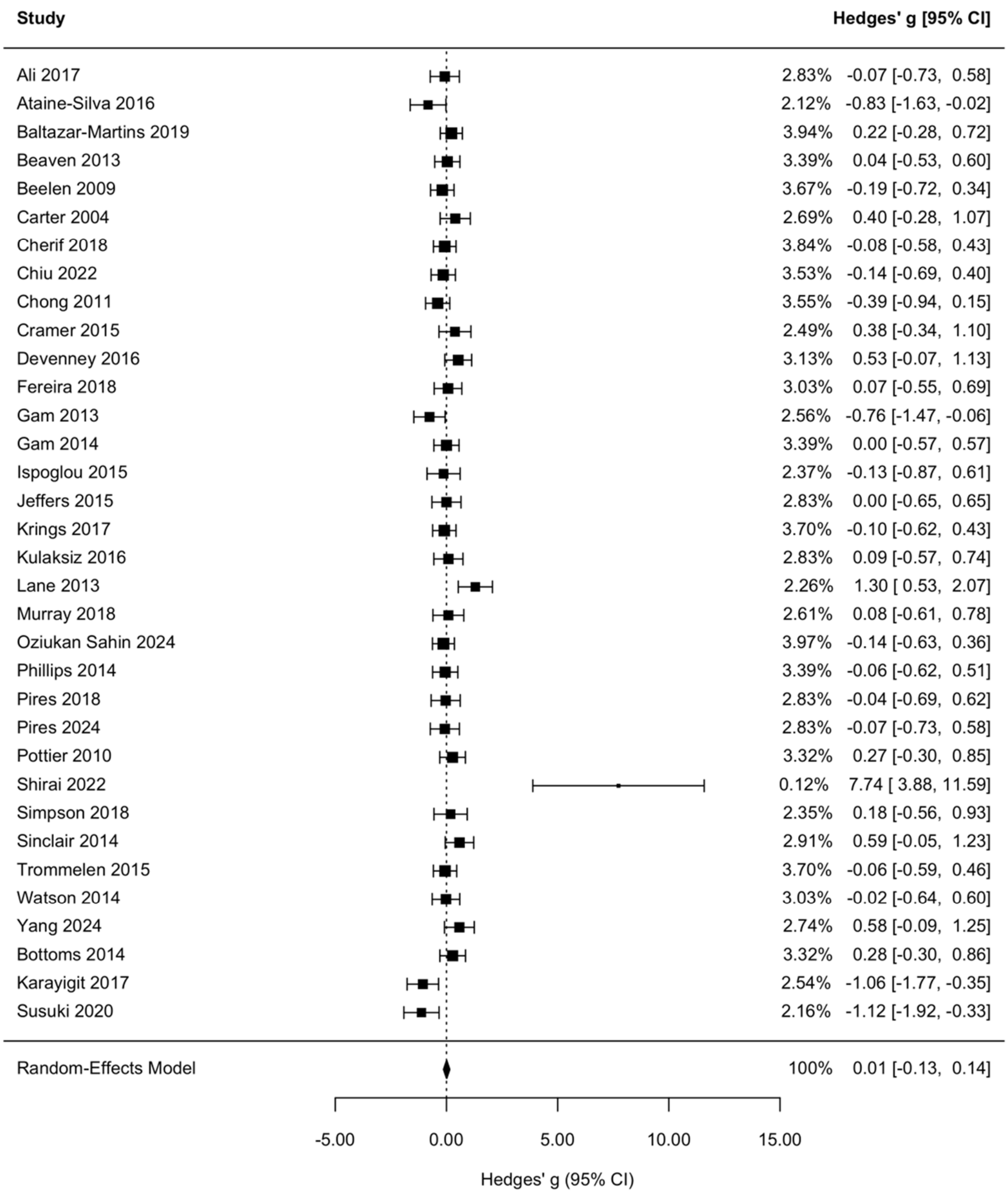

Figure S7: Funnel plot for power output [1, 3, 5, 11, 12, 15, 17, 19-21, 26, 31, 38, 41, 42, 47, 49, 54, 58, 59, 60, 64, 67, 70-73, 81-86, 90]

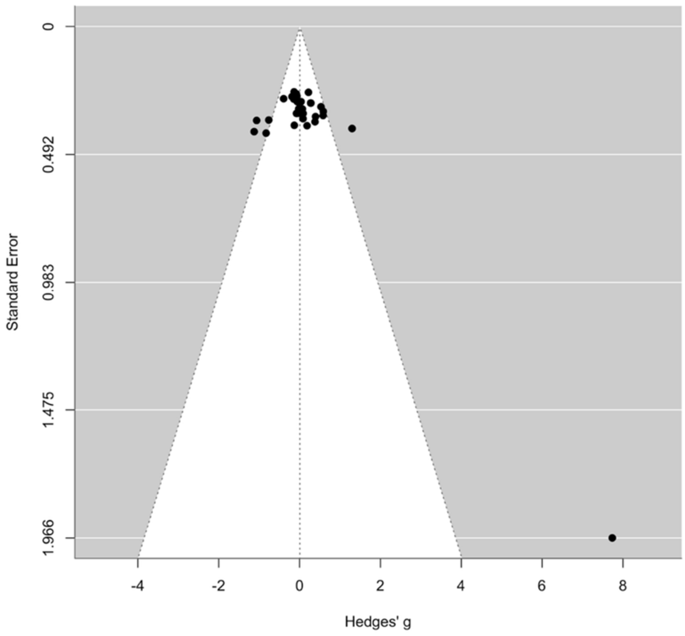

Figure S8: Forest plot for VO_2max [3, 8, 40, 47, 62, 64, 72, 75, 78, 89]

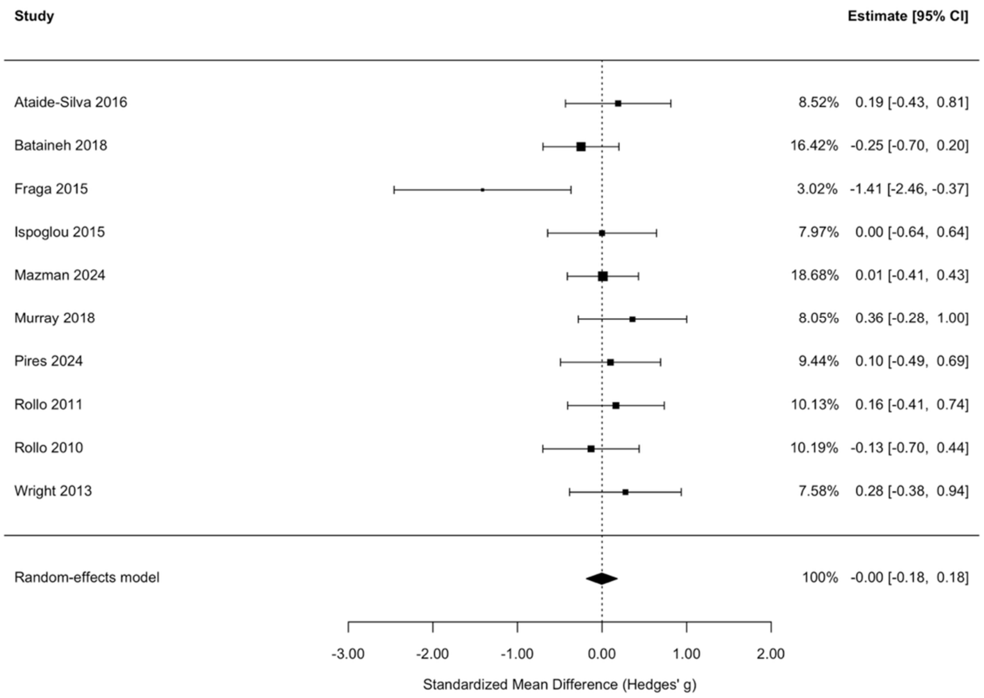

Figure S9: Funnel plot for VO_2max [3, 8, 40, 47, 62, 64, 72, 75, 78, 89]

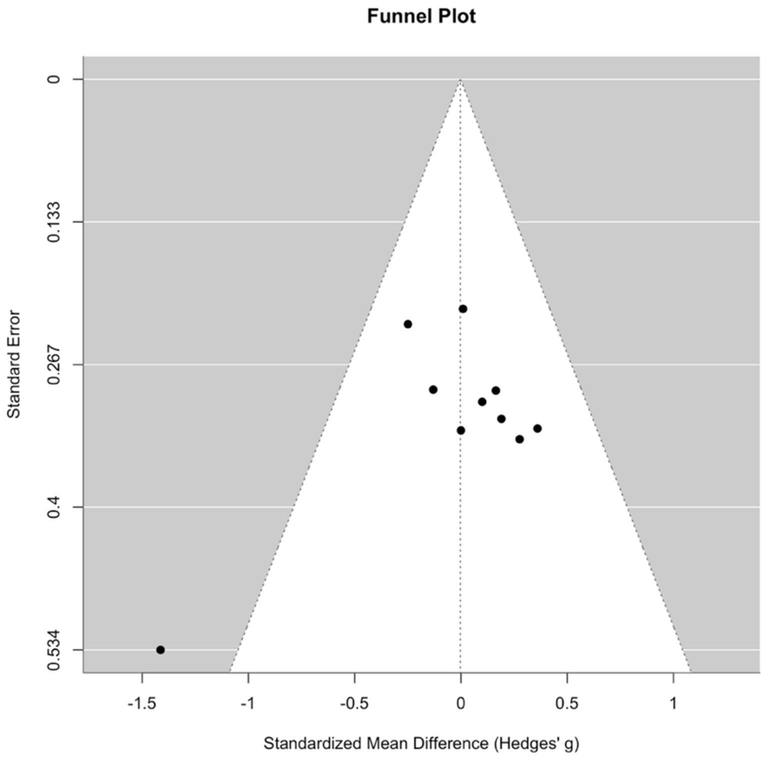

Figure S10: Funnel plot for speed [8, 15, 19, 22, 23, 25, 26, 31, 33, 39-41, 59, 63, 64, 66, 71, 74-78, 83, 87]

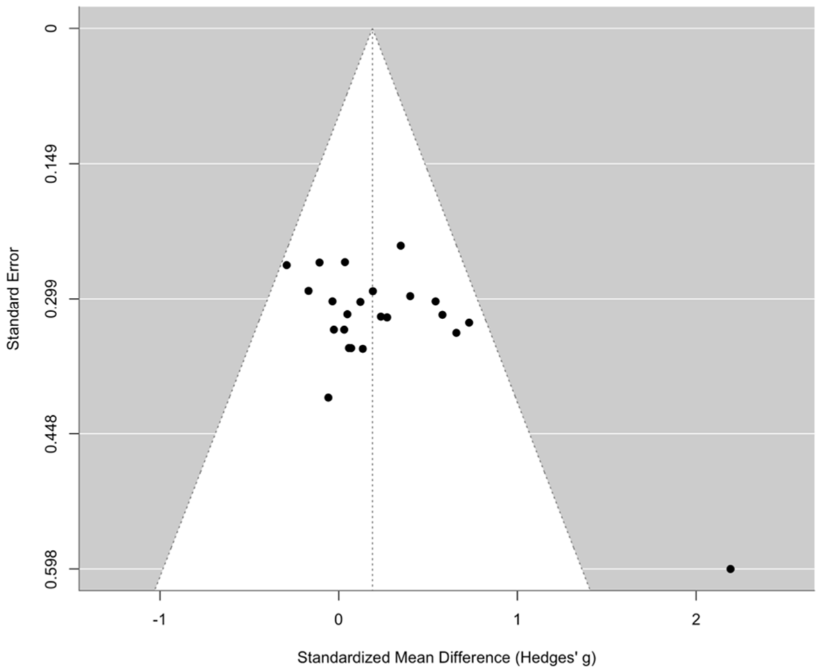

Figure S11: Funnel plot for workload [7, 10, 24, 29, 34, 35, 53, 58, 86, 90]

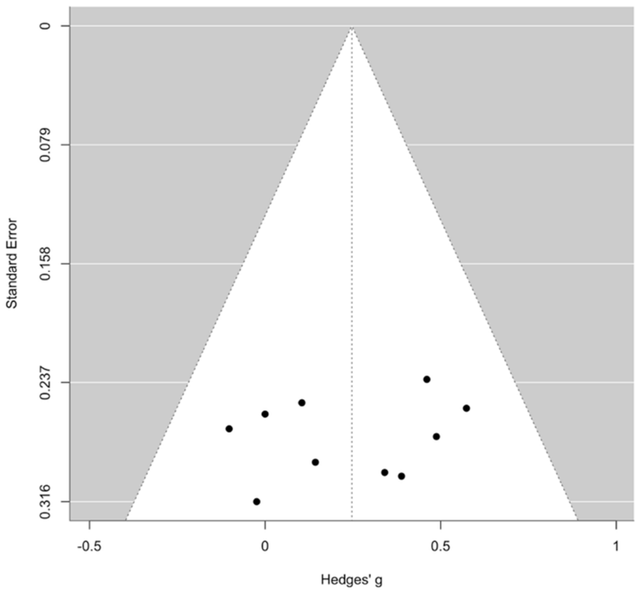

Figure S12: Funnel plot for MVC [4, 10, 13, 14, 23, 46, 49, 50, 57, 61, 68]

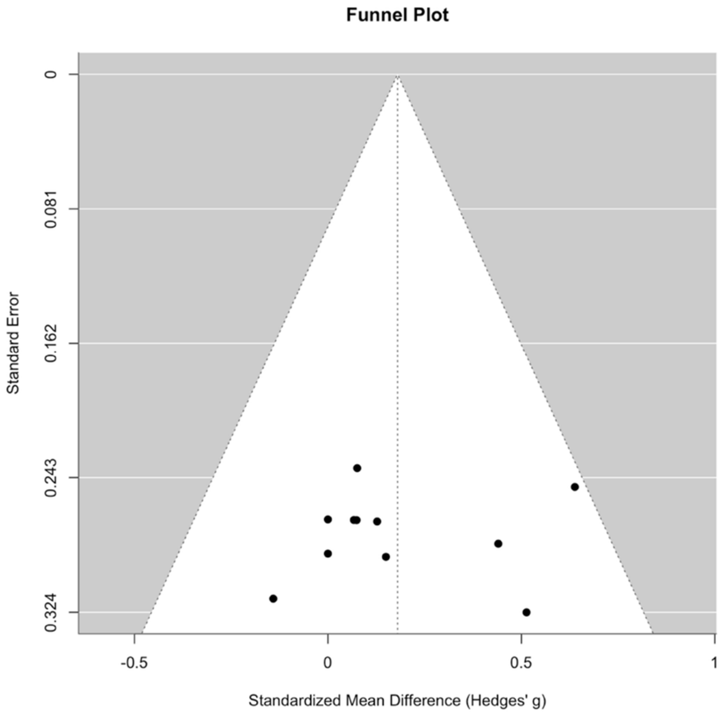

Table S3: PRISMA checklist

Section and Topic	Item #	Checklist item	Location where item is reported
**TITLE**			
Title	1	Identify the report as a systematic review.	Page 1
**ABSTRACT**			
Abstract	2	See the PRISMA 2020 for Abstracts checklist.	Page 1
**INTRODUCTION**			
Rationale	3	Describe the rationale for the review in the context of existing knowledge.	Page 2
Objectives	4	Provide an explicit statement of the objective(s) or question(s) the review addresses.	Page 2
**METHODS**			
Eligibility criteria	5	Specify the inclusion and exclusion criteria for the review and how studies were grouped for the syntheses.	Page 3
Information sources	6	Specify all databases, registers, websites, organisations, reference lists and other sources searched or consulted to identify studies. Specify the date when each source was last searched or consulted.	Page 3
Search strategy	7	Present the full search strategies for all databases, registers and websites, including any filters and limits used.	Page 3, Supplement
Selection process	8	Specify the methods used to decide whether a study met the inclusion criteria of the review, including how many reviewers screened each record and each report retrieved, whether they worked independently, and if applicable, details of automation tools used in the process.	Page 3
Data collection process	9	Specify the methods used to collect data from reports, including how many reviewers collected data from each report, whether they worked independently, any processes for obtaining or confirming data from study investigators, and if applicable, details of automation tools used in the process.	Pages 3, 4
Data items	10a	List and define all outcomes for which data were sought. Specify whether all results that were compatible with each outcome domain in each study were sought (e.g. for all measures, time points, analyses), and if not, the methods used to decide which results to collect.	Pages 4, 5
	10b	List and define all other variables for which data were sought (e.g. participant and intervention characteristics, funding sources). Describe any assumptions made about any missing or unclear information.	Page 4
Study risk of bias assessment	11	Specify the methods used to assess risk of bias in the included studies, including details of the tool(s) used, how many reviewers assessed each study and whether they worked independently, and if applicable, details of automation tools used in the process.	Page 4
Effect measures	12	Specify for each outcome the effect measure(s) (e.g. risk ratio, mean difference) used in the synthesis or presentation of results.	Pages 4, 5
Synthesis	13a	Describe the processes used to decide which studies were eligible for each synthesis (e.g. tabulating the study intervention characteristics and	Page 5,

Section and Topic	Item #	Checklist item	Location where item is reported
methods		comparing against the planned groups for each synthesis (item #5)).	Supplement
	13b	Describe any methods required to prepare the data for presentation or synthesis, such as handling of missing summary statistics, or data conversions.	Pages 4, 5
	13c	Describe any methods used to tabulate or visually display results of individual studies and syntheses.	Pages 4, 5
	13d	Describe any methods used to synthesize results and provide a rationale for the choice(s). If meta-analysis was performed, describe the model(s), method(s) to identify the presence and extent of statistical heterogeneity, and software package(s) used.	Pages 4, 5
	13e	Describe any methods used to explore possible causes of heterogeneity among study results (e.g. subgroup analysis, meta-regression).	Page 5
	13f	Describe any sensitivity analyses conducted to assess robustness of the synthesized results.	Pages 4, 5
Reporting bias assessment	14	Describe any methods used to assess risk of bias due to missing results in a synthesis (arising from reporting biases).	Page 4
Certainty assessment	15	Describe any methods used to assess certainty (or confidence) in the body of evidence for an outcome.	Page 5
**RESULTS**			
Study selection	16a	Describe the results of the search and selection process, from the number of records identified in the search to the number of studies included in the review, ideally using a flow diagram.	Pages 5, 6, Figure 1
	16b	Cite studies that might appear to meet the inclusion criteria, but which were excluded, and explain why they were excluded.	Figure 1
Study characteristics	17	Cite each included study and present its characteristics.	Page 6, supplement
Risk of bias in studies	18	Present assessments of risk of bias for each included study.	Pages 6-7
Results of individual studies	19	For all outcomes, present, for each study: (a) summary statistics for each group (where appropriate) and (b) an effect estimate and its precision (e.g. confidence/credible interval), ideally using structured tables or plots.	Pages 7-14
Results of syntheses	20a	For each synthesis, briefly summarise the characteristics and risk of bias among contributing studies.	Page 7
	20b	Present results of all statistical syntheses conducted. If meta-analysis was done, present for each the summary estimate and its precision (e.g. confidence/credible interval) and measures of statistical heterogeneity. If comparing groups, describe the direction of the effect.	Pages 7-14
	20c	Present results of all investigations of possible causes of heterogeneity among study results.	Pages 7-14
	20d	Present results of all sensitivity analyses conducted to assess the robustness of the synthesized results.	Pages 7-14

Section and Topic	Item #	Checklist item	Location where item is reported
Reporting biases	21	Present assessments of risk of bias due to missing results (arising from reporting biases) for each synthesis assessed.	Pages 7-14
Certainty of evidence	22	Present assessments of certainty (or confidence) in the body of evidence for each outcome assessed.	Page 13, Table 1
**DISCUSSION**			
Discussion	23a	Provide a general interpretation of the results in the context of other evidence.	Pages 16-18
	23b	Discuss any limitations of the evidence included in the review.	Page 18
	23c	Discuss any limitations of the review processes used.	Page 18
	23d	Discuss implications of the results for practice, policy, and future research.	Page 19
**OTHER INFORMATION**			
Registration and protocol	24a	Provide registration information for the review, including register name and registration number, or state that the review was not registered.	Page 3
	24b	Indicate where the review protocol can be accessed, or state that a protocol was not prepared.	Pages 3, 18
	24c	Describe and explain any amendments to information provided at registration or in the protocol.	Page 18
Support	25	Describe sources of financial or non-financial support for the review, and the role of the funders or sponsors in the review.	Page 19
Competing interests	26	Declare any competing interests of review authors.	Page 19
Availability of data, code and other materials	27	Report which of the following are publicly available and where they can be found: template data collection forms; data extracted from included studies; data used for all analyses; analytic code; any other materials used in the review.	Page 19

Table S4: GRADE analysis

	Summary of findings table accrding to GRADE analysis						Evaluation components to lower quality						Evaluation components to higher quality		
**No**	**Outcome**	**Standardized or mean difference (95% CI)**	**Number of Participants Intervention group**	**Number of Participants Control**	**Quality of evidence (GRADE)**	**Comments**	Methodological design start point	Risk of bias	Inconsistency of results	Indirectness	Imprecision	Publication bias	Large effect	Dose response	Confounding
1	Muscular endurance	SMD: 0.49, CI:-0.31, 1.29	165	165	Moderate ⊕⊕⊕◯	We are moderately confident in the effect estimate: The true effect is likely to be close to the estimate of the effect, but there is a possibility that it is substantially different	RCT or CT blinded designs: High quality	>50% low risk of bias. No downgrade	Even though we used a random effect model meta-analysis, we consider >75% heterogeneity as an index of inconsistency. I2=95.7%, substantial heterogeneity. Downgrade one level	All of the studies do display as a primary aim, very similar to the systematic review aim. Therefore, the available evidence is applicable to our research question. No downgrade	CI crosses one important-effect threshold (serious). The overall sample size is 165, which based on the effect size magnitude meet 100% requirements of the optimal sample size. The optimal sample size was calculated as twice the squared sum of the critical values for statistical significance and power, divided by the squared SMD. No downgrade	The studies in this meta-analysis do not suffer from important limitations, the evidence is direct and consistent. No major funding from the industry. Funnel-plot asymmetry innapropriate due to only nine entries are invovlved in the meta-analysis. No downgrade	SMD=0.49 (small). No upgrade	No evidence for a dose response effect. No upgrade	We found no confounding factors that indicate upgrading

No	Summary of findings table accrding to GRADE analysis						Evaluation components to lower quality						Evaluation components to higher quality		
No	Outcome	Standardized or mean difference (95% CI)	Number of Participants Intervention group	Number of Participants Control	Quality of evidence (GRADE)	Comments	Methodological design start point	Risk of bias	Inconsistency of results	Indirectness	Imprecision	Publication bias	Large effect	Dose response	Confounding
2	Maximum Volitional Contraction	SMD: 0.18, CI: 0.02, 0.34	131	131	Moderate ⊕⊕⊕◯	We are very confident that the true effect lies close to that of the estimate of the effect.	RCT or CT blinded designs: High quality	>50% low risk of bias. No downgrade	Even though we used a random effect model meta-analysis, we consider >75% heterogeneity as an index of inconsistency. I2=0%, no heterogeneity. No downgrade	All of the studies do display as a primary aim, very similar to the systematic review aim. Therefore, the available evidence is applicable to our research question. No downgrade	CI lies entirely within one practical region. The overall sample size is 131, which based on the effect size magnitude meet 27% requirements of the optimal sample size. The optimal sample size was calculated as twice the squared sum of the critical values for statistical significance and power, divided by the squared SMD. Downgrade one level	The studies in this meta-analysis do not suffer from important limitations, the evidence is direct and consistent. No major funding from the industry. No publication bias (Egger test p>0.05). No downgrade	SMD=0.18 (trivial). No upgrade	No evidence for a dose response effect. No upgrade	We found no confounding factors that indicate upgrading

	Summary of findings table accrding to GRADE analysis						Evaluation components to lower quality						Evaluation components to higher quality		
**No**	**Outcome**	**Standardized or mean difference (95% CI)**	**Number of Participants Intervention group**	**Number of Participants Control**	**Quality of evidence (GRADE)**	**Comments**	Methodological design start point	Risk of bias	Inconsistency of results	Indirectness	Imprecision	Publication bias	Large effect	Dose response	Confounding
3	Distance covered	SMD: 0.17, CI: 0.03, 0.30	187	187	Moderate ⊕⊕⊕◯	We are very confident that the true effect lies close to that of the estimate of the effect.	RCT or CT blinded designs: High quality	>50% low risk of bias. No downgrade	Even though we used a random effect model meta-analysis, we consider >75% heterogeneity as an index of inconsistency. I2=0%, no heterogeneity. No downgrade	All of the studies do display as a primary aim, very similar to the systematic review aim. Therefore, the available evidence is applicable to our research question. No downgrade	CI lies entirely within one practical region. The overall sample size is 187, which based on the effect size magnitude meet 34% requirements of the optimal sample size. The optimal sample size was calculated as twice the squared sum of the critical values for statistical significance and power, divided by the squared SMD. Downgrade one level	The studies in this meta-analysis do not suffer from important limitations, the evidence is direct and consistent. No major funding from the industry. No publication bias (Egger test p>0.05). No downgrade	SMD=0.17 (trivial). No upgrade	No evidence for a dose response effect. No upgrade	We found no confounding factors that indicate upgrading

	Summary of findings table accrding to GRADE analysis						Evaluation components to lower quality						Evaluation components to higher quality		
**No**	**Outcome**	**Standardized or mean difference (95% CI)**	**Number of Participants Intervention group**	**Number of Participants Control**	**Quality of evidence (GRADE)**	**Comments**	Methodological design start point	Risk of bias	Inconsistency of results	Indirectness	Imprecision	Publication bias	Large effect	Dose response	Confounding
4	Performance time	SMD: -0.15, CI: -0.25, -0.05	404	404	Moderate ⊕⊕⊕◯	We are moderately confident in the effect estimate: The true effect is likely to be close to the estimate of the effect, but there is a possibility that it is substantially different	RCT or CT blinded designs: High quality	>50% low risk of bias. No downgrade	Even though we used a random effect model meta-analysis, we consider >75% heterogeneity as an index of inconsistency. I2=9.3%, low heterogeneity. No downgrade	All of the studies do display as a primary aim, very similar to the systematic review aim. Therefore, the available evidence is applicable to our research question. No downgrade	CI lies entirely within one practical region. The overall sample size is 404, which based on the effect size magnitude meet >50% requirements of the optimal sample size. The optimal sample size was calculated as twice the squared sum of the critical values for statistical significance and power, divided by the squared SMD. No downgrade	The studies in this meta-analysis do not suffer from important limitations, the evidence is direct and consistent. No major funding from the industry. Publication bias (Egger test p<0.01). Downgrade one level	SMD=-0.15 (trivial). No upgrade	No evidence for a dose response effect. No upgrade	We found no confounding factors that indicate upgrading

	Summary of findings table accrding to GRADE analysis						Evaluation components to lower quality						Evaluation components to higher quality		
**No**	**Outcome**	**Standardized or mean difference (95% CI)**	**Number of Participants Intervention group**	**Number of Participants Control**	**Quality of evidence (GRADE)**	**Comments**	Methodological design start point	Risk of bias	Inconsistency of results	Indirectness	Imprecision	Publication bias	Large effect	Dose response	Confounding
5	Speed	SMD: 0.17, CI: 0.05, 0.30	260	260	Moderate ⊕⊕⊕◯	We are moderately confident in the effect estimate: The true effect is likely to be close to the estimate of the effect, but there is a possibility that it is substantially different	RCT or CT blinded designs: High quality	>50% low risk of bias. No downgrade	Even though we used a random effect model meta-analysis, we consider >75% heterogeneity as an index of inconsistency. I2=0%, no heterogeneity. No downgrade	All of the studies do display as a primary aim, very similar to the systematic review aim. Therefore, the available evidence is applicable to our research question. No downgrade	CI lies entirely within one practical region. The overall sample size is 260, which based on the effect size magnitude meet >50% requirements of the optimal sample size. The optimal sample size was calculated as twice the squared sum of the critical values for statistical significance and power, divided by the squared SMD. No downgrade	The studies in this meta-analysis do not suffer from important limitations, the evidence is direct and consistent. No major funding from the industry. Publication bias (Egger test p=0.01). Downgrade one level	SMD=0.17 (trivial). No upgrade	No evidence for a dose response effect. No upgrade	We found no confounding factors that indicate upgrading

No	Summary of findings table accrding to GRADE analysis						Evaluation components to lower quality						Evaluation components to higher quality		
No	Outcome	Standardized or mean difference (95% CI)	Number of Participants Intervention group	Number of Participants Control	Quality of evidence (GRADE)	Comments	Methodological design start point	Risk of bias	Inconsistency of results	Indirectness	Imprecision	Publication bias	Large effect	Dose response	Confounding
6	Workload	SMD: 0.25, CI: 0.08, 0.41	144	144	High ⊕⊕⊕⊕	We are very confident that the true effect lies close to that of the estimate of the effect.	RCT or CT blinded designs: High quality	>50% low risk of bias. No downgrade	Even though we used a random effect model meta-analysis, we consider >75% heterogeneity as an index of inconsistency. I2=0%, no heterogeneity. No downgrade	All of the studies do display as a primary aim, very similar to the systematic review aim. Therefore, the available evidence is applicable to our research question. No downgrade	CI lies entirely within one practical region. The overall sample size is 144, which based on the effect size magnitude meet >50% requirements of the optimal sample size. The optimal sample size was calculated as twice the squared sum of the critical values for statistical significance and power, divided by the squared SMD. No downgrade	The studies in this meta-analysis do not suffer from important limitations, the evidence is direct and consistent. No major funding from the industry. No publication bias (Egger test p>0.05). No downgrade	SMD=0.25 (small). No upgrade	No evidence for a dose response effect. No upgrade	We found no confounding factors that indicate upgrading

No	Summary of findings table accrding to GRADE analysis						Evaluation components to lower quality						Evaluation components to higher quality		
No	Outcome	Standardized or mean difference (95% CI)	Number of Participants Intervention group	Number of Participants Control	Quality of evidence (GRADE)	Comments	Methodological design start point	Risk of bias	Inconsistency of results	Indirectness	Imprecision	Publication bias	Large effect	Dose response	Confounding
7	Power output	SMD: 0.008, CI: -0.13, 0.14	373	373	Low ⊕⊕◯◯	We are moderately confident in the effect estimate: The true effect is likely to be close to the estimate of the effect, but there is a possibility that it is substantially different	RCT or CT blinded designs: High quality	>50% low risk of bias. No downgrade	Even though we used a random effect model meta-analysis, we consider >75% heterogeneity as an index of inconsistency. I2=36.1%, no heterogeneity. No downgrade	All of the studies do display as a primary aim, very similar to the systematic review aim. Therefore, the available evidence is applicable to our research question. No downgrade	CI crosses one important-effect threshold (serious). The overall sample size is 373, which based on the effect size magnitude meet only 15% requirements of the optimal sample size. The optimal sample size was calculated as twice the squared sum of the critical values for statistical significance and power, divided by the squared SMD. Downgrade one level	The studies in this meta-analysis do not suffer from important limitations, the evidence is direct and consistent. No major funding from the industry. Publication bias (Egger test p<0.01). Downgrade one level	SMD=0.008 (trivial). No upgrade	No evidence for a dose response effect. No upgrade	We found no confounding factors that indicate upgrading

No	Summary of findings table accrding to GRADE analysis						Evaluation components to lower quality						Evaluation components to higher quality		
No	Outcome	Standardized or mean difference (95% CI)	Number of Participants Intervention group	Number of Participants Control	Quality of evidence (GRADE)	Comments	Methodological design start point	Risk of bias	Inconsistency of results	Indirectness	Imprecision	Publication bias	Large effect	Dose response	Confounding
8	VO_2max	SMD: -0.003, CI: -0.18, 0.18	103	103	Moderate ⊕⊕⊕◯	We are very confident that the true effect lies close to that of the estimate of the effect.	RCT or CT blinded designs: High quality	>50% low risk of bias. No downgrade	Even though we used a random effect model meta-analysis, we consider >75% heterogeneity as an index of inconsistency. I2=0%, no heterogeneity. No downgrade	All of the studies do display as a primary aim, very similar to the systematic review aim. Therefore, the available evidence is applicable to our research question. No downgrade	CI crosses one important-effect threshold (serious). The overall sample size is 103, which based on the effect size magnitude meet <1% requirements of the optimal sample size. The optimal sample size was calculated as twice the squared sum of the critical values for statistical significance and power, divided by the squared SMD. Downgrade one level	The studies in this meta-analysis do not suffer from important limitations, the evidence is direct and consistent. No major funding from the industry. Funnel-plot asymmetry innapropriate due to only nine entries are invovlved in the meta-analysis. No downgrade	SMD=-0.003 (trivial). No upgrade	No evidence for a dose response effect. No upgrade	We found no confounding factors that indicate upgrading

References in the supplement

1. Ali, A.; Moss, C.; Yoo, M. J. Y.; Wilkinson, A.; Breier, B. H. Effect of mouth rinsing and ingestion of carbohydrate solutions on mood and perceptual responses during exercise. *J Int Soc Sports Nutr* **2017**, *14*, 4, doi:10.1186/s12970-016-0161-8.
2. Ali, A.; Yoo, M. J.; Moss, C.; Breier, B. H. Carbohydrate mouth rinsing has no effect on power output during cycling in a glycogen-reduced state. *J Int Soc Sports Nutr* **2016**, *13*, 19, doi:10.1186/s12970-016-0131-1.
3. Ataide-Silva, T.; Ghiarone, T.; Bertuzzi, R.; Stathis, C. G.; Leandro, C. G.; Lima-Silva, A. E. CHO Mouth Rinse Ameliorates Neuromuscular Response with Lower Endogenous CHO Stores. *Med Sci Sports Exerc* **2016**, *48*, 1810–1820, doi:10.1249/mss.0000000000000973.
4. Bailey, S. P.; Hibbard, J.; La Forge, D.; Mitchell, M.; Roelands, B.; Harris, G. K.; Folger, S. Impact of a Carbohydrate Mouth Rinse on Quadriceps Muscle Function and Corticomotor Excitability. *Int J Sports Physiol Perform* **2019**, *14*, 927–933, doi:10.1123/ijspp.2018-0583.
5. Baltazar-Martins, G.; Del Coso, J. Carbohydrate Mouth Rinse Decreases Time to Complete a Simulated Cycling Time Trial. *Front Nutr* **2019**, *6*, 65, doi:10.3389/fnut.2019.00065.
6. Bastos-Silva, V. J.; Melo Ade, A.; Lima-Silva, A. E.; Moura, F. A.; Bertuzzi, R.; de Araujo, G. G. Carbohydrate Mouth Rinse Maintains Muscle Electromyographic Activity and Increases Time to Exhaustion during Moderate but not High-Intensity Cycling Exercise. *Nutrients* **2016**, *8*, 49, doi:10.3390/nu8030049.
7. Bastos-Silva, V. J.; Prestes, J.; Geraldes, A. A. R. Effect of Carbohydrate Mouth Rinse on Training Load Volume in Resistance Exercises. *J Strength Cond Res* **2019**, *33*, 1653–1657, doi:10.1519/jsc.0000000000002092.
8. Bataineh, M. F.; Al-Nawaiseh, A. M.; Abu Altaieb, M. H.; Bellar, D. M.; Hindawi, O. S.; Judge, L. W. Impact of carbohydrate mouth rinsing on time to exhaustion during Ramadan: A randomized controlled trial in Jordanian men. *Eur J Sport Sci* **2018**, *18*, 357–366, doi:10.1080/17461391.2017.1420236.
9. Bavaresco Gambassi, B.; Gomes de Santana Barros Leal, Y.; Pinheiro Dos Anjos, E. R.; Antonelli, B. A.; Gomes Gonçalves, E. Silva D. C.; Hermes Pires de Mélo Montenegro, I.; di Cássia de Oliveira Angelo, R.; Suruagy Correia Moura, I.; Schwingel, P. A. Carbohydrate mouth rinse improves cycling performance carried out until the volitional exhaustion. *J Sports Med Phys Fitness* **2019**, *59*, 1–5, doi:10.23736/s0022-4707.17.07980-4.
10. Bazzucchi, I.; Patrizio, F.; Felici, F.; Nicolò, A.; Sacchetti, M. Carbohydrate Mouth Rinsing: Improved Neuromuscular Performance During Isokinetic Fatiguing Exercise. *Int J Sports Physiol Perform* **2017**, *12*, 1031–1038, doi:10.1123/ijspp.2016-0583.
11. Beaven, C. M.; Maulder, P.; Pooley, A.; Kilduff, L.; Cook, C. Effects of caffeine and carbohydrate mouth rinses on repeated sprint performance. *Appl Physiol Nutr Metab* **2013**, *38*, 633–637, doi:10.1139/apnm-2012-0333.
12. Beelen, M.; Berghuis, J.; Bonaparte, B.; Ballak, S. B.; Jeukendrup, A. E.; van Loon, L. J. Carbohydrate mouth rinsing in the fed state: lack of enhancement of time-trial performance. *Int J Sport Nutr Exerc Metab* **2009**, *19*, 400–409, doi:10.1123/ijsnem.19.4.400.
13. Black, C. D.; Haskins, K. R.; Bemben, M. G.; Larson, R. D. Carbohydrate Mouth Rinsing Does Not Alter Central or Peripheral Fatigue After High-Intensity and Low-Intensity Exercise in Men. *J Strength Cond Res* **2022**, *36*, 142–148, doi:10.1519/jsc.0000000000004162.
14. Black, C. D.; Schubert, D. J.; Szczyglowski, M. K.; Wren, J. D. Carbohydrate Mouth Rinsing Does Not Prevent the Decline in Maximal Strength After Fatiguing Exercise. *J Strength Cond Res* **2018**, *32*, 2466–2473, doi:10.1519/jsc.0000000000002444.
15. Bottoms, Lindsay; Hurst, Howard; Scriven, A.; Lynch, Fergal; Bolton, J.; Vercoe, L.; Shone, Z.; Barry, G.; Sinclair, Jonathan. The effect of caffeine mouth rinse on self-paced cycling performance. *Comparative Exercise Physiology* **2014**, *10*, 239–245, doi:10.3920/CEP140015.

16. Bowling, L. R.; Scudamore, E. M.; Church, J. B.; Scudamore, V. The Effects of a Carbohydrate Mouth Rinse on Soccer-Specific Skills Following a Morning High-Intensity Practice. *Int J Exerc Sci* **2025**, *18*, 215–224, doi:10.70252/sjxt6980.
17. Carter, J. M.; Jeukendrup, A. E.; Jones, D. A. The effect of carbohydrate mouth rinse on 1-h cycle time trial performance. *Med Sci Sports Exerc* **2004**, *36*, 2107–2111, doi:10.1249/01.mss.0000147585.65709.6f.
18. Chambers, E. S.; Bridge, M. W.; Jones, D. A. Carbohydrate sensing in the human mouth: effects on exercise performance and brain activity. *J Physiol* **2009**, *587*, 1779–1794, doi:10.1113/jphysiol.2008.164285.
19. Cherif, A.; Meeusen, R.; Ryu, J.; Taylor, L.; Farooq, A.; Kammoun, K.; Fenneni, M. A.; Aziz, A. R.; Roelands, B.; Chamari, K. Repeated-sprints exercise in daylight fasting: carbohydrate mouth rinsing does not affect sprint and reaction time performance. *Biol Sport* **2018**, *35*, 237–244, doi:10.5114/biolsport.2018.77824.
20. Chiu, C. H.; Chen, C. H.; Yang, T. J.; Chou, K. M.; Chen, B. W.; Lin, Z. Y.; Lin, Y. C. Carbohydrate mouth rinsing decreases fatigue index of taekwondo frequency speed of kick test. *Chin J Physiol* **2022**, *65*, 46–50, doi:10.4103/cjp.cjp_99_21.
21. Chong, E.; Guelfi, K. J.; Fournier, P. A. Effect of a carbohydrate mouth rinse on maximal sprint performance in competitive male cyclists. *J Sci Med Sport* **2011**, *14*, 162–167, doi:10.1016/j.jsams.2010.08.003.
22. Chryssanthopoulos, C.; Ziaras, C.; Oosthuyse, T.; Lambropoulos, I.; Giorgios, P. P.; Zacharogiannis, E.; Philippou, A.; Maridaki, M. Carbohydrate mouth rinse does not affect performance during a 60-min running race in women. *J Sports Sci* **2017**, *36*, 824–833, doi:10.1080/02640414.2017.1344358.
23. Clarke, N. D.; Hammond, S.; Kornilios, E.; Mundy, P. D. Carbohydrate mouth rinse improves morning high-intensity exercise performance. *Eur J Sport Sci* **2017**, *17*, 955–963, doi:10.1080/17461391.2017.1333159.
24. Clarke, N. D.; Kornilios, E.; Richardson, D. L. Carbohydrate and Caffeine Mouth Rinses Do Not Affect Maximum Strength and Muscular Endurance Performance. *J Strength Cond Res* **2015**, *29*, 2926–2931, doi:10.1519/jsc.0000000000000945.
25. Clarke, N. D.; Thomas, J. R.; Kagka, M.; Ramsbottom, R.; Delextrat, A. No Dose-Response Effect of Carbohydrate Mouth Rinse Concentration on 5-km Running Performance in Recreational Athletes. *J Strength Cond Res* **2017**, *31*, 715–720, doi:10.1519/jsc.0000000000001531.
26. Cramer, M. N.; Thompson, M. W.; Périard, J. D. Thermal and Cardiovascular Strain Mitigate the Potential Benefit of Carbohydrate Mouth Rinse During Self-Paced Exercise in the Heat. *Front Physiol* **2015**, *6*, 354, doi:10.3389/fphys.2015.00354.
27. de Oliveira, José Jonas; de Souza Salles, Allan Henrique; Reis Barbosa, Carolina Gabriela; de Oliveira Silva, Jasiele Aparecida; de Melo Dias, Ricardo; Silva Vilela Ribeiro, Anna Gabriela; Vieira Lacerda, Fábio; da Silva, Jean Marinho; de Souza e Silva, Alexandre. Effect of Post-Activation Potentiation and Carbohydrate Mouth Rise on Repeated Sprint Ability in University Futsal Players. *Journal of Exercise Physiology Online* **2020**, *23*, 29–40.
28. De Oliveira, OsÉ Jonas; Verlengia, Rozangela; Barbosa, Carolina Gabriela Reis; Sindorf, Marcio Antonio Gonsalves; Da Rocha, Guilherme Luiz; Lopes, Charles Ricardo; Crisp, Alex H. Effects of post-activation potentiation and carbohydrate mouth rinse on repeated sprint ability. *Journal of Human Sport & Exercise* **2019**, *14*, 159–169, doi:10.14198/jhse.2019.141.13.
29. Decimoni, Luciana S.; Curty, Victor M.; Almeida, Livia; Koch, Alexander J.; Willardson, Jeffrey M.; Machado, Marco. Carbohydrate mouth rinsing improves resistance training session performance. *International Journal of Sports Science & Coaching* **2018**, *13*, 804–809, doi:10.1177/1747954118755640.
30. Deighton, K.; Duckworth, L.; Matu, J.; Suter, M.; Fletcher, C.; Stead, S.; Ali, S.; Gunby, N.; Korsness, K. Mouth rinsing with a sweet solution increases energy expenditure and decreases appetite during 60 min of self-regulated walking exercise. *Appl Physiol Nutr Metab* **2016**, *41*, 1255–1261, doi:10.1139/apnm-2016-0344.

31. Devenney, S.; Collins, K.; Shortall, M. Effects of various concentrations of carbohydrate mouth rinse on cycling performance in a fed state. *Eur J Sport Sci* **2016**, *16*, 1073–1078, doi:10.1080/17461391.2016.1196735.
32. Dolan, P.; Witherbee, K. E.; Peterson, K. M.; Kerksick, C. M. Effect of Carbohydrate, Caffeine, and Carbohydrate + Caffeine Mouth Rinsing on Intermittent Running Performance in Collegiate Male Lacrosse Athletes. *J Strength Cond Res* **2017**, *31*, 2473–2479, doi:10.1519/jsc.0000000000001819.
33. Dorling, J. L.; Earnest, C. P. Effect of carbohydrate mouth rinsing on multiple sprint performance. *J Int Soc Sports Nutr* **2013**, *10*, 41, doi:10.1186/1550-2783-10-41.
34. Dunkin, J. E.; Phillips, S. M. The Effect of a Carbohydrate Mouth Rinse on Upper-Body Muscular Strength and Endurance. *J Strength Cond Res* **2017**, *31*, 1948–1953, doi:10.1519/jsc.0000000000001668.
35. Durkin, M.; Akeroyd, H.; Holliday, A. Carbohydrate mouth rinse improves resistance exercise capacity in the glycogen-lowered state. *Appl Physiol Nutr Metab* **2021**, *46*, 126–132, doi:10.1139/apnm-2020-0298.
36. Evans, E. S.; Brophy, T.; Braswell, M. R.; Boyle, J.; Harris, G. K.; Watkins, R. H.; Bailey, S. P. Carbohydrate mouth rinsing does not affect 6-min walk test performance and blood glucose responses in older adults. *Eur J Appl Physiol* **2021**, *121*, 2849–2858, doi:10.1007/s00421-021-04753-x.
37. Fares, E. J.; Kayser, B. Carbohydrate mouth rinse effects on exercise capacity in pre- and postprandial States. *J Nutr Metab* **2011**, *2011*, 385962, doi:10.1155/2011/385962.
38. Ferreira, A. M. J.; Farias-Junior, L. F.; Mota, T. A. A.; Elsangedy, H. M.; Marcadenti, A.; Lemos, Tmam; Okano, A. H.; Fayh, A. P. T. Carbohydrate Mouth Rinse and Hydration Strategies on Cycling Performance in 30 Km Time Trial: A Randomized, Crossover, Controlled Trial. *J Sports Sci Med* **2018**, *17*, 181–187.
39. Ferreira, A. M. J.; Farias-Junior, L. F.; Mota, T. A. A.; Elsangedy, H. M.; Marcadenti, A.; Lemos, Tmam; Okano, A. H.; Fayh, A. P. T. The effect of carbohydrate mouth rinse on performance, biochemical and psychophysiological variables during a cycling time trial: a crossover randomized trial. *J Int Soc Sports Nutr* **2018**, *15*, 23, doi:10.1186/s12970-018-0225-z.
40. Fraga, C.; Velasques, B.; Koch, A. J.; Machado, M.; Paulucio, D.; Ribeiro, P.; Pompeu, F. A. Carbohydrate mouth rinse enhances time to exhaustion during treadmill exercise. *Clin Physiol Funct Imaging* **2015**, *37*, 17–22, doi:10.1111/cpf.12261.
41. Gam, S.; Guelfi, K. J.; Fournier, P. A. Opposition of carbohydrate in a mouth-rinse solution to the detrimental effect of mouth rinsing during cycling time trials. *Int J Sport Nutr Exerc Metab* **2013**, *23*, 48–56, doi:10.1123/ijsnem.23.1.48.
42. Gam, S.; Tan, M.; Guelfi, K. J.; Fournier, P. A. Mouth rinsing with a bitter solution without ingestion does not improve sprint cycling performance. *Eur J Appl Physiol* **2015**, *115*, 129–138, doi:10.1007/s00421-014-2987-6.
43. Green, M. S.; Kimmel, C. S.; Martin, T. D.; Mouser, J. G.; Brune, M. P. Effect of Carbohydrate Mouth Rinse on Resistance Exercise Performance. *J Strength Cond Res* **2022**, *36*, 1916–1921, doi:10.1519/jsc.0000000000003755.
44. Hawkins, K. R.; Krishnan, S.; Ringos, L.; Garcia, V.; Cooper, J. A. Running Performance With Nutritive and Nonnutritive Sweetened Mouth Rinses. *Int J Sports Physiol Perform* **2017**, *12*, 1105–1110, doi:10.1123/ijspp.2016-0577.
45. Hirata, Yoko; Wadazumi, Tsuyoshi; Hamada, Naomi; Shirai, Asako; Watanabe, Hitoshi. The effect of carbohydrate mouth rinse in isometric hand grip performance. *Japanese Journal of Physical Fitness and Sports Medicine* **2021**, *70*, 269–276, doi:10.7600/jspfsm.70.269.
46. Hsu, T. Y.; Hsieh, M. H.; Shiu, Y. J.; Chiu, C. H. Carbohydrate Mouth Rinsing Improves Softball Launch Angle Consistency: A Double-Blind Crossover Study. *Nutrients* **2025**, *17*, doi:10.3390/nu17010167.

47. Ispoglou, T.; O'Kelly, D.; Angelopoulou, A.; Bargh, M.; O'Hara, J. P.; Duckworth, L. C. Mouth Rinsing With Carbohydrate Solutions at the Postprandial State Fail to Improve Performance During Simulated Cycling Time Trials. *J Strength Cond Res* **2015**, *29*, 2316–2325, doi:10.1519/jsc.0000000000000882.
48. James, R. M.; Ritchie, S.; Rollo, I.; James, L. J. No Dose Response Effect of Carbohydrate Mouth Rinse on Cycling Time-Trial Performance. *Int J Sport Nutr Exerc Metab* **2017**, *27*, 25–31, doi:10.1123/ijsnem.2016-0111.
49. Jeffers, R.; Shave, R.; Ross, E.; Stevenson, E. J.; Goodall, S. The effect of a carbohydrate mouth-rinse on neuromuscular fatigue following cycling exercise. *Appl Physiol Nutr Metab* **2015**, *40*, 557–564, doi:10.1139/apnm-2014-0393.
50. Jensen, M.; Stellingwerff, T.; Klimstra, M. Carbohydrate Mouth Rinse Counters Fatigue Related Strength Reduction. *Int J Sport Nutr Exerc Metab* **2015**, *25*, 252–261, doi:10.1123/ijsnem.2014-0061.
51. Karayigit, R.; Ali, A.; Rezaei, S.; Ersoz, G.; Lago-Rodriguez, A.; Domínguez, R.; Naderi, A. Effects of carbohydrate and caffeine mouth rinsing on strength, muscular endurance and cognitive performance. *J Int Soc Sports Nutr* **2021**, *18*, 63, doi:10.1186/s12970-021-00462-0.
52. Karayigit, R.; Eser, M. C.; Gur, F.; Sari, C.; Cepicka, L.; Gabrys, T. Carbohydrate Mouth Rinse Increases High but Not Low Intensity Repetitions to Failure in Resistance-Trained Males. *Nutrients* **2022**, *14*, doi:10.3390/nu14040875.
53. Karayigit, R.; Forbes, S. C.; Naderi, A.; Candow, D. G.; Yildirim, U. C.; Akca, F.; Aras, D.; B, C. Yasli; Sisman, A.; Mor, A.; et al. Different Doses of Carbohydrate Mouth Rinse Have No Effect on Exercise Performance in Resistance Trained Women. *Int J Environ Res Public Health* **2021**, *18*, doi:10.3390/ijerph18073463.
54. Karayigit, Raci; Karabiyik, Hakan; Yaşlı, Burak; Koz, Mitat; Ersoz, Gulfem. The effect of carbohydrate mouth rinse on intermittent sprint performance in soccer players. *Ortadoğu Tıp Dergisi* **2017**, *9*, 183–190, doi:10.21601/ortadogutipdergisi.359888.
55. Karayigit, Raci; Sari, Cengizhan; Yildirim, Ulas; Gürkan, Oğuz; Şişman, Ayşegül; Boz, Hilal; Bayrakdar, Akan. The Effects of Carbohydrate Mouth Rinsing on Upper Body Strength and Muscular Endurance Performance. *Progress in Nutrition* **2021**, *23*, doi:10.23751/pn.v22i4.9333.
56. Karuk, H. N.; Rudarli Nalcakan, G.; Pekünlü, E. Effects of carbohydrate and caffeine combination mouth rinse on anaerobic performance of highly trained male athletes. *Eur J Sport Sci* **2022**, *22*, 589–599, doi:10.1080/17461391.2021.1907449.
57. Khong, T. K.; Selvanayagam, V.; Yusof, A. Effect of glucose and sodium chloride mouth rinses on neuromuscular fatigue: a preliminary study. *Eur J Sport Sci* **2020**, *21*, 224–230, doi:10.1080/17461391.2020.1730980.
58. Krings, B. M.; Peterson, T. J.; Shepherd, B. D.; McAllister, M. J.; Smith, J. W. Effects of Carbohydrate Ingestion and Carbohydrate Mouth Rinse on Repeat Sprint Performance. *Int J Sport Nutr Exerc Metab* **2017**, *27*, 204–212, doi:10.1123/ijsnem.2016-0321.
59. Kulaksız, T. N.; Koşar Ş, N.; Bulut, S.; Güzel, Y.; Willems, M. E.; Hazir, T.; Turnagöl, H. H. Mouth Rinsing with Maltodextrin Solutions Fails to Improve Time Trial Endurance Cycling Performance in Recreational Athletes. *Nutrients* **2016**, *8*, doi:10.3390/nu8050269.
60. Lane, S. C.; Bird, S. R.; Burke, L. M.; Hawley, J. A. Effect of a carbohydrate mouth rinse on simulated cycling time-trial performance commenced in a fed or fasted state. *Appl Physiol Nutr Metab* **2013**, *38*, 134–139, doi:10.1139/apnm-2012-0300.
61. Luden, N. D.; Saunders, M. J.; D'Lugos, A. C.; Pataky, M. W.; Baur, D. A.; Vining, C. B.; Schroer, A. B. Carbohydrate Mouth Rinsing Enhances High Intensity Time Trial Performance Following Prolonged Cycling. *Nutrients* **2016**, *8*, doi:10.3390/nu8090576.
62. Mazman, Bahar; Yetgin, Meral Kucuk. The Effect of Carbonhydrate Mouth Rinse on the Endurance Performance in Football Players. *CBÜ Beden Eğitimi & Spor Bilimleri Dergisi* **2024**, *19*, 95–108, doi:10.33459/cbubesbd.1399602.

63. Muhamad, A. S.; Puad, Nfrm; Kuan, G. Effects of Carbohydrate Mouth Rinsing on Salivary Lysozyme, Mood States and Running Performance Among Recreational Runners. *Malays J Med Sci* **2020**, *27*, 87–96, doi:10.21315/mjms2020.27.1.9.
64. Murray, K. O.; Paris, H. L.; Fly, A. D.; Chapman, R. F.; Mickleborough, T. D. Carbohydrate Mouth Rinse Improves Cycling Time-Trial Performance without Altering Plasma Insulin Concentration. *J Sports Sci Med* **2018**, *17*, 145–152.
65. Nehme, R.; de Branco, F. M. S.; Vieira, P. F.; Guimarães, A. V. C.; Gomes, G. K.; Teixeira, G. P.; Rodrigues, P. H.; de Castro Junior, L. M.; Puga, G. M.; Saunders, B.; et al. Single and Serial Carbohydrate Mouth Rinsing Do Not Improve Yo-Yo Intermittent Recovery Test Performance in Soccer Players. *Int J Sport Nutr Exerc Metab* **2022**, *32*, 22–29, doi:10.1123/ijsnem.2021-0174.
66. Nyman, D. L. E.; Gamble, A. S. D.; Bigg, J. L.; Boyd, L. A.; Vanderheyden, A. J.; Spriet, L. L. Carbohydrate Mouth-Rinsing Improves Overtime Physical Performance in Male Ice Hockey Players During On-Ice Scrimmages. *Front Nutr* **2022**, *9*, 792708, doi:10.3389/fnut.2022.792708.
67. ÖZlÜKan ŞAhİN, Burcu; YÜZbaŞIoĞLu, Yasin; Rudarli, Gülbin. Effects of Carbohydrate and Caffeine Mouth Rinsing Methods on Repetitive Kick Force and Duration, and Hand Reaction Time in Karate Athletes. *Eurasian Journal of Sport Sciences & Education / Avrasya Spor Bilimleri ve Eğitim Dergisi* **2024**, *6*, 181–199, doi:10.47778/ejsse.1515897.
68. Painelli, V. S.; Roschel, H.; Gualano, B.; Del-Favero, S.; Benatti, F. B.; Ugrinowitsch, C.; Tricoli, V.; Lancha, A. H., Jr. The effect of carbohydrate mouth rinse on maximal strength and strength endurance. *Eur J Appl Physiol* **2011**, *111*, 2381–2386, doi:10.1007/s00421-011-1865-8.
69. Pereira, P. E. A.; Azevedo, P.; Azevedo, K.; Azevedo, W.; Machado, M. Caffeine Supplementation or Carbohydrate Mouth Rinse Improves Performance. *Int J Sports Med* **2020**, *42*, 147–152, doi:10.1055/a-1212-0742.
70. Phillips, S. M.; Findlay, S.; Kavaliauskas, M.; Grant, M. C. The Influence of Serial Carbohydrate Mouth Rinsing on Power Output during a Cycle Sprint. *J Sports Sci Med* **2014**, *13*, 252–258.
71. Pires, F. O.; Brietzke, C.; Pinheiro, F. A.; Veras, K.; de Mattos, E. C. T.; Rodacki, A. L. F.; Ugrinowitsch, C. Carbohydrate Mouth Rinse Fails to Improve Four-Kilometer Cycling Time Trial Performance. *Nutrients* **2018**, *10*, doi:10.3390/nu10030342.
72. Pires, F. O.; Pinheiro, F. A.; Brietzke, C.; Franco-Alvarenga, P. E.; Veras, K.; de Matos, E. C. T.; Rodacki, A. L. F.; Ugrinowitsch, C. Carbohydrate mouth rinse failed to reduce central fatigue, lower perceived exertion, and improve performance during incremental exercise. *Front Nutr* **2024**, *11*, 1329074, doi:10.3389/fnut.2024.1329074.
73. Pottier, A.; Bouckaert, J.; Gilis, W.; Roels, T.; Derave, W. Mouth rinse but not ingestion of a carbohydrate solution improves 1-h cycle time trial performance. *Scand J Med Sci Sports* **2010**, *20*, 105–111, doi:10.1111/j.1600-0838.2008.00868.x.
74. Přibyslavská, V.; Scudamore, E. M.; Johnson, S. L.; Green, J. M.; Stevenson Wilcoxson, M. C.; Lowe, J. B.; O'Neal, E. K. Influence of carbohydrate mouth rinsing on running and jumping performance during early morning soccer scrimmaging. *Eur J Sport Sci* **2015**, *16*, 441–447, doi:10.1080/17461391.2015.1020345.
75. Rollo, I.; Cole, M.; Miller, R.; Williams, C. Influence of mouth rinsing a carbohydrate solution on 1-h running performance. *Med Sci Sports Exerc* **2010**, *42*, 798–804, doi:10.1249/MSS.0b013e3181bac6e4.
76. Rollo, I.; Homewood, G.; Williams, C.; Carter, J.; Goosey-Tolfrey, V. L. The Influence of Carbohydrate Mouth Rinse on Self-Selected Intermittent Running Performance. *Int J Sport Nutr Exerc Metab* **2015**, *25*, 550–558, doi:10.1123/ijsnem.2015-0001.
77. Rollo, I.; Williams, C.; Gant, N.; Nute, M. The influence of carbohydrate mouth rinse on self-selected speeds during a 30-min treadmill run. *Int J Sport Nutr Exerc Metab* **2008**, *18*, 585–600, doi:10.1123/ijsnem.18.6.585.

78. Rollo, I.; Williams, C.; Nevill, M. Influence of ingesting versus mouth rinsing a carbohydrate solution during a 1-h run. *Med Sci Sports Exerc* **2011**, *43*, 468–475, doi:10.1249/MSS.0b013e3181f1cda3.
79. Rossato, L. T.; Fernandes, C. T. M.; Vieira, P. F.; de Branco, F. M. S.; Nahas, P. C.; Puga, G. M.; de Oliveira, E. P. No Improvement in Running Time to Exhaustion at 100% VO2max in Recreationally Active Male Runners With a Preexercise Single-Carbohydrate Mouth Rinse. *Int J Sports Physiol Perform* **2019**, *14*, 1178–1183, doi:10.1123/ijspp.2018-0624.
80. Rowlatt, G.; Bottoms, L.; Edmonds, C. J.; Buscombe, R. The effect of carbohydrate mouth rinsing on fencing performance and cognitive function following fatigue-inducing fencing. *Eur J Sport Sci* **2016**, *17*, 433–440, doi:10.1080/17461391.2016.1251497.
81. Shirai, A.; Wadazumi, T.; Hirata, Y.; Hamada, N.; Hongu, N. Carbohydrate Mouth Rinse and Spray Improve Prolonged Exercise Performance in Recreationally Trained Male College Students. *Sports (Basel)* **2022**, *10*, doi:10.3390/sports10040051.
82. Simpson, G. W.; Pritchett, R.; O'Neal, E.; Hoskins, G.; Pritchett, K. Carbohydrate Mouth Rinse Improves Relative Mean Power During Multiple Sprint Performance. *Int J Exerc Sci* **2018**, *11*, 754–763, doi:10.70252/crpx1180.
83. Sinclair, J.; Bottoms, L.; Flynn, C.; Bradley, E.; Alexander, G.; McCullagh, S.; Finn, T.; Hurst, H. T. The effect of different durations of carbohydrate mouth rinse on cycling performance. *Eur J Sport Sci* **2014**, *14*, 259–264, doi:10.1080/17461391.2013.785599.
84. Suzuki, Yoshio; Sato, Kotaro; Arai, Norie; Endo, Shin. Influence of Trehalose Mouth Rinse on Anaerobic and Aerobic Exercise Performance. *Natural product communications* **2020**, *15*, 1–6, doi:10.1177/1934578X20969594.
85. Trommelen, J.; Beelen, M.; Mullers, M.; Gibala, M. J.; van Loon, L. J.; Cermak, N. M. A Sucrose Mouth Rinse Does Not Improve 1-hr Cycle Time Trial Performance When Performed in the Fasted or Fed State. *Int J Sport Nutr Exerc Metab* **2015**, *25*, 576–583, doi:10.1123/ijsnem.2015-0094.
86. Watson, P.; Nichols, D.; Cordery, P. Mouth rinsing with a carbohydrate solution does not influence cycle time trial performance in the heat. *Appl Physiol Nutr Metab* **2014**, *39*, 1064–1069, doi:10.1139/apnm-2013-0413.
87. Whitham, M.; McKinney, J. Effect of a carbohydrate mouthwash on running time-trial performance. *J Sports Sci* **2007**, *25*, 1385–1392, doi:10.1080/02640410601113676.
88. Williamson-Reisdorph, C. M.; Bechke, E. E.; McLester, C.; Buresh, R.; Millard-Stafford, M.; Green, Z.; Rooks, R.; Nickerson, B.; Kliszczewicz, B. M. Carbohydrate Rinse Fails to Enhance Cycling Performance or Alter Metabolic and Autonomic Recovery in Recreational Cyclists. *J Hum Kinet* **2022**, *83*, 109–119, doi:10.2478/hukin-2022-0036.
89. Wright, B. F.; Davison, G. Carbohydrate Mouth Rinse Improves 1.5 h Run Performance: Is There A Dose-Effect? *Int J Exerc Sci* **2013**, *6*, 328–340, doi:10.70252/ssrn3654.
90. Yang, T. J.; Shiu, Y. J.; Chen, C. H.; Yu, S. Y.; Hsu, Y. Y.; Chiu, C. H. Carbohydrate Mouth Rinses before Exercise Improve Performance of Romanian Deadlift Exercise: A Randomized Crossover Study. *Nutrients* **2024**, *16*, doi:10.3390/nu16081248.
